# Regulation of PDH via cysteine oxidation on DLAT

**DOI:** 10.1038/s41467-026-78266-6

**Published:** 2026-10-14

**Authors:** Fabiola Garcia-Cortizo, Tobias Weber, Hana Nůsková, Daniel Pfaff, Michael Büttner, Angela Wirth, Elisabeth Kliemank, Gernot Poschet, Marc Freichel, Thomas Fleming, Alba Sulaj, Peter Paul Nawroth, Julia Szendroedi, Aurelio A. Teleman

**Affiliations:** 1 German Cancer Research Center (DKFZ) Heidelberg, Division B140, Heidelberg, Germany; 2 Faculty of Medicine, Heidelberg University, Heidelberg, Germany; 3 Faculty of Biosciences, Heidelberg University, Heidelberg, Germany; 4 Department for Endocrinology, Diabetology, Metabolic diseases and Clinical Chemistry, University Hospital Heidelberg, Im Neuenheimer Feld 410, Heidelberg, Germany; 5 German Center for Diabetes Research (DZD), Helmholtz Center Munich, Neuherberg, Germany; 6 Joint Heidelberg-IDC Translational Diabetes Program, Internal Medicine I, Heidelberg University Hospital, Heidelberg, Germany; 7 Department of Internal Medicine I and Clinical Chemistry, University Hospital, Heidelberg, Germany; 8 Centre for Organismal Studies (COS), Metabolomics Core Technology Platform, Heidelberg University, Im Neuenheimer Feld 360, Heidelberg, Germany; 9 Institute of Pharmacology, Heidelberg University, Heidelberg, Germany; 10 DZHK (German Centre for Cardiovascular Research), partner site Heidelberg/Mannheim, Heidelberg, Germany

**Keywords:** Post-translational modifications, Proteins, Enzymes, Metabolism

## Abstract

Pyruvate dehydrogenase (PDH) complex is a key regulator of metabolism, controlling flux from glycolysis into the tricarboxylic acid (TCA) cycle. PDH is often dysregulated in metabolic diseases and cancer. We discover here that in addition to its well characterized regulation by phosphorylation, PDH activity is also regulated via inhibitory oxidation of three cysteines of the DLAT subunit - Cys488, Cys586 and Cys618. Oxidation of these cysteines increases under oxidative stress, and is highly prevalent in mouse and human tissues. This mechanism enables cells to efficiently fight oxidative stress. Under oxidative stress, cells execute a metabolic shift, blocking glycolytic flux into the TCA cycle to shunt glycolytic carbons toward the pentose phosphate pathway. This generates NADPH which cells use to detoxify H_2_O_2_ and to reduce oxidized cysteines. We find that DLAT oxidation is required for cells to mount an effective antioxidant response. Mutations preventing DLAT oxidation render PDH resistant to oxidative inhibition, impair NADPH generation, reduce peroxide detoxification, and sensitize cells to oxidative damage and death. DLAT oxidation also drives a Warburg-like metabolic phenotype of increased glucose uptake, elevated lactate secretion, and reduced TCA cycle activity. Thus DLAT oxidation is a redox-sensing mechanism that regulates PDH activity and central carbon metabolism.

## Abstract (web summary)

Garcia-Cortizo et al. discover that the metabolic enzyme Pyruvate Dehydrogenase (PDH), which acts as a gatekeeper for metabolic flux from glycolysis into the TCA cycle, is inhibited by oxidation on three cysteines of the PDH subunit DLAT.

## Introduction

The pyruvate dehydrogenase (PDH) complex occupies a pivotal position at the junction between glycolysis and the tricarboxylic acid (TCA) cycle by converting pyruvate into acetyl-CoA^1,2^. PDH thereby acts as a gatekeeper that regulates metabolic flux from glycolysis into mitochondrial respiration, a function that is critical under both physiological and pathophysiological conditions^3,4^. For instance, PDH is central to metabolic flexibility - the ability of cells to switch from oxidizing glucose to oxidizing alternative fuel sources when glucose is limited. In low-glucose conditions, PDH activity decreases, reducing glycolytic flux into the TCA cycle, thereby causing cells to rely more heavily on alternative substrates, such as fatty acids and amino acids, to sustain TCA activity and energy production^5,6^. Metabolic inflexibility is a hallmark of type 2 diabetes in both humans and murine models^7–9^ and has been linked to impaired PDH regulation^7,10,11^.

In addition to controlling the flux of carbons from glycolysis into the TCA cycle, PDH also regulates, indirectly, the metabolic flux into other pathways that branch off of glycolysis, such as the pentose phosphate pathway (PPP) or lactate production. When PDH activity declines, glycolytic intermediates are increasingly redirected toward the PPP and into lactate generation^12,13^. Indeed, cells constantly adjust the partitioning of glycolytic flux between the TCA cycle and the PPP. The PPP provides the majority of cellular NADPH, which is essential for lipid synthesis and for fighting oxidative stress^14,15^. Accordingly, when cells experience oxidative stress, or when they are proliferating and require increased membrane biosynthesis, they reduce flux into the TCA cycle and divert more carbon into the PPP to boost NADPH production. This metabolic reprogramming, characteristic of the Warburg effect, is commonly observed in cancer cells, which face both elevated oxidative stress and high lipid biosynthetic demands^16–18^. One mechanism by which cells sense oxidative stress and reroute glycolytic flux involves pyruvate kinase, the glycolytic enzyme immediately upstream of PDH. Pyruvate kinase is inhibited by oxidation, creating a homeostatic feedback loop in which increased oxidative stress suppresses pyruvate kinase activity, thereby enhancing PPP flux and NADPH generation to counteract oxidative damage^15,19,20^. In principle, because PDH resides at the junction between glycolysis and the TCA cycle, it would be well-positioned to sense oxidative stress to modulate glycolytic flux into the TCA cycle versus the PPP. Whether oxidative stress directly regulates the PDH complex, however, remains poorly understood.

The PDH complex is one of the largest protein complexes known to date, with a molecular weight of 9 megadaltons^1,21^. Although the exact composition of the eukaryotic complex is still debated, it is thought to have a core composed of 60 copies of the E2 subunit DLAT plus 12 copies of E3BP. Attached to this central core are 20-30 subunits of the E1 enzyme, composed of two PDHA and two PDHB proteins, and 6-12 subunits of E3 composed of the DLD protein^1,22^. Activity of the PDH complex is primarily known to be regulated via inhibitory phosphorylation on three sites of PDHA: Ser 232, Ser293 and Ser300^1,23,24^. These sites are phosphorylated by four different pyruvate dehydrogenase kinases (PDKs) and dephosphorylated by two different pyruvate dehydrogenase phosphatases (PDP). In addition, phosphorylation of Tyr301^25^ and of Tyr289 by Src kinase^26^ also inhibits PDH activity. Recently, PDH was found to be inhibited by lactylation of PDHA1 on Lys336 in response to hypoxia^27^. Furthermore, in macrophages undergoing M1 polarization, DLD is modified by HNO on Cys477 and Cys484 in a DLAT-dependent and irreversible manner, thereby inhibiting DLD homodimer formation and activity^28^. Whether PDH activity is directly regulated by oxidative stress is not well understood.

Here, we study the effect of oxidative stress on PDH activity. Oxidative stress occurs when levels of reactive oxygen species (ROS) such as superoxide (O_2_•^-^) or hydrogen peroxide (H₂O₂) increase. This can occur in multiple different circumstances, such as (1) mitochondrial dysfunction or hypoxia, both of which cause impaired electron transport chain function and increased electron leakage to molecular oxygen, producing superoxide, (2) exposure to toxins, (3) exposure to radiation, (4) hyperactive oncogenic signaling, or (5) impaired antioxidant systems^29–31^. One situation where antioxidant systems are impaired is cells with elevated methylglyoxal (MG). MG is a metabolite that is produced non-enzymatically via the spontaneous dephosphorylation of the glycolytic intermediates dihydroxyacetone phosphate (DHAP) and glyceraldehyde-3-phosphate (GAP)^32^. As an α-oxoaldehyde, MG is highly reactive, interacting with macromolecules such as DNA and protein to form both reversible adducts^33^ and irreversible adducts referred to as advanced glycation end products (AGEs), which damage DNA and disrupt protein function^34^. MG levels are elevated in individuals with diabetes^35,36^, and MG is thought to mediate part of the tissue damage, complications, and pain observed in the disease^35,37,38^. In addition to these effects, elevated MG also causes oxidative stress^39–42^. This is due in part to increased ROS production^43^ and in part to decreased activity of key antioxidant enzymes^44^. Because the detoxification of both ROS and MG relies on reduced glutathione (GSH), elevated MG also impairs ROS detoxification by depleting reduced GSH^41^. Thus, we selected two model systems to study the effects of oxidative stress on PDH activity - treatment of cells with H_2_O_2_, and elevation of MG.

## Results

### High MG inhibits PDH activity

We first aimed to establish a cell culture model whereby MG levels are mildly, but chronically elevated, consistent with what is observed in individuals with diabetes^36,45^. To achieve this, we knocked out Glyoxalase 1 (GLO1), one of the enzymes capable of detoxifying MG^42,46,47^, in HEK293T cells using CRISPR-Cas9. This yielded a GLO1 knockout line (GLO1^KO^) with no detectable GLO1 protein or activity (Supplementary Fig. 1a–c). Although GLO1^KO^ cells do not exhibit elevated levels of free MG (Supplementary Fig. 1d), they do show mildly elevated levels of MG adducts such as MG-H1 and CEL^48^ on some mitochondrial proteins, but not cytosolic proteins (Suppl. Figure 1e). This indicates that excess MG is primarily sequestered by on proteins^49^, consistent with observations in other systems^48,50,51^. As expected, a flow cytometry assay using DCFDA^52^, confirmed that GLO1^KO^ cells have elevated ROS levels compared to GLO1^WT^ cells (Supplementary Fig. 1f). From this, we conclude that GLO1^KO^ cells are a suitable model for studying the consequences of mildly elevated MG and ROS.

**Fig. 1:**
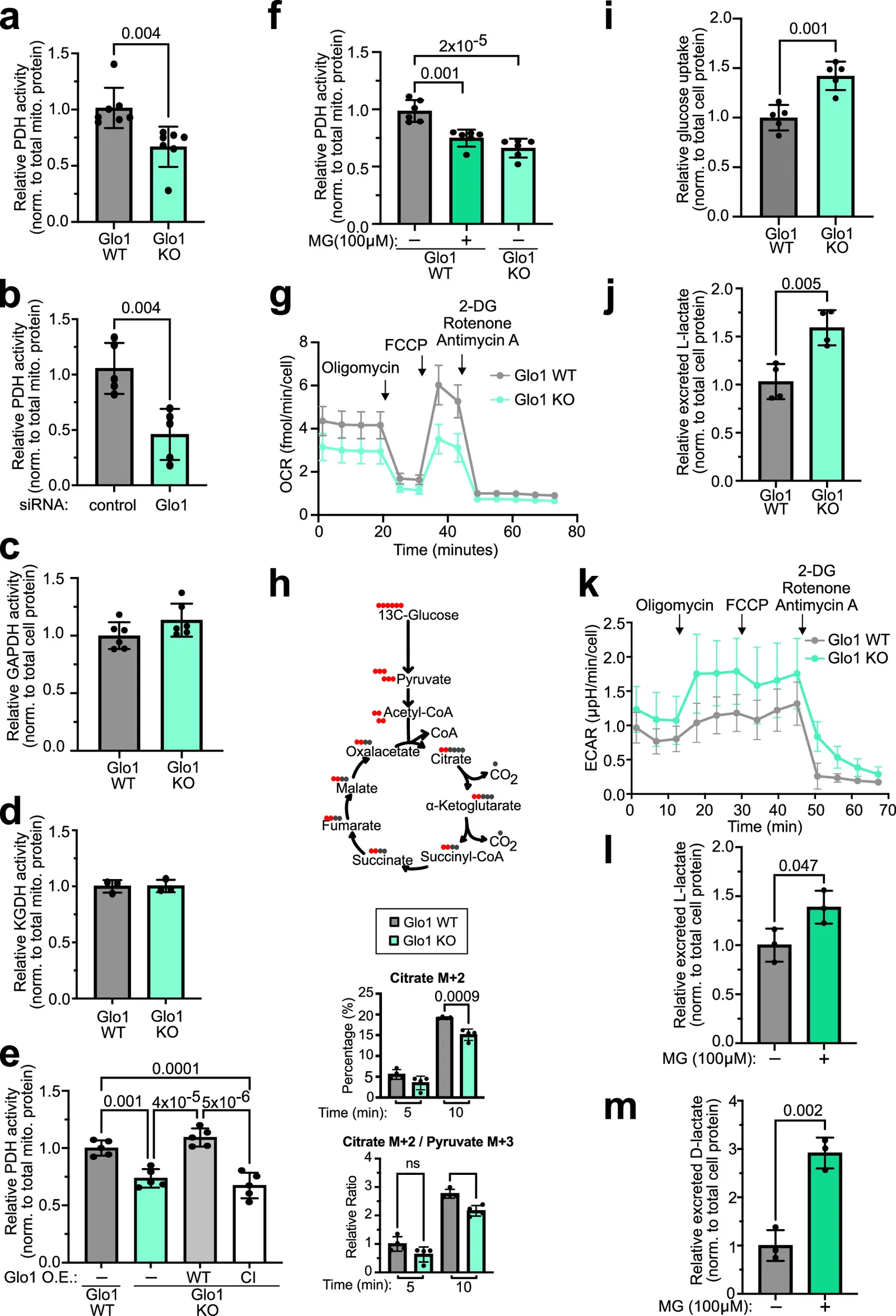
Methylglyoxal inhibits PDH activity. **a** PDH activity is reduced in GLO1 knockout (KO) HEK293T cells. *n* = 7 biological replicates, significance by two-tailed unpaired *t* test. **b** PDH activity is reduced in GLO1 knockdown HEK293T cells. siRNA targeting Renilla luciferase was used as a negative control. *n* = 5 biological replicates, significance by two-tailed unpaired *t* test. **c**, **d** Activity of other dehydrogenases, such as GAPDH (**c**) or KGDH (**d**), is not reduced in GLO1^KO^ HEK293T cells. *n* = 2 (**c**) or 3 (**d**) biological replicates, significance by two-tailed unpaired *t* test. **e** PDH activity is rescued by expression of WT GLO1, but not a catalytically inactive (CI) mutant of GLO1 in GLO1^KO^ HEK293T. *n* = 5 biological replicates, significance by one way ANOVA corrected for multiple comparisons with Tukey test. **f** PDH activity is inhibited in HEK293T cells incubated with 100 µM methylglyoxal (MG) for 1 h. *n* = 6 biological replicates, significance by one way ANOVA corrected for multiple comparisons with Tukey test. **g** GLO1^KO^ HEK293T cells have reduced oxygen consumption rates, measured via a SeaHorse Mito Stress Test Kit. Oxygen consumption is normalized per cell. *n* = 3 biological replicates on different days with multiple wells each. **h** GLO1^KO^ HEK293T cells have reduced carbon flux from glycolysis into the TCA cycle. Shown is the percent labeling with 13 C carbon into citrate after incubation for 5 or 10 min with 13 C glucose, as well as the ratio of labeled citrated to labeled pyruvate. *n* = 4 biological replicates, significance by one way ANOVA corrected for multiple comparisons with Šídák test. **i** GLO1^KO^ HEK293T cells have high glucose uptake, measured as the drop in glucose in the medium over a 24 h period, normalized to cellular protein. *n* = 2 biological replicates, significance by two-tailed unpaired *t* test. **j, k** GLO1^KO^ HEK293T cells produce more L-lactate, measured either as the increase in lactate in medium over a 24 h period (**j**) or using a SeaHorse assay (**k**). For (**k**), the same drugs were used as in panel (**g**) because a protocol that allows simultaneous ECAR and OCR measurements is used. *n* = 4 (**j**) or 3 (**k**) biological replicates on different days with multiple wells each. Significance by a two-tailed unpaired *t* test. **l**, **m** HEK293T cells incubated with 100 µM MG for 1 hour produced more L-lactate (**k**) and D-lactate (**m**). *n* = 3 biological replicates, significance by two-tailed unpaired *t* test. All data are presented as mean values +/− Std. Dev. Source data are provided as a Source Data file.

Next, we tested the activity of pyruvate dehydrogenase (PDH). This revealed that GLO1^KO^ cells have reduced PDH activity (Fig. 1a). All PDH activity measurements were done on purified mitochondrial, and normalized to total protein in the prep. We confirmed this phenotype using a transient siRNA-mediated knockdown of GLO1 to exclude clonal effects that can result from monoclonal selection of CRISPR-ized lines (Fig. 1b). In contrast, activity of other dehydrogenases, such as GAPDH or the mitochondrial α-ketoglutarate dehydrogenase (KGDH) are not reduced in GLO1^KO^ cells (Fig. 1c, d), indicating that the effect is specific for PDH. Interestingly, although KGDH and PDH share the same E3 subunit, dihydrolipoamide dehydrogenase (DLD), and both use lipoic acid as a cofactor, the effect appears specific to PDH, suggesting that the mechanism is likely independent of DLD or lipoic acid.

GLO1 could either affect PDH activity through its canonical MG-detoxifying catalytic activity, or through an alternative moonlighting function, possibly involving interactions with PDH or other protein complexes, as observed for other metabolic enzymes^53^. To distinguish these two options, we tested whether a catalytically inactive mutant (CI) of GLO1 carrying the E172Q mutation^54^ can rescue PDH activity. As expected, stable transfection of FLAG-tagged, wildtype GLO1 in GLO1^KO^ cells (Supplementary Fig. 1g) restored GLO1 activity (Supplementary Fig. 1h) and PDH activity (Fig. 1e), confirming that the reduced PDH activity observed in GLO1^KO^ cells is an on-target effect. In contrast, expression of the catalytically inactive mutant (Supplementary Fig. 1g, h) did not rescue PDH activity (Fig. 1e), indicating that the reduction in PDH activity in GLO1^KO^ cells is due to impaired MG detoxification. To confirm this, we added MG to GLO1^WT^ cells. Although at high concentrations MG is toxic, when added at lower concentrations it is rapidly detoxified by cells, with MG levels in the medium returning to near baseline levels within 30 minutes (Supplementary Fig. 2a), resulting in an increase in intracellular MG that is transient (Supplementary Fig. 2b). By titrating MG levels, we found that 100 µM MG causes an accumulation of mitochondrial MG adducts similar to those observed in GLO1^KO^ cells (Supplementary Fig. 2c). Like GLO1 knockout, treatment of cells with MG causes oxidative stress: addition of 100 µM MG to cells increases ROS levels (Supplementary Fig. 2d) and increased hyperoxidation of PRDX3 (Supplementary Fig. 2e, f), a readout for mitochondrial oxidative stress^55^. We therefore used 100 µM MG for all subsequent experiments. Incubation of GLO1^WT^ cells with 100 µM MG for 1 h recapitulated the GLO1 loss-of-function phenotype, leading to reduced PDH activity (Fig. 1f). In sum, mildly elevated MG leads to reduced activity of PDH.

### High MG re-routes glycolytic flux from the TCA cycle to lactate production

Reduced PDH activity should lead to several metabolic consequences: First, glycolytic flux into the TCA cycle should decrease. As a consequence, cells should upregulate glycolysis and L-lactate production to compensate for the reduced ATP production from respiration^56^. Indeed, a Seahorse respiration assay confirmed that oxygen consumption is reduced in Glo^KO^ cells (Fig. 1g). To directly assess flux into the TCA cycle, we performed metabolomic flux analysis on GLO1^WT^ or GLO1^KO^ cells incubated with 13C-labeled glucose for 5 or 10 min (Supplementary Data 1). As expected, the ratio of labeled citrate to labeled pyruvate, a readout for PDH activity, was reduced in GLO1^KO^ cells (Fig. 1h), as was labeling of succinate further downstream (Supplementary Fig. 3a).

As expected, this led to increased glucose uptake in Glo1^KO^ cells (Fig. 1i) as well as increased L-lactate production in both GLO1^KO^ cells (Fig. 1j, k) and in cells treated with 100 µM MG for 1 hour (Fig. 1l). Since MG detoxification via the sequential action of GLO1 and GLO2 converts MG into D-lactate^46^, which is distinct from its stereoisomer L-lactate generated by glycolysis, D-lactate production also increased upon MG treatment (Fig. 1m). These results are consistent with previous studies showing that 3T3-L1 adipocytes and HepG2 cells increase glucose uptake and L-lactate production when exposed to MG^39,57^.

We observed similar metabolic phenotypes in HeLa cells: GLO1^KO^ HeLa cells (which we characterized previously^58^) and GLO1 knockdown HeLa cells also exhibited reduced PDH activity (Supplementary Fig. 3b, c), increased glucose uptake (Supplementary Fig. 3d), and increased L-lactate production (Supplementary Fig. 3e). In sum, elevated MG induces a Warburg-like metabolic phenotype in different cell types, with reduced PDH activity, increased glucose uptake, increased lactate production, and reduced TCA cycle activity.

### Regulation of PDH by MG is not mediated by changes in phosphorylation

**Fig. 2:**
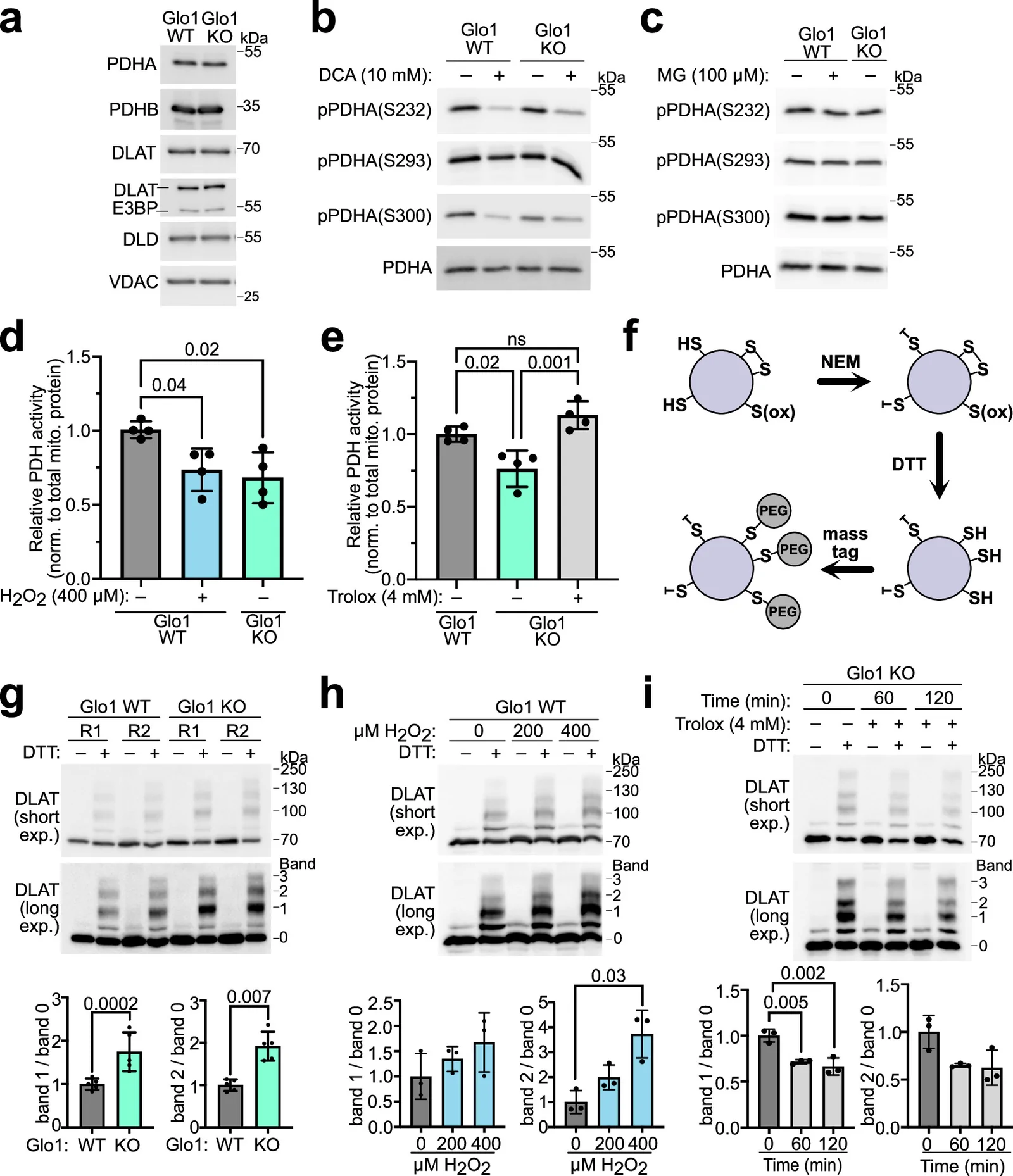
PDH activity is regulated by oxidation. **a** Levels of PDH subunits do not decrease in GLO1^KO^ HEK293T cells. Representative of 2 biological replicates. **b** PDH phosphorylation is not increased in GLO1^KO^ HEK293T cells. Representative of 4 biological replicates. The PDK inhibitor DCA is used as a positive control to reduce PDHA phosphorylation. **c** PDH phosphorylation is not increased in HEK293T cells incubated with methylglyoxal (MG) (100 µM, 1 h). Representative of 2 biological replicates. **d** PDH activity is inhibited in HEK293T cells incubated with 400 µM H_2_O_2_ for 1 h. *n* = 4 biological replicates, significance by one way ANOVA corrected for multiple comparisons with Tukey test. **e** The antioxidant Trolox (4 mM, 1 h) rescues the decreased PDH activity observed in GLO1^KO^ HEK293T cells. *n* = 4 biological replicates, significance by one way ANOVA corrected for multiple comparisons with Tukey test. **f** Schematic diagram of the PEG-exchange assay whereby reduced cysteines are blocked with N-ethylmaleimide (NEM), oxidized cysteines are reduced with DTT, and then the free cysteines that are generated by the DTT are coupled to a mass-tag visible as a mobility shift on an SDS-PAGE gel. **g** The DLAT subunit of PDH is more oxidized in GLO1^KO^ HEK293T cells compared to GLO1 WT cells. *n* = 5 biological replicates, including replicates from Supplementary Fig. 6b. Significance by two-tailed unpaired *t* test. **h** Oxidation of DLAT increases in HEK293T cells treated for 1 h with 200 µM or 400 µM H_2_O_2_. Representative blot (top) of 2 biological replicates quantified below. Significance by one way ANOVA corrected for multiple comparisons with the Tukey test. **i** Oxidation of DLAT in GLO1^KO^ HEK293T cells is reduced by the antioxidant trolox (4 mM for 1 or 2 h). Representative blot (top) of 3 biological replicates, quantified below. Significance by one way ANOVA corrected for multiple comparisons with Šídák test. All data are presented as mean values +/− Std. Dev. Source data are provided as a Source Data file.

To understand the mechanism by which MG affects PDH activity, we first investigated whether the protein levels of PDH subunits are reduced in GLO1^KO^ cells, but this was not the case (Fig. 2a). PDH activity is regulated via inhibitory phosphorylation on Ser232, Ser293 and Ser300 of its PDHA subunit^59^. To verify the sensitivity of the phospho-antibodies, we treated cells with dichloroacetate (DCA), an inhibitor of the Pyruvate Dehydrogenase Kinases (PDKs), which phosphorylate these sites^60^, and observed the expected drop in PDHA phosphorylation (Fig. 2b). However, we did not observe an increase in phosphorylation of these sites in GLO1^KO^ cells (Fig. 2b) or in MG treated GLO1^WT^ cells (Fig. 2c). Thus, the reduction in PDH activity in GLO1^KO^ cells cannot be explained by increased inhibitory phosphorylation. Supporting this, although DCA increases PDH activity, it does not eliminate the difference in PDH activity between GLO1^WT^ and GLO1^KO^ cells (Supplementary Fig. 4a), indicating that MG does not act via the PDKs. In sum, MG and phosphorylation regulate PDH via distinct mechanisms.

### Lack of evidence for a direct effect of MG on PDH via MG-adducts

MG can form adducts on lysine and arginine residues of proteins, leading to protein dysfunction. The most common adduct is hydroimidazolone (MG-H1)^48^. To test if GLO1^KO^ cells have more MG-H1 modifications on PDH subunits than GLO1^WT^ cells, we immunoprecipitated PDH and immunoblotted with anti-MG-H1 antibody. Although immunoprecipitation of FLAG-tagged PDHA leads to co-immunoprecipitation of all other subunits of PDH (Supplementary Fig. 4b), we also immunoprecipitated FLAG-PDHB and HA-DLAT to enrich for them. None of the PDH subunits, however, was differentially modified with MG-H1 in GLO1^KO^ compared to GLO1^WT^ cells (Supplementary Fig. 4c). Since MG can form multiple different types of adducts on proteins, we also immunoprecipitated the PDH complex via FLAG-PDHA from GLO1^WT^ and GLO1^KO^ cells and analyzed the proteins by mass spectrometry. This revealed only one modification that differed significantly between the two samples – a CEL modification on Lys313 of PDHA, which was elevated in GLO1^KO^ cells. However, we were able to validate this result by immunoblotting in only one replicate (Supplementary Fig. 4d) out of three attempts, indicating that the difference in this modification between GLO1^WT^ and GLO1^KO^ cells is not very robust. In sum, we were unable to find convincing evidence to support the hypothesis that MG regulates PDH activity by directly forming adducts on PDH.

### Inhibition of PDH activity is mediated by oxidation

Since MG causes increased ROS^39–42^ Supplementary Fig. 1f, Supplementary Fig. 2d–f), and ROS can oxidize cysteines to affect protein function^61,62^, we asked if MG regulates PDH activity via ROS. To first test if ROS can regulate PDH activity, we treated HEK293T cells with 400 µM H_2_O_2_ for 1 h and found that this inhibits PDH activity to levels similar to those observed in GLO1^KO^ cells (Fig. 2d and Supplementary Fig. 5a, b). This effect is reversible (Supplementary Fig. 5c) and can be recapitulated by treating purified PDH with H_2_O_2_ in vitro (Supplementary Fig. 5d)^63^, indicating it is direct and independent of phosphorylation. In contrast, treatment of purified PDH with MG in vitro had no effect on PDH activity (Supplementary Fig. 5e). To assess whether endogenously produced H_2_O_2_ can also inhibit PDH activity, we treated cells with 3-amino-1,2,4-triazole (3AT), an inhibitor of catalase, one of the main enzymes detoxifying H_2_O_2_^64^. This also resulted in reduced PDH activity (Suppl. Figure 5f), indicating that endogenous H_2_O_2_ can inhibit PDH. Notably, H_2_O_2_ is primarily produced in mitochondria^65^, where PDH is also localized.

Since treatment of cells with H_2_O_2_ causes a drop in PDH activity (Fig. 2d), one would expect that H_2_O_2_ also causes a drop in mitochondrial respiration. However, it has been reported that treatment of cells with H_2_O_2_ causes an increase in mitochondrial respiration^66^. Mitochondria can use several fuel sources to drive respiration, including glucose via glycolysis, glutamine via glutaminolysis, and fatty acids via beta-oxidation^67^, raising the possibility that the increased respiration is due to a shift in fuel usage. Consistent with previous reports, we confirmed that H_2_O_2_ causes increased respiration (Supplementary Fig. 5g). We then tested if this is due to increased usage of glucose, glutamine or fatty acids by pharmacologically inhibiting the respective pathways. This revealed that the increased respiration in response to H_2_O_2_ is due to increased glutaminolysis, because it is abolished upon treatment with BPTS (Bis-2-(5-phenylacetamido-1,3,4-thiadiazol-2-yl) ethyl sulfide), a specific inhibitor of glutaminase (Supplementary Fig. 5g). Indeed, in the presence of BPTS, H_2_O_2_ causes a decrease in respiration, as one would expect from the drop in PDH activity.

Having established that ROS can inhibit PDH, we next asked whether the reduced PDH activity observed in GLO1^KO^ cells is due to elevated oxidation. To test this, we examined if the antioxidant Trolox can revert the phenotype. Indeed, treatment of Glo1^KO^ cells with Trolox restored PDH activity to wildtype levels (Fig. 2e). Together, these findings indicate that MG suppresses PDH activity through an oxidation-dependent mechanism.

### DLAT is oxidized on three cysteines

To detect protein oxidation, we adapted a method based on the acyl-PEG exchange (APE) assay^68^. In this modified method (Fig. 2f), we first lyse cells and block free cysteine thiols with N-methylmaleimide (NEM). Next, we treat the lysate with DTT to reduce and remove oxidative modifications on cysteines, such as glutathionylation or disulfide bonds. These newly-generated free thiol groups are then labeled by reacting with methoxy-polyethylene glycol-maleimide (mPEG), which covalently attaches a large PEG group to the cysteines. This causes a mobility shift of the protein during SDS-PAGE, which can be detected by immunoblotting, with each modified cysteine resulting in an additional band. Each sample is split in two and either treated with DTT, or without DTT as a negative control. We applied this method to investigate each subunit of the PDH complex. This showed that PDHA and PDHB are not oxidized, as no additional bands appeared after DTT treatment (Supplementary Fig. 6a). In contrast, E3BP and DLD showed oxidation at one cysteine, seen by the appearance of a single additional band in the DTT-treated samples (Supplementary Fig. 6a). However, the extent of oxidation of E3BP and DLD, as determined by the fraction of the protein that shifts in the presence of DTT, is not different in GLO1^WT^ versus GLO1^KO^ cells. In contrast, for the DLAT subunit, we observed three up-shifted bands (Fig. 2g), indicating the presence of oxidation on three separate cysteines in GLO1^WT^ cells. Furthermore, the extent of oxidation was higher in GLO1^KO^ cells compared to GLO1^WT^ cells (quantified in the lower panels, Fig. 2g), as well as in cells treated with MG (Supplementary Fig. 6b), suggesting that oxidation of DLAT may be responsible for the reduced PDH activity in GLO1^KO^ cells. Indeed, oxidation of DLAT consistently negatively correlated with PDH activity: DLAT oxidation increased when GLO1^WT^ cells were treated with H_2_O_2_ (Fig. 2h), which causes reduced PDH activity (Fig. 2d). Similarly, DLAT oxidation increased when cells were treated with 3AT (Supplementary Fig. 6c), indicating that this oxidation is caused by endogenous H_2_O_2_. Finally, Trolox reduced DLAT oxidation in GLO1^KO^ cells (Fig. 2i), correlating with the rescue in PDH activity (Fig. 2e).

To definitively prove that oxidation of DLAT is responsible for the drop in PDH activity under oxidative stress, we next aimed to identify the specific cysteine residues in DLAT that are oxidized.

### DLAT is oxidized on Cys488, Cys586 and Cys618

**Fig. 3:**
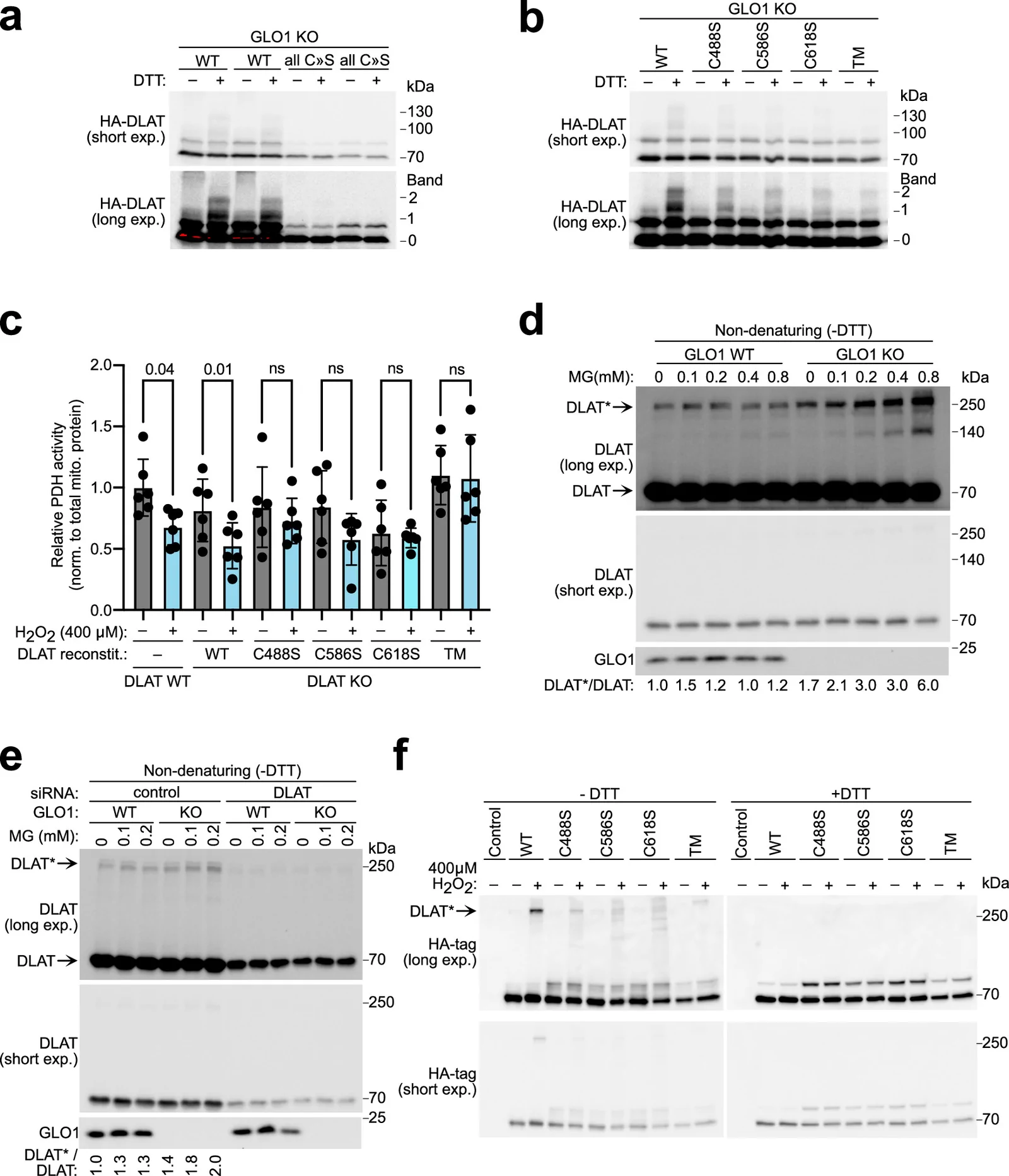
DLAT oxidation on Cys488, Cys586 or Cys618 inhibits its activity. **a** Oxidation of DLAT is abolish by mutating all cysteines in DLAT to serine. Representative of 2 biological replicates. **b** Oxidation of DLAT is reduced in the single point mutants for Cys488, Cys586 or Cys618, and more strongly reduced in the triple mutant (TM). Representative of 3 biological replicates. **c** Activity of PDH in HEK293T DLAT knockout cells expressing a triple mutant (TM) DLAT (C488S, C586S, C618S) does not decrease upon treatment with H_2_O_2_ (400 µM, 1 h), and the single point mutants are partially resistant. *n* = 6 biological replicates. All columns are normalized to the first (control condition without H_2_O_2_ treatment). Significance by one-way ANOVA with Geisser–Greenhouse correction, followed by Šídák-adjusted post hoc tests. **d** DLAT forms high molecular weight aggregates, which are increased in GLO1^KO^ HEK293T cells or in response to treatment with methylglyoxal for 1 h. Representative of 2 biological replicates. **e** The higher molecular weight band detected by the DLAT antibody is specific for DLAT, because it decreases in intensity upon DLAT knockdown. siRNA targeting Renilla luciferase was used as a negative control. Representative of 2 biological replicates. **f** Cys488, Cys586 and Cys618 of DLAT are involved in the formation of higher molecular weight aggregates. Incubation of HEK293T cells with 400 µM H_2_O_2_ for 1 h induces formation of the high molecular weight DLAT band (DLAT*) in cells transfected with HA-DLAT(WT) but not HA-DLAT with the indicated point mutation or triple mutation (TM). Representative of 2 biological replicates. All data are presented as mean values +/− Std. Dev. Source data are provided as a Source Data file.

To identify the cysteines in DLAT that are oxidized, we first confirmed that mutating all cysteines in DLAT to serine abolishes the up-shift in our PEG exchange assay (Fig. 3a). There is one band located between the bands we refer to as ‘band 0’ and ‘band 1’ which sometimes changes in response to DTT treatment (Fig. 2g–i) and sometimes does not (Fig. 3a). This band is not affected by mutation of all cysteines, and since its behavior is inconsistent between experiments, we chose to disregard it here, though it may be relevant for future studies.

Next, we aimed to identify which cysteines are modified. We individually mutated seven of the nine cysteines in DLAT to serine and found that mutation of Cys488, Cys586 and Cys618 reduced DLAT oxidation (Supplementary Fig.6d), as did the triple mutation (TM) of these three residues (Fig. 3b). In sum, DLAT Cys488, Cys586 and Cys618 are targets of oxidation.

### Oxidation of DLAT Cys488, Cys586 and Cys618 regulates PDH activity

The results presented above raise the hypothesis that oxidative stress causes oxidation of DLAT on Cys488, Cys586 and Cys618, which in turn inhibits PDH activity. We therefore tested whether these three cysteines mediate the effect of oxidative stress on PDH activity. To this end, we generated a DLAT knockout cell line and reconstituted it with wild-type (WT) DLAT, with C488S, C586S, or C618S single mutant DLAT, or with triple-mutant (TM) DLAT. The DLAT knockout cell line, which we generated using CRISPR/Cas9 (Supplementary Fig. 7a), entirely lacks DLAT protein (Supplementary Fig. 7b) and PDH activity (Supplementary Fig. 7c). This cell line acidifies the cell culture medium rapidly and dies if the medium is not exchanged frequently - an expected consequence of the cells compensating for the lack of PDH by up-regulating glycolysis and lactate production. We then stably transfected this line with wildtype or mutant DLAT (Supplementary Fig. 7b). Interestingly, triple mutant DLAT supports higher PDH activity than wildtype DLAT (Supplementary Fig. 7c, d) despite being a bit less stable than wildtype protein (Supplementary Fig. 7b). This is consistent with oxidation of these three cysteines being inhibitory. In order to control for batch effects, we generated a second set of cells reconstituted with either wild-type or triple mutant DLAT. Also in this second batch, cells reconstituted with triple-mutant DLAT have elevated PDH activity (#2, Supplementary Fig. 7d). Correspondingly, cells with TM DLAT also have elevated respiration (Supplementary Fig. 7e).

Next, we treated these cell lines with H_2_O_2_ and found that the single mutants provided partial protection of PDH activity against oxidation, while the triple mutant completely blunted the effect of oxidation on PDH activity (Fig. 3c). In sum, oxidation of DLAT at Cys488, Cys586 and Cys618 reduces PDH activity.

### DLAT oxidation acts independently of phosphorylation on PDH activity

Since PDH activity is regulated by phosphorylation and oxidation, we wondered how these two mechanisms integrate to regulate PDH activity. We first assessed the phosphorylation status of PDH in cells treated with H_2_O_2_, which increases PDH oxidation (Fig. 2h) and reduces PDH activity (Fig. 2d). In this case, H_2_O_2_ causes a substantial decrease in PDH phosphorylation (Suppl. Fig. 8a), which *per* se should increase PDH activity. However, the net effect is a drop in PDH activity, indicating that the oxidation inhibits PDH despite reduced inhibitory phosphorylation. The more moderate intervention with catalase inhibitor 3AT, which also causes increased PDH oxidation (Supplementary Fig. 6c) and reduced PDH activity (Supplementary Fig. 5f), did not cause a detectable change in PDH phosphorylation (Supplementary Fig. 8b), indicating that changes in PDH oxidation are sufficient to reduce its activity. Finally, when we treated cells with Trolox, which reduces PDH oxidation (Fig. 2i) and increases PDH activity (Fig. 2e), we observed a mild decrease in PDH phosphorylation (Suppl. Fig. 8c). In this case, both the reduction in oxidation and in phosphorylation contribute to the increase in PDH activity.

In sum, PDH activity seems to always negatively correlate with DLAT oxidation. In some cases, there is a discrepancy between PDH activity and its phosphorylation state, indicating that it is important to directly measure PDH activity under experimental conditions rather than relying solely on phosphorylation status as a proxy.

### DLAT oxidation involves a disulfide bond

The thiol group of cysteines is susceptible to a variety of post-translational modifications in response to oxidative stress, including S-nitrosylation, S-glutathionylation and disulfide bonding^61^. We next sought to better understand the nature of the oxidation occurring at Cys488, Cys586 and Cys618. The PEG exchange assay we used to detect oxidation at these cysteines entails that the oxidation is reversible by DTT, making it unlikely that the thiol groups of these cysteines are oxidized to sulfinic acid or sulfonic acid, since these modifications are not reversed by DTT. We ruled out sulfenic acid, because numerous mass spectrometry screens failed to detect sulfenic acid on these DLAT residues^69–71^. We therefore assessed whether DLAT undergoes S-nitrosylation. Immunoprecipitation of HA-DLAT and immunoblotting with an antibody that detects nitrosylated cysteine revealed no difference in the degree of S-nitrosylation between GLO1^WT^ and GLO1^KO^ cells (Supplementary Fig. 8d). (To validate the method, we used the NO donor DETA NONOate as a positive control, which confirmed the expected nitrosylation response). Interestingly, another subunit of the PDH complex, DLD, was reported to be nitrosylated in a manner that is not reversed by DTT^28^.

Under oxidative stress conditions, cysteines can be glutathionylated^72^. To detect this, we incubated GLO1^WT^ and GLO1^KO^ cells with biotinylated glutathione ethyl ester (BioGEE), a cell-permeable, biotinylated glutathione analog which allows for metabolic labeling and subsequent streptavidin mediated pull-down to detect glutathionylated proteins (Supplementary Fig.. 8e). As a positive control, we treated GLO1^WT^ cells with H_2_O_2_ and detected by immunoblotting the expected increase in GAPDH glutathionylation^73^. This revealed that DLAT also becomes glutathionylated in response to H_2_O_2_, however we found no difference in the level of DLAT glutathionylation between GLO1^WT^ and GLO1^KO^ cells. Glutathionylation of DLAT was previously observed in vivo when macrophages are stimulated with LPS and in vitro following GSH oxidation with diamide^74^, but in both cases the gluthationylation of DLAT occurred on lipoic acid, whereas in our context the oxidation occurs on cysteine residues.

Cysteines can form disulfide bonds in response to oxidative stress^61^. These bonds can be detected on an SDS-PAGE gel under non-reducing conditions, where intramolecular disulfide bonds cause proteins to migrate faster, whereas intermolecular disulfide bonds cause proteins to migrate slower^75^. Treatment of GLO1^WT^ and GLO1^KO^ cells with MG for 1 hour caused a concentration-dependent increase in the intensity of a 210 kDa band, corresponding to a potential trimer of DLAT, which we term DLAT* (Fig. 3d). This high molecular weight band is specific for DLAT, because DLAT knockdown causes a reduction in the intensity of both DLAT and DLAT* (Fig. 3e). Quantification of this band, normalized to total DLAT, showed that GLO1^KO^ cells have 70% more DLAT* than GLO1^WT^ cells (Fig. 3d). Interestingly, formation of this higher molecular weight species occurs in response to oxidative stress (Fig. 3f) and involves Cys488, Cys586 and Cys618, because DLAT* formation is strongly reduced in the single mutants and abolished in the triple mutant (Fig. 3f). These results strongly suggest that Cys488, Cys586 and Cys618 form intermolecular disulfide bonds in response to MG or oxidative stress. In addition, these residues may also be involved in others forms of oxidation which we have not tested.

Two mechanistic questions remain. First, the stoichiometry of this intermolecular disulfide bonding is relatively low, suggesting that only a subset of the 60 DLAT subunits in one PDH complex needs to be oxidized to inhibit PDH activity. How this works at the structural level remains to be explored. Second, if DLAT* is a DLAT trimer, the exact mechanism by which three cysteines interconnect three DLAT molecules remains to be elucidated. Answering these questions will require additional structural insights into this megadalton complex.

### Oxidation of DLAT forms a homeostatic feedback loop that promotes survival to oxidative stress

**Fig. 4:**
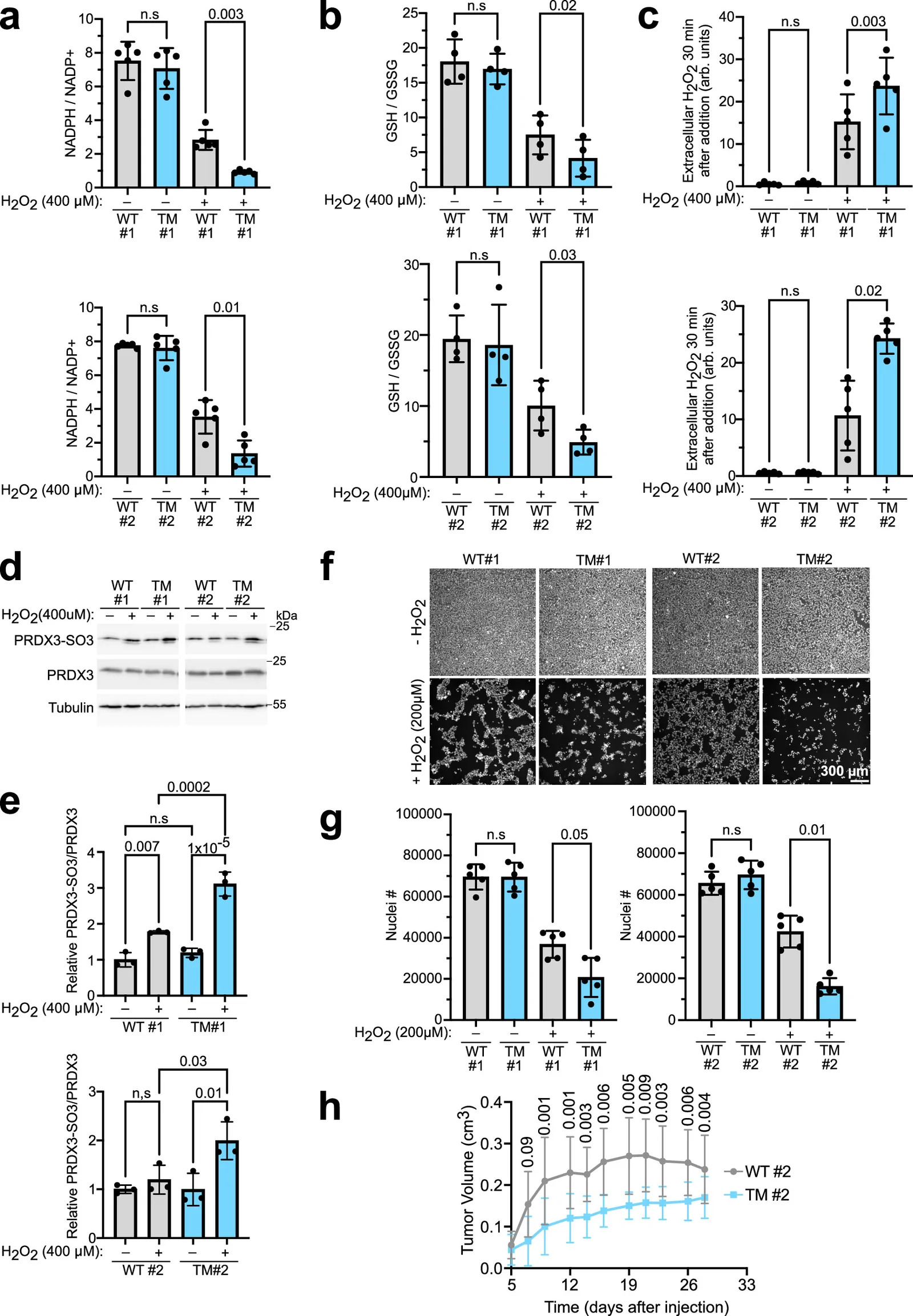
DLAT oxidation is part of a homeostatic feedback loop enabling cells to fight oxidative stress. **a** DLAT knockout cells reconstituted with C488S, C586S, C618S triple-mutant (TM) DLAT have lower NADPH levels upon treatment with H_2_O_2_ compared to cells reconstituted with wildtype DLAT. Cells were treated with 400 µM H_2_O_2_ for 1 h prior to measurement of NADPH and NADP + . *n* = 5 biological replicates. Significance by one-way ANOVA with Geisser–Greenhouse correction, followed by Šídák-adjusted post hoc tests. **b** DLAT knockout cells reconstituted with C488S, C586S, C618S triple-mutant (TM) DLAT have lower levels of reduced glutathione upon treatment with H_2_O_2_ compared to cells reconstituted with wildtype DLAT. Cells were treated with 400 µM H_2_O_2_ for 1 hour prior to measurement of reduced (GSH) and oxidized (GSSG) glutathione. *n *= 5 biological replicates. Significance by one-way ANOVA with Geisser–Greenhouse correction, followed by Šídák-adjusted post hoc tests. **c** DLAT knockout cells reconstituted with C488S, C586S, C618S triple-mutant (TM) DLAT detoxify H_2_O_2_ less well than cells reconstituted with wildtype DLAT. Cells were treated with 400 µM H_2_O_2_ for 30 min prior to measurement of H_2_O_2_ in the medium. *n* = 5 biological replicates. Significance by one-way ANOVA with Geisser–Greenhouse correction, followed by Šídák-adjusted post hoc tests. **d**, **e** DLAT knockout cells reconstituted with C488S, C586S, C618S triple-mutant (TM) DLAT have more hyperoxidation of PRDX3 upon treatment with H_2_O_2_ compared to cells reconstituted with wildtype DLAT. PRDX3 hyperoxidation is detected with an antibody that detects sulfinylated PRDX3 (SO₃-PRDX3). **d** Representative western blot. **e** Quantification of 3 biological replicates. Significance by one-way ANOVA with Geisser–Greenhouse correction, followed by Šídák-adjusted post hoc tests (**f**, **g**) DLAT knockout cells reconstituted with C488S, C586S, C618S triple-mutant (TM) DLAT die more than cells reconstituted with wildtype DLAT upon treatment with H_2_O_2_. (f) Representative images. **g** Quantification of 5 biological replicates. Significance by one-way ANOVA with Geisser–Greenhouse correction, followed by Šídák-adjusted post hoc tests (**h**) Xenografts of DLAT knockout HEK293T cells reconstituted with C488S, C586S, C618S triple-mutant (TM) DLAT grow more slowly than the equivalent xenografts of cells reconstituted with wildtype DLAT. *n* = 8 mice. Significance by multiple two-sided unpaired *T* test. All data are presented as mean values +/- Std. Dev. Source data are provided as a Source Data file.

The results presented above indicate that oxidative stress leads to oxidation of DLAT, thereby inhibiting PDH and reducing glycolytic flux into the TCA cycle. This should enable glycolytic flux to re-route towards the pentose phosphate pathway (PPP) to produce NADPH to fight the oxidative stress. To test if this is the case, we analyzed if cells containing triple mutant (TM) DLAT are impaired in fighting oxidative stress. The difference between cells with wildtype DLAT and cells with TM DLAT reflects the metabolic consequence of DLAT oxidation. Indeed, when challenged with H_2_O_2_, cells with TM DLAT have a much stronger drop in NADPH levels than cells with wildtype DLAT (Fig. 4a). Roughly half of the NADPH generated in oxidative stress conditions requires DLAT to become oxidized on these three cysteines (Fig. 4a). Correspondingly, the ratio of reduced to oxidized glutathione (GSH/GSSH) also drops more strongly upon H_2_O_2_ treatment in cells with TM DLAT (Fig. 4b). Cells use NADPH and GSH to detoxify H_2_O_2_. To test the detoxification capacity of cells, we treated them with 400 µM H_2_O_2_ and quantified H_2_O_2_ levels in the medium over time. In the absence of cells, H_2_O_2_ is relatively stable in medium within a 120 min timeframe (Supplementary Fig. 9a) whereas in the presence of cells it is detoxified very rapidly, dropping to roughly 20 µM within 30 minutes (Supplementary Fig. 9b). This detoxification was impaired in cells with TM DLAT (Fig. 4c). As a consequence, cells with TM DLAT suffer more oxidative damage to their proteins, such as hyperoxidation of PRDX3 (Fig. 4d, e), and die more readily in response to H_2_O_2_ (Fig. 4f, g). In sum, down-regulation of PDH activity via oxidation of DLAT Cys488, Cys586 and Cys618 is important for cells to sense and fight oxidative stress.

Cells in the core of a tumor are exposed to oxidative stress^76^ and previous work has shown that cells with impaired capacity to fight oxidative stress are also impaired in tumor formation^19^. To test this, we generated xenografts in mice with cells carrying either wildtype DLAT or TM DLAT. Although TM cells proliferate as quickly as WT cells in cell culture (Fig. 4g), the TM cells formed tumors more slowly than WT cells (Fig. 4h). Thus, the ability of cells to re-route metabolic flux via oxidation of DLAT is also relevant in tumors.

### Conservation in HepG2 cells

We then sought to validate whether the effect of oxidative stress on PDH is conserved in HepG2 cells, which are a hepatocellular carcinoma cancer model, and derive from liver, a key tissue involved in systemic glucose homeostasis^77^. As in other cell lines, MG increases ROS levels in HepG2 cells^39^. Consistent with our findings in HEK293T and HeLa cells, in HepG2 cells MG reduces PDH activity (Supplementary Fig. 10a), does not affect PDH phosphorylation (Supplementary Fig. 10b), and increases PDH oxidation (Supplementary Fig.10c). Incubation of HepG2 cells with H_2_O_2_ for 1 hour decreases PDH activity (Supplementary Fig. 10d), only mildly affected PDH phosphorylation (Supplementary Fig. 10e), and increased PDH oxidation (Supplementary Fig. 10f). Furthermore, both MG and H_2_O_2_ lead to an increase in the high molecular weight DLAT species (Supplementary Fig. 10g, h). In sum, the regulation of PDH by MG and oxidative stress appears to be conserved in liver cells.

### MG impairs PDH activity in vivo in mice

**Fig. 5:**
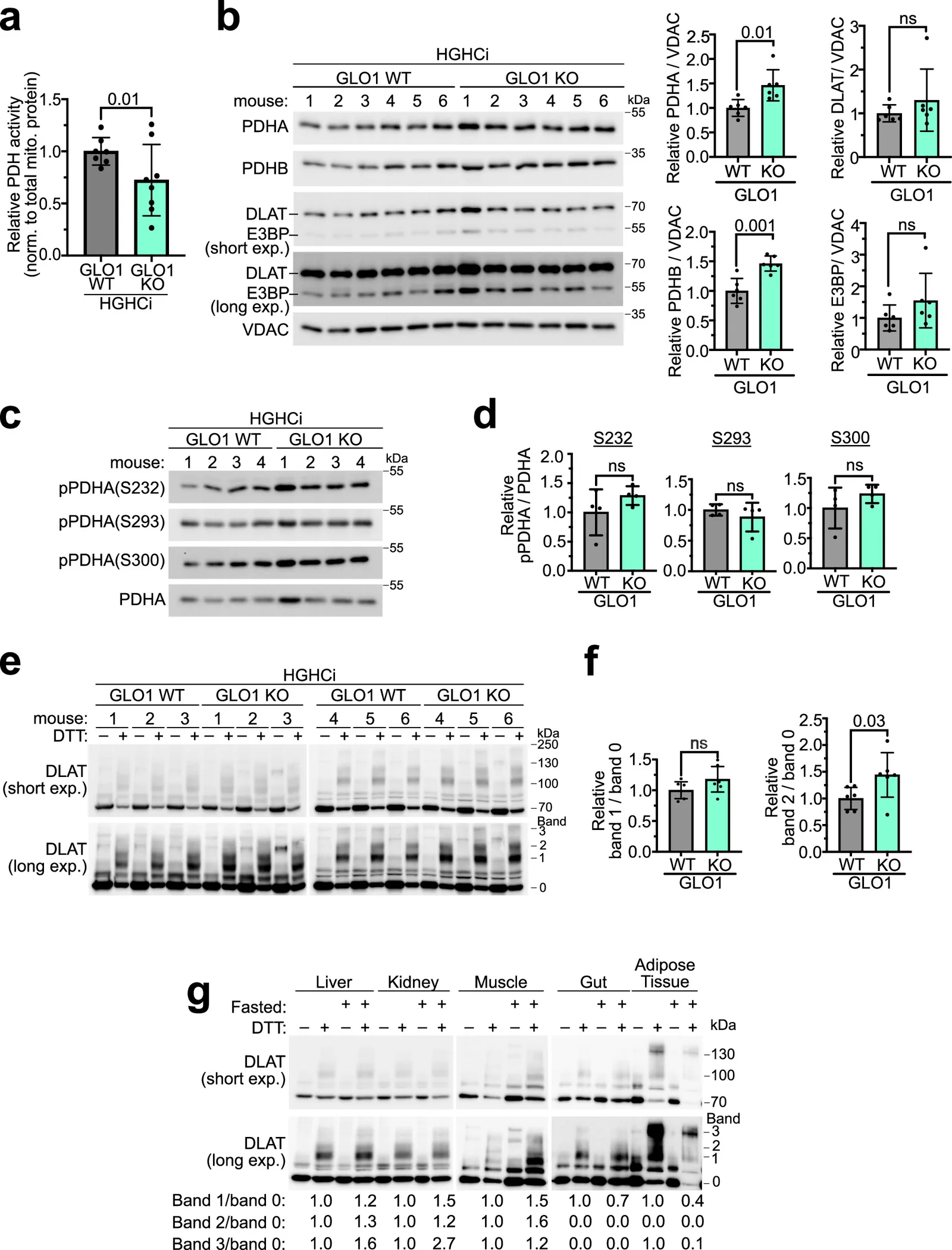
PDH oxidation is regulated in vivo in mice by methylglyoxal and by fasting. **a** PDH activity is reduced in livers of GLO1^KO^ mice with high glucose and high cholesterol (HGHCi). *n* = 7 WT mice and *n* = 8 for Glo1 KO mice. Significance by the two-tailed Kolmogorov-Smirnov test. **b** Expression of PDH subunits is not reduced in GLO1^KO^ HGHCi mice. *n* = 6 mice. Significance by a one-tailed unpaired *t* test. **c**, **d** PDH phosphorylation is not changed in the liver of GLO1^KO^ HGHCi mice. Quantified in (**d**). *n* = 4 mice, significance by two-tailed unpaired *t* test. **e**, **f** DLAT oxidation is higher in GLO1^KO^ HGHCi mice compared to GLO1 WT HGHCi mice, quantified in (**f**). In each case, the intensities of bands 1-3 were normalized to band 0 from the same + DTT sample. *n* = 6 mice, significance by two-tailed unpaired t test. **g** DLAT oxidation increases in several mouse tissues upon fasting. In each case, the intensities of bands 1-3 were normalized to band 0 from the same + DTT sample, and then normalized to the non-fasted sample for each tissue. *n* = 1 biological replicates x 2 conditions. All data are presented as mean values +/- Std. Dev. Source data are provided as a Source Data file.

We next investigated whether MG can inhibit liver PDH in vivo using a mouse model. To test this, we evaluated PDH activity in livers from GLO1^WT^ and GLO1^KO^ mice^47^ in which we induced hypercholesterolemia and hyperglycemia (HGHCi). These animals express a gain-of-function variant of murine PCSK9^78^ in the liver, and are fed a high-fat diet in order to induce hypercholesterolemia. In addition, the animals are injected with streptozotocin to induce hyperglycemia^78^. Consistent with our cell culture results, loss of GLO1 caused a significant decrease in liver PDH activity (Fig. 5a). This reduction in activity was not due to changes in PDH protein levels or phosphorylation because, if anything, PDHA and PDHB protein levels were mildly increased in GLO1^KO^ livers (Fig. 5b), and PDHA phosphorylation was not significantly changed (Fig. 5c, d). Instead, in line with our cell culture findings, DLAT oxidation was mildly but significantly increased in livers from GLO1^KO^ HGHCi mice (Fig. 5e, f). This finding demonstrates that also in vivo DLAT oxidation occurs at substantial levels, is elevated under conditions of glycation stress, and correlates with PDH activity.

### Fasting modulates DLAT oxidation

We wondered whether oxidation of DLAT can be modulated in vivo by physiological conditions. Under fasting conditions, PDH activity is typically inhibited to conserve pyruvate for gluconeogenesis rather than respiration^79^. Interestingly, some studies have reported that acute starvation, unlike prolonged or intermittent fasting, increases H_2_O_2_ production in cell culture and in mice^80^, raising the possibility that fasting might down-regulate PDH activity in part by increasing its oxidation. To determine whether DLAT oxidation is modulated by fasting, we compared DLAT oxidation across different tissues from wild-type mice that were either fed ad libitum or fasted for 16 hours. (Fig. 5g). This revealed that DLAT oxidation is present in all wildtype tissues under control conditions, and that it increases in response to fasting in the kidney, liver and muscle. Thus, DLAT oxidation can be physiologically modulated in vivo.

### PDH activity correlates with DLAT oxidation across human samples

**Fig. 6:**
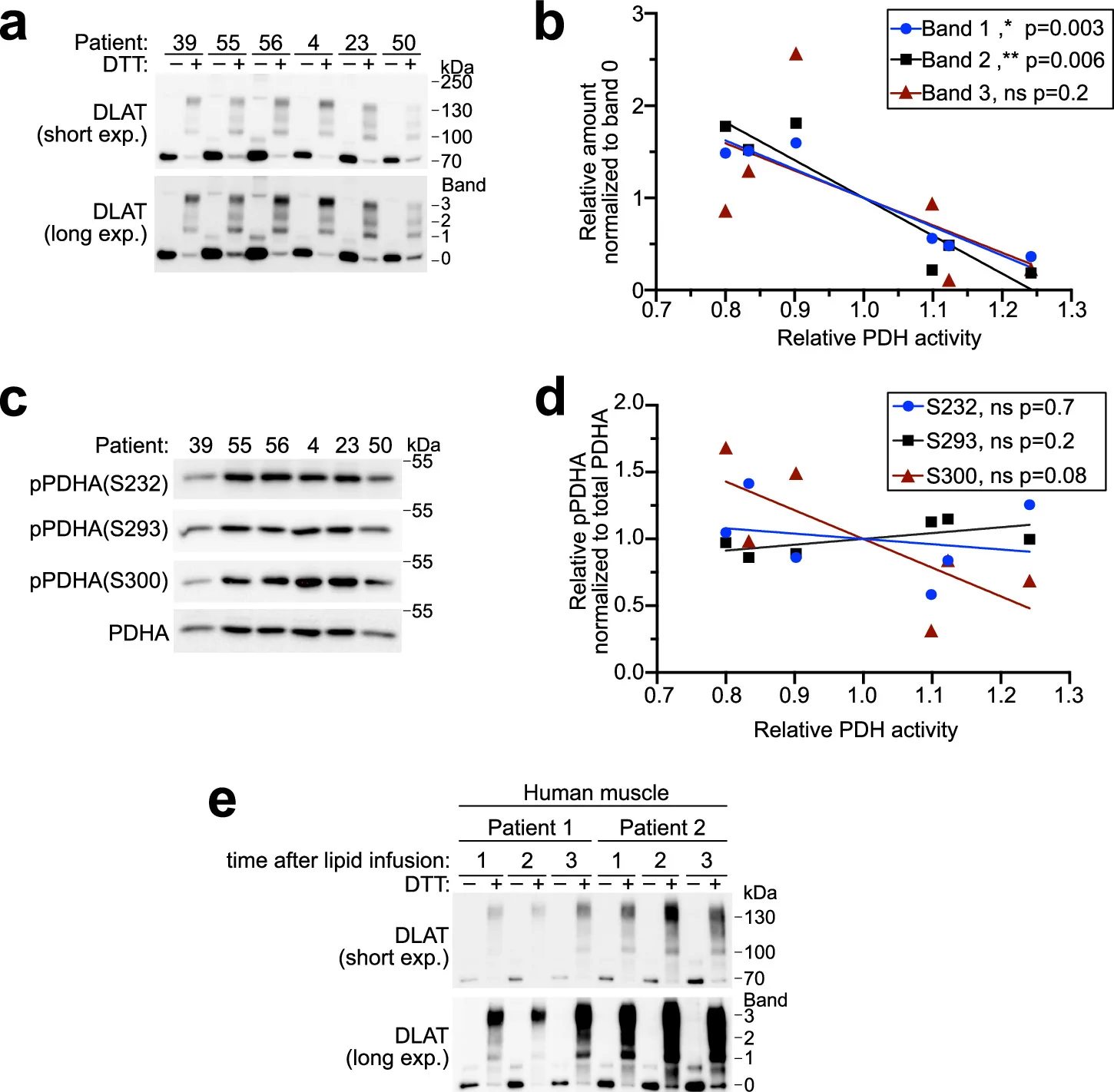
DLAT oxidation correlates more significantly with PDH activity in human liver compared to PDH phosphorylation. For panels a-d the same liver samples from n = 6 patients with obesity and diabetes were used to study DLAT oxidation (a), PDH activity (b and d) and phosphorylation (b). **(a)** DLAT is oxidized in liver samples from patients with obesity and diabetes with high HOMA-IR. **(b)** DLAT oxidation correlates significantly with PDH activity in liver samples from patients with obesity and diabetes. Significance by Pearson’s correlation coefficient (r) with a two-tailed test.**(c, d)** Phosphorylation of PDHA on Ser300 in livers of patients with obesity and diabetes correlates with PDH activity, although not significantly (d). Significance by Pearson’s correlation coefficient (r) with a two-tailed test. **e** PDH is highly oxidized in human muscles and does not change oxidation state in response to lipid or insulin infusion. Muscle biopsies were taken (1) before the clamp, in the fasting state, (2) 2.5 h after start of lipid infusion, and 3) 4 hours after the start of the lipid infusion and 30 minutes after the start of a high-insulin clamp. *n* = 2 patients. Source data are provided as a Source Data file.

We next asked whether DLAT is oxidized in vivo in human tissue. Since individuals with diabetes often have elevated levels of reactive metabolites such as MG^35,36^, we measured DLAT oxidation in liver samples from patients with only pre-diabetes or pre-diabetes and obesity having a high HOMA-IR (Supplementary Data 2). Indeed, DLAT oxidation is highly prevalent in human liver (Fig. 6a). By quantifying DLAT oxidation and PDH activity in parallel from these same samples, we observed a significant negative correlation between the degree of DLAT oxidation and PDH activity across individuals (Fig. 6b). Also phosphorylation of PDH on S300 showed a correlation with PDH activity, although not significant (Fig. 6c-d). These findings indicate that DLAT oxidation is highly prevalent in liver, and could be an important regulator and readout of PDH activity in vivo in humans.

### DLAT is highly oxidized in muscle

Since skeletal muscle is another critical tissue for glucose homeostasis^81^, we assayed PDH oxidation in human muscle. We tested muscle from healthy individuals in three metabolic states – a fasted state, after 2.5 h of lipid infusion, and 30 min after a hyperinsulinemic clamp – to test if PDH oxidation responds to these stimuli. Interestingly, we find that DLAT is very highly oxidized in skeletal muscle (Fig. 6e), with very little DLAT protein remaining unoxidized (band 0). We did not observe a change in DLAT oxidation in response to lipids or insulin, suggesting that muscle has constitutively high DLAT oxidation. This high level of DLAT oxidation aligns well with the fact that skeletal muscle has low PDH activity compared to other tissues^82^.

## Discussion

In this study, we show that DLAT, a component of the PDH complex, becomes oxidized on three cysteines – Cys488, Cys586 and Cys618 – in response to oxidative stress. This oxidation reduces PDH activity, promoting a Warburg-like metabolic phenotype characterized by increased glycolysis, elevated lactate production, and reduced TCA cycle activity. In agreement with this mechanism, re-routing glycolytic flux to the pentose phosphate pathway, cells with mutant DLAT, which cannot be oxidized, have reduced NADPH production, reduced H_2_O_2_ detoxification, and reduced survival to H_2_O_2_. In sum, oxidation of DLAT on Cys488, Cys586 and Cys618 is part of a homeostatic mechanism in which oxidative stress enhances NADPH production, helping cells to counteract the oxidative environment.

Our findings expand our understanding of PDH complex regulation beyond phosphorylation events, revealing that PDH can also sense the oxidative state of a cell directly through DLAT oxidation. We find that DLAT oxidation is present in vivo in both mouse and human. Indeed, in some tissues, such as mouse and human muscle, the majority of DLAT is oxidized (Figs. 5g, 6e), suggesting that this mode of regulation is widespread in vivo. Oxidation of DLAT may explain why in some cases PDH phosphorylation and PDH activity do not correlate^83^. Indeed, our results highlight the importance of directly measuring PDH activity rather than relying on phosphorylation as a proxy for activity, which is often done due to the relative ease of measuring phosphorylation.

Given the prevalence of DLAT oxidation in regulating PDH activity, it will be interesting in the future to study its broader implications for physiology and pathophysiology. This will necessitate the generation of mouse models with point mutations at DLAT Cys488, Cys586 and Cys618. DLAT oxidation is likely to be relevant in various contexts: (1) Since different tissues have different levels of DLAT oxidation (Fig. 5g), it will be interesting to study whether this contributes to tissue-specific differences in PDH activity^82^. (2) By rerouting metabolic flux, DLAT oxidation could impact basal metabolism and the ability of an organism to shift between fuel sources, a process that depends on dynamic changes in PDH activity^84^. (3) Individuals with diabetes, as well as mice with experimental diabetes, have reduced PDH activity^9,10^, although the underlying molecular mechanisms are not fully elucidated. Since individuals with diabetes have elevated levels of reactive metabolites such as MG^35,36^ and elevated oxidative stress^85^, oxidation of DLAT could provide a molecular mechanism contributing to the reduced PDH activity and reduced metabolic flexibility that are hallmarks of the disease^86^. Indeed, in muscle from type 2 diabetic mice compared to control mice, PDH activity responds more slowly to DCA, an inhibitor of PDKs that increases PDH activity^87^, suggesting the existence of an additional mechanism that inhibits PDH in diabetes besides phosphorylation. 4) Since type 2 diabetes is a risk factor for certain cancers^88^, DLAT oxidation may contribute to the Warburg-like metabolic phenotype, which provides a favorable metabolic environment for cancer initiation or progression.

In conclusion, we uncover here a mechanism of regulation of PDH that is highly prevalent in vivo and correlates strongly with PDH activity across human samples. DLAT oxidation at Cys488, Cys586 and Cys618 is likely to have far-reaching implications in various physiological and pathological contexts, given the central role that the PDH complex plays in regulating metabolic flux.

## Methods

### Antibodies and reagents

The following primary antibodies were used at 1:1000 dilution unless otherwise specified: CEL (Cell Signaling Technology #44358), DLAT (Cell Signaling Technology #12362), DLAT (Proteintech, #13426-1-AP), DLD (Santa Cruz Biotechnology sc-365977), E3BP (Santa Cruz Biotechnology sc-393644), FLAG tag (Cell Signaling Technology #14693), GAPDH (Cell Signaling Technology #2118), Glutathione (D8) (Santa Cruz Biotechnology sc-52399), HA tag (Cell Signaling Technology #2367), Hyperoxidized PRDX3 (Cayman Chemicals Cay39806-100), LDH (Cell Signaling Technology #2012), MTC1 (Cell signaling Technology #85680), PDHA (Cell Signaling Technology #3205), MG-H1^51^, PDHB (Proteintech #14744-1-AP), pPDH(S232) (Proteintech #29582-1-150), pPDH(S297) (Abcam ab92696) was used 1:5000, pPDH(S232) (Proteintech #80572-1-RR), Pyruvate dehydrogenase E2/E3bp (Abcam ab110333), PRDX3 (Proteintech #10664-1-AP), S-Nitroso-Cys (Biozol Diagnostica APD NISC11-A), Tubulin (Sigma Aldrich #T9026) was used 1:5000, and VDAC (Cell Signaling Technology #4661). Secondary antibodies were used 1:10,000 and are the following: anti-rabbit HRP (Jackson ImmunoResearch #111-035-003), anti-mouse HRP (Jackson ImmunoResearch #115-035-003), anti-guinea pig HRP (Jackson ImmunoResearch #106-035-003).

### Mice

All animal experiments were approved by the ethical authorities (Regierungspräsidium Karlsruhe, Germany) and performed in compliance with the German Animal Welfare Act for the care and use of laboratory animals, with the rules of the regulatory authorities in Baden-Wurttemberg, with the ARRIVE guidelines and with the guidelines of Directive 2010/63/EU.

Experiments with the GLO1^KO^ mice were approved by the ethical authorities (Regierungspräsidium Karlsruhe, Germany), approval numbers 35-9185.81/G-144/21. Chronic hypercholesterolemia/hyperglycemia (HGHCi) was induced in male C57BL/6 N and Glo1-/-^47^ mice. The mice were treated with Adeno-associated viral vectors encoding the gain-of-function variant D377Y of murine PCSK9 (rAAV8-PCSK9^D377Y^) under control of a liver-specific promoter, which were delivered via a single retro-orbital sinus injection (1.0 × 10^11^ viral genomes/mouse). To induce chronic hypercholesterolemia, the animals were fed a high-cholesterol/high-fat diet containing 16% fat and 1.25% cholesterol. To induce chronic hyperglycemia, mice were injected intraperitoneally with streptozotocin (STZ, 60 mg/kg, freshly dissolved in 0.05 M sterile sodium citrate, pH 4.5), once daily for five consecutive days, starting one week after rAAV8-PCSK9^D377Y^ application. Blood glucose levels were monitored twice per week using Accu-Chek Aviva (Roche, USA) and maintained in a range of 300–500 mg/dl. Body weight was measured once weekly. HFD and hyperglycemia (minimum 300 mg/dl) were maintained for 6 weeks. Mice were then sacrificed by cervical dislocation. Liver tissue was washed briefly in ice-cold NaCl and immediately snap frozen in liquid nitrogen.

For assaying DLAT oxidation in different tissues of control mice, C57BL/6 N mice were purchased from Charles River Laboratories (Wilmington, MA, USA). The mice were maintained under standard laboratory conditions of a 12 h light/12-hour dark cycle (22 °C, 55% humidity) and had access to food and water ad libitum. 10-week-old C57BL/6 N male mice were fasted for 16 h or not, and then sacrificed using carbon dioxide, followed by perfusion with ice-cold PBS. Major organs (brain, heart, liver, kidneys, colon, epididymal fat pads, and quadriceps muscle) were dissected and flash-frozen in liquid nitrogen. All procedures were approved by the Animal Care and Use Committee at the regional authority in Karlsruhe, Germany (T-94/14).

For the xenograft experiments, 10-week-old NSG/NXG female mice were purchased from Janvier Labs (Rte du Genest, Le Genest-Saint-Isle, France). One million cells were suspended in a PBS-Matrigel mixture (1:1, v/v) and injected subcutaneously into the posterior right flank of NSG/NXG mice anesthetized with isoflurane. Tumor growth was measured regularly with calipers 2-3 times per week in two dimensions after cell injection to quantify tumor volume (length x width²). Mice were examined daily for general health, behavior, and signs of discomfort, in accordance with institutional and German animal welfare regulations. All procedures were approved by the Animal Care and Use Committee at the regional authority in Karlsruhe, Germany (permit 35-9185.81/G-74/25).

### Human liver samples

Human liver samples were obtained from the ChARMeD study, a prospective longitudinal cohort that follows individuals before and up to two years after bariatric surgery. The study is currently being conducted at the Study Center for Diabetes and Metabolism at the Department of Endocrinology, Diabetology, Metabolic Diseases and Clinical Chemistry at the University Hospital Heidelberg. Surgeries and liver biopsy collections were performed at the Department of General, Visceral, and Transplantation Surgery, University Hospital Heidelberg. The study was approved by the ethics committee of Heidelberg University, in compliance with national guidelines and the Declaration of Helsinki (local ethic number S-887/2021) and is registered with the German Clinical Trials Register (DRKS00028484). Participants were prospectively recruited at the Study Center for Diabetes and Metabolism, University Hospital Heidelberg, among individuals scheduled for bariatric surgery who met the indication for surgery according to national guidelines^89^. All participants provided written informed consent prior to study procedures.

All liver samples were from female participants. Participants were clinically and metabolically assessed for obesity- and diabetes-associated complications prior to surgery. Standardized laparoscopic procedures were performed under general anesthesia by experienced surgeons. Liver biopsies, obtained from the lower part of the right liver lobe following standardized protocols^90^, were either formalin-fixed and paraffin-embedded or snap-frozen in liquid nitrogen and stored at − 80 °C for further analysis. Paraffin-embedded samples were histologically evaluated using hematoxylin-eosin and Masson’s trichrome by specialized liver pathologists at the Department of Pathology, University Hospital Heidelberg. MASLD was assessed using the steatosis, activity, and fibrosis score^91^. Snap-frozen liver samples were further processed for PDH activity, acyl-PEG modification and inmunoblot assessment.

Anthropometric and metabolic characteristics of study participants are shown in Supplementary Data 2.

Muscle biopsies were assessed from healthy volunteers participating in the FFAIR study. Muscle biopsies were taken (1) before the clamp, in the fasting state, (2) 2.5 h after start of the lipid infusion, and (3) 4 h after the start of the lipid infusion and 30 minutes after the start of a high-insulin clamp to examine insulin stimulation of PDH activity. The region above the musculus vastuslateralis was anesthetized with local anesthetics (Xylocain 2%), a muscle biopsy of ∼ 70–400 mg was obtained using a Bergstrom needle. Samples were immediately stored in liquid nitrogen and then frozen at − 80 °. The study was approved by the ethics committee of Heidelberg University, in compliance with national guidelines and the Declaration of Helsinki (local ethic number S-883/2021) and is registered with the German Clinical Trials Register (DRKS00028505). Healthy volunteers were prospectively recruited for the FFAIR study at the Study Center for Diabetes and Metabolism, University Hospital Heidelberg, and provided written informed consent prior to study procedures.

### Cell culture

The following cell lines were used: HEK293T (ATCC #CRL-3216), HeLa (ATCC #CCL-2), HepG2 (ATCC #HB-8065), All cell lines were cultured in DMEM (Life Technologies #41965-062) containing 25 mM glucose, 4 mM L-glutamine, 1% penicillin/streptomycin (Life Technologies #15140-122), and 10% FBS. The culture conditions were 5% CO2 at 37 °C, and the cells were tested for mycoplasma to exclude contamination.

For all treatments with metabolites or inhibitors, cells were seeded the day before treatment and the addition was performed directly to the plate or well for 1 hour unless otherwise specified. The following metabolites or inhibitors were used: MG was synthesized as described in ref. ^42^, H_2_O_2_ (Sigma Aldrich # 516813), trolox (Sigma Aldrich #238813), sodium dichloroacetate (DCA) (Sigma Aldrich 347795) and 3-Amino-1,2,4-triazol (3AT) (Sigma Aldrich #A8056).

### Plasmid transfections and siRNA knockdowns

For all transfections, the manufacturer’s instructions were followed, and the cells were always seeded one day in advance. HeLa cell transfections were done with Lipofectamine 2000 reagent (ThermoScientific #11668030), while for HEK293T cells, we transfected with PEI (Polysciences #23966-1).

All siRNA-mediated knockdowns were performed by reverse-transfection with 15 nM pools of four siRNAs targeting either human GLO1 (siGENOME, Horizon Discovery #MU-012277-01-0002), human DLAT (siGENOME, Horizon Discovery #LQ-008490-01-0002) or Renilla luciferase (siGENOME, Horizon Discovery #P-002070-01-50) as a negative control. siRNA sequences are provided in Supplementary Data 3. After waiting three days, the cells were lysed and analyzed by immunoblotting or pyruvate dehydrogenase activity was measured as detailed in the “Enzymatic assays” section below.

### Generation of plasmids, knockout cells and stably transfected cell lines

Monoclonal GLO1 knockout (Glo^KO^) HeLa cells were previously generated and validated by us^58^, while the monoclonal Glo^KO^ HEK293T and DLAT^KO^ cell lines were generated via CRISPR-Cas9-mediated genome editing using the PX459 plasmid expressing both Cas9 and an sgRNA targeting hGLO1 (GCAGACCATGCTACGAGTGA) or hDLAT (AGATGGTAGAATCACCAAGA) according to the Brunello library^92^. Validation of the Glo^KO^ and DLAT^KO^ cell lines was performed by PCR-amplifying the target genomic locus, TOPO cloning (Life Technologies #450640), and sanger sequencing. We sequenced 19 clones for the Glo1^KO^ cell line, and 18 clones for the DLAT^KO^, and they all showed the same mutation as indicated in Supplementary Fig. 1a or Supplementary Fig. 7a.

The human GLO1 ORF, which we previously cloned into pcDNA3.1 ( + )^58^, was subcloned into the same plasmid but with an exchange of the neomycin resistance cassette for puromycin resistance and a C-terminal FLAG-tag was added. We generated the HEK293T cell lines stably expressing FLAG-tagged WT or catalytically inactive (CI) GLO1 by transfection of linearized plasmids into GLO1^KO^ HEK293T cells and puromycin selection. The presence of GLO1 in the cell lines were validated by immunoblotting and GLO1 activity assays. Point mutations in GLO1 were introduced by overlap-PCR site-directed mutagenesis using oligos listed in Suppl. Data 3.

The coding sequences of PDHA1 and DLAT were PCR-amplified from HeLa cDNA using Phusion High-Fidelity Polymerase (New England Biolabs #M0530). These were cloned into pRK5, and when indicated, a tag was added by subcloning the inserts into a pRK5-based plasmids containing the tag of interest, either FLAG or HA. Point mutations in DLAT were introduced by PCR-based site-directed mutagenesis using primers containing overlapping sequences, followed by assembly of the PCR products using Gibson Assembly (New England Biolabs, #E2611S). Oligo sequences are in Suppl. Data 3.

The piggyBAC transposon system^93^ was used to stably transfect DLAT^KO^ HEK293T cells with DLAT WT, the single point mutants of DLAT (C488S), (C586S), (C618S) or the triple mutant (TM) (C488S, C586S, C618S) of DLAT. This was performed by cloning the constructs of interest into a modified piggyBac-T2A-mNeonGreen vector (piggyBac-puro), where the T2A-mNeonGreen sequence was replaced with a multiple cloning site, a BGH polyadenylation signal from pcDNA3.1(+) and an expression cassette for puromycin resistance under control of the SV40 promoter. The original piggyBac-T2A-mNeonGreen plasmid was a kind gift by Moritz Mall (DKFZ Heidelberg). The piggyBAC plasmids and the transposase were transfected into the DLAT^KO^ HEK293T in the ratio 1:1. Starting from two days after transfection, cells were cultured in growth medium supplemented with 1 μg/ml puromycin (Sigma-Aldrich #P9620) for approximately two weeks, until a polyclonal resistant population emerged.

### Cell lysis, immunoblots, immunoprecipitations

Cell lysis was a three step procedure: First the cells were washed with Dulbecco’s phosphate buffer saline (DPBS) (Life Technologies #14040117), then the cells were scraped with lysis buffer (50 mM Tris pH 7.4, 150 mM NaCl, 1% triton X-100, 1x Roche Complete protease inhibitor cocktail with EDTA, and 1x Roche PhosSTOP phosphatase inhibitors) and finally the lysates were cleared by centrifugation for 10 min, 10000 × *g* at 4 °C. In all cases, the protein concentration was determined using Bradford reagent (Biorad #500-0006).

For immunoprecipitation (IP), two 15 cm dishes were used per condition. The cells were lysed with lysis buffer (40 mM HEPES pH 7.4, 5 mM MgCl_2_, 1% triton X-100, 1x Roche Complete EDTA-free protease inhibitor cocktail, and 1x Roche PhosSTOP phosphatase inhibitors). The lysates were cleared of cell debris by centrifugating for 10 min, 10000 × *g* at 4 °C. For the IP, 4-6 mg of protein in a final volume of 1 ml were incubated with 30 μL pre-washed anti-DYKDDDDK affinity resin beads (ThermoFisher #A36803) or anti-HA magnetic beads (ThermoFisher #88837) for 2 h in rotation. For elution, the immunoprecipitates were washed three times with lysis buffer supplemented with 150 mM NaCl and then eluted with 2x Laemmli.

For pulldown of Biotinylated Glutathione Ethyl Ester (BioGEE), one 10 cm dish was incubated with 0.02 μM BioGEE (ThermoFisher #G36000) for 2 h. Then the cells were washed twice with DPBS and lysed with Tris HCl 25 mM pH 7.4, NaCl 150 mM, MgCl_2_ 5 mM, 1% triton and 10 mM N-ethylmaleimide to block further thiol reactions. The lysates were centrifuged in a tabletop centrifuge at 20800 x *g* 4 C for 10 min and 1-2 mg of protein were incubated with streptavidin-coupled beads (Life Technologies 65001 Dynabeads MyOneStreptavidin C1) for 2 h with gentle rotation. Next, the beads were washed 3 times with wash buffer (Tris HCl 25 mM, NaCl 500 mM, MgCl_2_ 5 mM and 0.1% SDS) and samples were eluted in 2x Laemmli supplemented with 1 mM biotin.

For developing immunoblots, the chemiluminescent signal was generated using the ECL substrates Western Lightning Plus-ECL (PerkinElmer #NEL105001EA) or Supersignal West Femto (ThermoFisher #34095). All immunoblots were imaged using a BioRad Chemidoc system, and quantifications were performed with ImageLab software (Biorad). For all experiments, to combine quantifications across multiple experiments, the band intensity of each sample was normalized by the sum of all samples in one experiment, thereby normalizing for total intensity.

### Cell fractionation and mitochondrial purification

One 10 cm dish was used per condition. The cells were washed once with DPBS and harvested by adding 1 ml of mitochondrial lysis buffer (10 mM Tris-HCl pH 7.5, 1x Roche Complete EDTA-free protease inhibitor cocktail, and 1x Roche PhosSTOP phosphatase inhibitors) and scraping. Cell lysates were homogenized with a pre-cooled glass homogenizer (tight pestle; 20 strokes up and down). Next, 200 µl of 1.5 M sucrose was added to the homogenized lysates and nuclear sedimentation was performed by centrifuging the lysates for 5 min at 1000 × *g* at 4 °C. The supernatant, containing mitochondrial and cytoplasmatic fractions, was transferred to a new Eppendorf tube and centrifuged a second time for 10 min, 10000 × *g* at 4 °C. Then, the pellet containing the mitochondrial faction was resuspended in the buffer of interest according to the assay to be performed. When mouse and human tissues were used for mitochondrial purifications, the same protocol was followed, with the exception that 20–40 mg of tissue was homogenized in 1 ml mitochondrial lysis buffer supplemented with 2 mM EDTA.

### Flow cytometry

For ROS detection, cells were stained with 5 µM CM-H2DCFDA (Invitrogen, Thermo Fisher Scientific, #C6827) at 37 °C for 30 min in a 12-well plate. When cells were incubated with 100 µM methylglyoxal (MG) for 1 h, the staining was performed in parallel during the last 30 min of the treatment. After staining, the cells were rinsed briefly with PBS and detached with TrypLe (Gibco, Thermo Fisher Scientific). The cell suspension was then diluted with DPBS and analyzed using the Guava easyCyte 14HT flow cytometer (Cytek Biosciences). Fluorescence was measured in the Green-B channel (excitation 488 nm, emission 512/18 nm), with 10,000 cells counted per sample. Background-corrected mean fluorescence intensities were used for statistical analysis.

### Enzymatic assays

The pyruvate dehydrogenase activity assays in HeLa cells were performed using a commercially available colorimetric activity assay kit (Sigma Aldrich MAK183), while the pyruvate dehydrogenase activity in HEK293T cells was measured by an assay protocol adapted from Chretien et al.^94^. In both cases the mitochondrial pellet was resuspended and incubated for 10 minutes with either commercially provided lysis buffer (sigma assay) or PDH sample buffer (10 mM KH2PO4, 5 mM MgCl2, 10% triton, and 1x Roche Complete protease inhibitor cocktail with EDTA). The mitochondrial lysates were cleared by a short spin in a table top Eppendorf centrifuge (1000 g, 2 s). For the adapted PDH activity assay protocol, 10 µl of mitochondrial lysate supernatant was combined with 170 µl of assay buffer (10 mM potassium phosphate buffer, pH 7.4, 10 mM KCl, 5 mM MgCl_2_, 0.5 mM NAD + , 0.2 mM Coenzyme A, 0.2 mM Thiamine pyrophosphate, 1 mM Cysteine and 0.2 mM GNE-140). GNE-140 is an LDH inhibitor, which was included in the assay buffer to prevent the competing reaction which consumes NADH. Then, either 20 µl of pyruvate 4 mM (final concentration 0.4 mM) or 20 µl H_2_O (as a control) were added, and NADH production was measured by monitoring absorbance at 340 nm for 60 min. The assay is specific for PDH activity because it gives no detectable signal in DLAT knockout cells (Supplementary Fig. 7c).

For in vitro assays using pyruvate dehydrogenase from porcine heart (Sigma, P7032-10UN), 26 µg of PDH was incubated with varying concentrations of H_2_O_2_ in a final volume of 100 µl of PDH sample buffer for 1 h. The enzymatic activity was then measured as described above.

GAPDH and KGDH activity were measured with commercially available colorimetric assays, GAPDH (Sigma Aldrich MAK277) and KGDH (Sigma Aldrich MAK189). For GAPDH and KGDH activity measurements, one 10-cm dish was used for cellular fractionation as described above, and the cytosolic supernatant was use for GAPDH activity assays while the mitochondrial fraction was used for KGDH assays.

GLO1 activity was measured from the cytosolic fraction of a well in a 6-well plate. Cells were seeded in a 6-well plate the day before the experiment. Homogenization was performed with 400 μL buffer (10 mM sodium phosphate buffer pH 7.0, 0.02% Triton X-100) and sonication using a Branson sonifier for 15 sec with 10% amplitude, followed by centrifugation at 20800 x *g* for 30 min at 4 C. Substrate mix (50 mM sodium phosphate buffer pH 6.6, 20 µM methylglyoxal (Sigma M0252), 20 µM reduced L-Glutathione (Sigma G6013)) was incubated in a 96-well microtiter plate at 37 C for 10 min. 10 µl of the cleared lysates were then added to 190 uL substrate mix in each well. Changes in absorbance at 235 nm were then measured kinetically for 15 min using the SPECTROstar Omega plate reader (BMG LABTECH).

All enzymatic assays were performed in a 96-well plate format, using the SPECTROstar Omega plate reader (BMG LABTECH). Enzymatic activity was normalized to total mitochondrial protein or to total cellular protein, measured by Bradford assay, depending on whether it is a mitochondrial enzyme or not. In addition, in order to easily compare the results from assays performed on different days, the activity of each sample was normalized to the sum of activity in all samples in that experiment (total signal).

### Glucose uptake and lactate secretion measurements

Cells were seeded in a 10 cm dish on day 0 of the experiment. On day 1, the medium was replaced with fresh medium. For glucose uptake, a sample of this fresh medium was collected, and another sample was taken after 24 h. Glucose was measured in both samples with a colorimetric assay kit (Abcam ab65333), and glucose consumption was determined as the difference in glucose content in the well, normalized to total cell protein in the well. Similarly, extracellular lactate measurements were performed with a commercial kit that can distinguish D-lactic acid from L-lactic acid (R-Biopharm #1112821035) following the manufacturer’s instructions. Extracellular lactate was measured on day 2 (24 h after replacing the medium) by measuring lactate levels in an aliquot of medium. All lactate and glucose measurements were normalized to total protein levels measured by Bradford.

### Metabolic characterization by Seahorse XF assays

A Seahorse XFe96 analyzer was used to measure the glycolytic and respiratory capacity of cells. For this purpose, both the Glycolysis Stress Test Kit (Agilent Seahorse XF, 100-103020) and the Mito Stress Test Kit (Agilent Seahorse XF, 100-103015) were performed following the manufacturer’s instructions. All Seahorse plates were coated with fibronectin solution (Sigma F1141). For ECAR measurements, 35000 cells were seeded per well, as HEK293T are highly glycolytic. For OCR, 45000 cells were seeded per well. Prior to all SeaHorse measurements, the cells were pre-incubated for 1 hour in SeaHorse XF DMEM medium (Agilent Seahorse XF, 103575-100) supplemented with 25 mM Glucose, 2 mM Glutamine and 1 mM pyruvate. For the Bis-2-(5-phenylacetamido-1,3,4-thiadiazol-2-yl)ethyl sulfide (BPTES) (Sigma SML0601) treatments, cells were incubated with a final concentration 3 µM 1 h prior to the assay. In addition, a parallel 96-well plate was always seeded to normalize SeaHorse measurements to cell number. For this, nuclear were counted by with Hoechst staining in a Synentec NYONE cell imager.

### Metabolomic flux analysis

Sample extraction: Frozen cells were extracted directly on petri dishes by adding 1 mL 50% MeOH containing 20 µl Ribitol (0,2 mg/ml) as internal standard. Cell slurry was collected with a plastic scraper and transferred to 2-mL Eppendorf tubes on ice. After the addition of 500 µL 100% chloroform, samples were vortexed and further incubated on ice for 5 min. To separate polar and organic phases, samples were centrifuged for 10 min at 11,000 x *g*. While avoiding the interphase containing cellular debris, 400 µL of the polar (upper) phase were transferred to a fresh glass vial and dried using a vacuum concentrator (Eppendorf Concentrator Plus) without heating.

Derivatization (Methoximation and Silylation): Sequential on-line methoximation and silylation reactions were performed using a MPS autosampler (Gerstel, Mülheim Ruhr). Methoximation was performed by adding 20 µL 20 mg/ml methoxyamine hydrochloride (Sigma 226904) in pyridine (Sigma 270970) and incubation at 37 °C for 90 min in a Gerstel MPS Agitator Unit (250 rpm). For silylation reactions, 45 µl of N-Methyl-N-(trimethylsilyl)trifluoroacetamide (MSTFA; Sigma 69479) were added, and samples were incubated at 37 °C for 30 min with gentle shaking. Before injection, samples were incubated at RT for 45 min.

Gas Chromatography/Mass Spectrometry (GC/MS) analysis: For GC/MS analysis, a GC-ToF system was used, consisting of an Agilent 7890 Gas CHromatograph (Agilent, Santa Clara) fitted with a Rxi-5Sil MS column (30 meter x 0.25 mm × 0.25 µm; Restek Corporation, Bellefonte) coupled to a Pegasus BT Mass Spectrometer (LECO Corporation, Michigan). The GC was operated with an injection temperature of 250 °C, and 1 µL sample was injected in splitless mode. The GC temperature program started with a 1 min. hold at 40 °C followed by a 6 °C/min ramp up to 210 °C, a 20 °C/min ramp up to 330 °C and a bake-out at 330 °C for 5 min. using Helium as a carrier gas with constant linear velocity. The ToF mass spectrometer was operated with ion source and interface temperatures of 250 °C, a solvent cut time of 12 min and a scan range (m/z) of 50 – 600 with an acquisition rate of 17 spectra/second.

Mass isotopologue distribution (MID) was determined using the free DExSI software^95^.

### PEG exchange assay

For all our PEG exchange assays, we modified the method described by Percher et al.^96^ to asses oxidation instead of acylation. First, cells were seeded in a 6-well plate, and after one day the cells were washed twice with DPBS and lysed with 250 µl of TAE lysis buffer (5 mM thioethanolamine, 15 mM NaCl, 4% SDS, 5 mM PMSF, 1x Roche Complete EDTA-free protease inhibitor cocktail, and 1500 units/ml benzonase, pH 7.3). Then, 92.5 μl of cell lysate were pipetted into a new Eppendorf tube in duplicate, and the lysates were supplemented with EDTA to a final concentration of 5 mM. Next, 2.5 μl of 1M N-ethylmaleimide stock were added to the lysates and a 2 h incubation was performed with head-over-tail rotation. The NEM in the lysates was removed by 1 round of chloroform-methanol precipitation and two rounds of washing with methanol. The cleaned pellet was dried at room temperature for 10 min and then resuspended in 60 µl TAE buffer (5 mM thioethanolamine, 15 mM NaCl, 5 mM EDTA and 4% SDS, pH 7.3). Subsequently, 6 µl of 300 mM DTT or TAE-EDTA buffer (negative control) was added to the lysates and a second incubation was performed for 30 minutes. Next, the DTT in the lysates was removed by one round of chloroform-methanol precipitation and two rounds of methanol washes, followed by drying the pellet for other 10 min. The pellet was resuspended this time in 30 µl of TAE buffer and 90 µl of 1.33 mM methoxy polyethylene glycol maleimide (mPEG-mal) 5 kDa. The last incubation was performed for 2 h with gentle rotation, followed by another round of chloroform-methanol precipitation and methanol washes. Finally, the pellet was dried for 10 min and resuspended in 60 μl 2 x Laemmli (control samples) and 30 µl 2 x Laemmli (DTT -treated samples).

### Assessment of reduced/oxidized glutathione (GSH/GSSG) and nicotinamide adenine dinucleotide phosphate (NADPH/NADP^+^) ratios

To determined cellular redox status, GSH/GSSG and NADPH/NADP^+^ ratios were measured using the GSH/GSSG-Glo™ Assay Kit (Promega, V6611) and the NADP/NADPH-Glo™ Assay Kit (Promega, G9082), respectively, accordingly to the manufacturer’s instructions with minor modifications as described below.

A total of 1.2 million cells were seeded per well in a 6-well plate. For GSH/GSSG measurements, two wells were used per condition, one for total glutathione (GSH + GSSG) and one for oxidized glutathione (GSSG) quantification. Prior to measurement, cells were either left untreated or exposed to 400 µM H_2_O_2_ for 1 h to induce oxidative stress. Following treatment, plates were placed on ice for 10 min. During this incubation, N-ethylmaleimide (NEM) was added to wells designated for oxidized glutathione quantification to a final concentration of 25 mM to derivatize and block reduced GSH.

After the 10 min incubation, the medium was removed by gently inverting the plate and tapping it on tissue paper to eliminate residual liquid. The appropriate lysis buffer provided in each assay kit was added to each well. To account for the increase in cell number and surface area compared with the standard 96-well format, the lysis volume was scaled up. For GSH/GSSG measurements, 200 µL of lysis buffer was added to each well, while for NADPH/NADP^+^ measurements, the lysis buffer was scale up to 300 μl per well. All subsequent steps were performed according to the manufacturer’s protocols without further modification.

### Measurements of H_2_O_2_ detoxification capacity and cell viability

To assess the H_2_O_2_ detoxification capacity of DLAT^KO^ HEK293T cells stably transfected with WT and TM DLAT, 1.2 million cells were seeded in a 6-well plate on day 0 of the experiment. The next day 400 µM H_2_O_2_ was added to the cells, and the level of H_2_O_2_ in the medium were measured after 30 min had passed using the Peroxide assay kit (Sigma MAK311-1KT) following the manufacturer’s instructions. All measurements were normalized to total protein levels determined by Bradford.

The cell viability of DLAT^KO^ HEK293T cells stably transfected with WT and TM DLAT was assessed by seeding 45.000-55.000 cells per well in a 96-well plate on day 0. On day 1, cells were treated with 400 µM H_2_O_2_, and viability was evaluated 24 h later by nuclear counting using Hoechst staining. Image acquisition and quantification were performed using a Synentec NYONE cell imager.

### Quantification of MG by mass spectrometry

Levels of free methylglyoxal in HEK293T cells were determined by isotope dilution, and tandem mass spectroscopy, following derivatization with 1,2-diaminobenzene as described in ref. ^97^.

### Statistical analyses

All the data were visualized and analyzed using GraphPad Prism (version 9.5.0) and Microsoft Excel (version16.16.27). When two conditions were compared, two-tailed, unpaired Student’s *t* tests were used unless specified otherwise. When comparing more conditions, one- or two-way ANOVA was used with *Šídák’s* correction for multiple testing.

### Reporting summary

Further information on research design is available in the Nature Portfolio Reporting Summary linked to this article.

## Supplementary information

Supplementary Information

Description of Additional Supplementary Files

Supplementary Data 1

Supplementary Data 2

Supplementary Data 3

Reporting Summary

Transparent Peer Review file

## Source data

Source Data

## Data Availability

All data supporting the findings of this study are available within the paper and its Supplementary Information. Source data are provided in this paper.

## References

[1] Patel, M. S., Nemeria, N. S., Furey, W. & Jordan, F. The pyruvate dehydrogenase complexes: structure-based function and regulation. *J. Biol. Chem.* **289**, 16615–16623 (2014). 10.1074/jbc.R114.563148 PMC4059105 24798336

[2] Reed, L. J. & Oliver, R. M. The multienzyme alpha-keto acid dehydrogenase complexes. *Brookhaven Symp. Biol.* **21**, 397–412 (1968). 4888137

[3] Kim, M. J. et al. The role of pyruvate metabolism in mitochondrial quality control and inflammation. *Mol. Cells* **46**, 259–267 (2023). 10.14348/molcells.2023.2128 PMC10183795 36756776

[4] McFate, T. et al. Pyruvate dehydrogenase complex activity controls metabolic and malignant phenotype in cancer cells. *J. Biol. Chem.* **283**, 22700–22708 (2008). 10.1074/jbc.M801765200 PMC2504897 18541534

[5] Gopal, K. et al. Loss of muscle PDH induces lactic acidosis and adaptive anaplerotic compensation via pyruvate-alanine cycling and glutaminolysis. *J. Biol. Chem.* **299**, 105375 (2023). 10.1016/j.jbc.2023.105375 PMC10692893 37865313

[6] Chambers, K. T. et al. Chronic inhibition of pyruvate dehydrogenase in heart triggers an adaptive metabolic response. *J. Biol. Chem.* **286**, 11155–11162 (2011). 10.1074/jbc.M110.217349 PMC3064169 21321124

[7] Zhang, S., Hulver, M. W., McMillan, R. P., Cline, M. A. & Gilbert, E. R. The pivotal role of pyruvate dehydrogenase kinases in metabolic flexibility. *Nutr. Metab.* **11**, 10 (2014). 10.1186/1743-7075-11-10 PMC3925357 24520982

[8] Befroy DE et al. Impaired mitochondrial substrate oxidation in muscle of insulin-resistant offspring of type 2 diabetic patients. *Diabetes* **56**, 1376–1381 (2007). 10.2337/db06-0783 PMC2995532 17287462

[9] Zhou, Y. P., Ostenson, C. G., Ling, Z. C. & Grill, V. Deficiency of pyruvate dehydrogenase activity in pancreatic islets of diabetic GK rats. *Endocrinology* **136**, 3546–3551 (1995). 10.1210/endo.136.8.7628391 7628391

[10] Haythorne, E. et al. Altered glycolysis triggers impaired mitochondrial metabolism and mTORC1 activation in diabetic beta-cells. *Nat. Commun.* **13**, 6754 (2022). 10.1038/s41467-022-34095-x PMC9663558 36376280

[11] Jeon, J. H. et al. Loss of metabolic flexibility as a result of overexpression of pyruvate dehydrogenase kinases in muscle, liver and the immune system: Therapeutic targets in metabolic diseases. *J. Diab. Investig.* **12**, 21–31 (2021). 10.1111/jdi.13345 PMC7779278 32628351

[12] Feron, O. Pyruvate into lactate and back: from the Warburg effect to symbiotic energy fuel exchange in cancer cells. *Radiother. Oncol.* **92**, 329–333 (2009). 10.1016/j.radonc.2009.06.025 19604589

[13] Heigenhauser, G. J. & Parolin, M. L. Role of pyruvate dehydrogenase in lactate production in exercising human skeletal muscle. *Adv. Exp. Med. Biol.* **474**, 205–218 (1999). 10.1007/978-1-4615-4711-2_17 10635003

[14] Ralser, M. et al. Dynamic rerouting of the carbohydrate flux is key to counteracting oxidative stress. *J. Biol.* **6**, 10 (2007). 10.1186/jbiol61 PMC2373902 18154684

[15] Kuehne, A. et al. Acute activation of oxidative pentose phosphate pathway as first-line response to oxidative stress in human skin cells. *Mol. Cell* **59**, 359–371 (2015). 10.1016/j.molcel.2015.06.017 26190262

[16] Saunier, E., Benelli, C. & Bortoli, S. The pyruvate dehydrogenase complex in cancer: An old metabolic gatekeeper regulated by new pathways and pharmacological agents. *Int. J. Cancer* **138**, 809–817 (2016). 10.1002/ijc.29564 25868605

[17] Woolbright, B. L., Rajendran, G., Harris, R. A. & Taylor, J. A. 3rd. Metabolic flexibility in cancer: targeting the pyruvate dehydrogenase kinase:pyruvate dehydrogenase axis. *Mol. Cancer Ther.* **18**, 1673–1681 (2019). 10.1158/1535-7163.MCT-19-0079 31511353

[18] Chen, J. et al. Compartmentalized activities of the pyruvate dehydrogenase complex sustain lipogenesis in prostate cancer. *Nat. Genet.* **50**, 219–228 (2018). 10.1038/s41588-017-0026-3 PMC5810912 29335542

[19] Anastasiou, D. et al. Inhibition of pyruvate kinase M2 by reactive oxygen species contributes to cellular antioxidant responses. *Science* **334**, 1278–1283 (2011). 10.1126/science.1211485 PMC3471535 22052977

[20] Gruning, N. M. et al. Pyruvate kinase triggers a metabolic feedback loop that controls redox metabolism in respiring cells. *Cell Metab.* **14**, 415–427 (2011). 10.1016/j.cmet.2011.06.017 PMC3202625 21907146

[21] Yu, X. et al. Structures of the human pyruvate dehydrogenase complex cores: a highly conserved catalytic center with flexible N-terminal domains. *Structure* **16**, 104–114 (2008). 10.1016/j.str.2007.10.024 PMC4807695 18184588

[22] Hiromasa, Y., Fujisawa, T., Aso, Y. & Roche, T. E. Organization of the cores of the mammalian pyruvate dehydrogenase complex formed by E2 and E2 plus the E3-binding protein and their capacities to bind the E1 and E3 components. *J. Biol. Chem.* **279**, 6921–6933 (2004). 10.1074/jbc.M308172200 14638692

[23] Holness, M. J. & Sugden, M. C. Regulation of pyruvate dehydrogenase complex activity by reversible phosphorylation. *Biochem. Soc. Trans.* **31**, 1143–1151 (2003). 10.1042/bst0311143 14641014

[24] Korotchkina, L. G. & Patel, M. S. Probing the mechanism of inactivation of human pyruvate dehydrogenase by phosphorylation of three sites. *J. Biol. Chem.* **276**, 5731–5738 (2001). 10.1074/jbc.M007558200 11092882

[25] Fan, J. et al. Tyr-301 phosphorylation inhibits pyruvate dehydrogenase by blocking substrate binding and promotes the Warburg effect. *J. Biol. Chem.* **289**, 26533–26541 (2014). 10.1074/jbc.M114.593970 PMC4176253 25104357

[26] Jin, Y. et al. Src drives the Warburg effect and therapy resistance by inactivating pyruvate dehydrogenase through tyrosine-289 phosphorylation. *Oncotarget* **7**, 25113–25124 (2016). 10.18632/oncotarget.7159 PMC5041892 26848621

[27] Mao, Y. et al. Hypoxia induces mitochondrial protein lactylation to limit oxidative phosphorylation. *Cell Res.* **34**, 13–30 (2024). 10.1038/s41422-023-00864-6 PMC10770133 38163844

[28] Palmieri, E. M. et al. Pyruvate dehydrogenase operates as an intramolecular nitroxyl generator during macrophage metabolic reprogramming. *Nat. Commun.* **14**, 5114 (2023). 10.1038/s41467-023-40738-4 PMC10444860 37607904

[29] Ogrunc, M. et al. Oncogene-induced reactive oxygen species fuel hyperproliferation and DNA damage response activation. *Cell Death Differ.* **21**, 998–1012 (2014). 10.1038/cdd.2014.16 PMC4013514 24583638

[30] Yamamori, T. et al. Ionizing radiation induces mitochondrial reactive oxygen species production accompanied by upregulation of mitochondrial electron transport chain function and mitochondrial content under control of the cell cycle checkpoint. *Free Radic. Biol. Med.* **53**, 260–270 (2012). 10.1016/j.freeradbiomed.2012.04.033 22580337

[31] Shalini, S. et al. Impaired antioxidant defence and accumulation of oxidative stress in caspase-2-deficient mice. *Cell Death Differ.* **19**, 1370–1380 (2012). 10.1038/cdd.2012.13 PMC3392626 22343713

[32] Phillips, S. A. & Thornalley, P. J. The formation of methylglyoxal from triose phosphates. Investigation using a specific assay for methylglyoxal. *Eur. J. Biochem.* **212**, 101–105 (1993). 10.1111/j.1432-1033.1993.tb17638.x 8444148

[33] Coukos, J. S., Lee, C. W., Pillai, K. S., Liu, K. J. & Moellering, R. E. Widespread, Reversible Cysteine Modification by Methylglyoxal Regulates Metabolic Enzyme Function. *ACS Chem. Biol.* **18**, 91–101 (2023). 10.1021/acschembio.2c00727 PMC9872086 36562291

[34] Xue, M., Irshad, Z., Rabbani, N. & Thornalley, P. J. Increased cellular protein modification by methylglyoxal activates endoplasmic reticulum-based sensors of the unfolded protein response. *Redox Biol.* **69**, 103025 (2024). 10.1016/j.redox.2024.103025 PMC10821617 38199038

[35] Bierhaus, A. et al. Methylglyoxal modification of Nav1.8 facilitates nociceptive neuron firing and causes hyperalgesia in diabetic neuropathy. *Nat. Med.* **18**, 926–933 (2012). 10.1038/nm.2750 22581285

[36] Rhein, S. et al. The reactive pyruvate metabolite dimethylglyoxal mediates neurological consequences of diabetes. *Nat. Commun.* **15**, 5745 (2024). 10.1038/s41467-024-50089-3 PMC11237006 38987239

[37] Lai, S. W. T., Lopez Gonzalez, E. J., Zoukari, T., Ki, P. & Shuck, S. C. Methylglyoxal and its adducts: induction, repair, and association with disease. *Chem. Res Toxicol.* **35**, 1720–1746 (2022). 10.1021/acs.chemrestox.2c00160 PMC9580021 36197742

[38] Rabbani, N. & Thornalley, P. J. Protein glycation - biomarkers of metabolic dysfunction and early-stage decline in health in the era of precision medicine. *Redox Biol.* **42**, 101920 (2021). 10.1016/j.redox.2021.101920 PMC8113047 33707127

[39] Sruthi, C. R. & Raghu, K. G. Methylglyoxal induces ambience for cancer promotion in HepG2 cells via Warburg effect and promotes glycation. *J. Cell Biochem.* **123**, 1532–1543 (2022). 10.1002/jcb.30215 35043457

[40] Di Loreto, S. et al. Methylglyoxal induces oxidative stress-dependent cell injury and up-regulation of interleukin-1beta and nerve growth factor in cultured hippocampal neuronal cells. *Brain Res.* **1006**, 157–167 (2004). 10.1016/j.brainres.2004.01.066 15051519

[41] Zhang, Y. et al. Methylglyoxal induces oxidative stress and ferroptosis of renal tubular epithelial cells in acute and chronic kidney injury mice. *Front. Cell Dev. Biol.* **13**, 1604575 (2025). 10.3389/fcell.2025.1604575 PMC12492953 41049288

[42] Morgenstern, J. et al. Loss of glyoxalase 1 induces compensatory mechanism to achieve dicarbonyl detoxification in mammalian schwann cells. *J. Biol. Chem.* **292**, 3224–3238 (2017). 10.1074/jbc.M116.760132 PMC5336158 27956549

[43] Oliveira, A. L. et al. Enhanced RAGE expression and excess reactive-oxygen species production mediates Rho kinase-dependent detrusor overactivity after methylglyoxal exposure. *Front. Physiol.* **13**, 860342 (2022). 10.3389/fphys.2022.860342 PMC8996136 35418871

[44] Wang, G. et al. Metformin prevents methylglyoxal-induced apoptosis by suppressing oxidative stress in vitro and in vivo. *Cell Death Dis.* **13**, 29 (2022). 10.1038/s41419-021-04478-x PMC8748764 35013107

[45] Lu, J. et al. Increased plasma methylglyoxal level, inflammation, and vascular endothelial dysfunction in diabetic nephropathy. *Clin. Biochem.* **44**, 307–311 (2011). 10.1016/j.clinbiochem.2010.11.004 21126514

[46] Thornalley, P. J. Glyoxalase I-structure, function and a critical role in the enzymatic defence against glycation. *Biochem. Soc. Trans.* **31**, 1343–1348 (2003). 10.1042/bst0311343 14641060

[47] Schumacher, D. et al. Compensatory mechanisms for methylglyoxal detoxification in experimental & clinical diabetes. *Mol. Metab.* **18**, 143–152 (2018). 10.1016/j.molmet.2018.09.005 PMC6308908 30287091

[48] Lo, T. W., Westwood, M. E., McLellan, A. C., Selwood, T. & Thornalley, P. J. Binding and modification of proteins by methylglyoxal under physiological conditions. A kinetic and mechanistic study with N alpha-acetylarginine, N alpha-acetylcysteine, and N alpha-acetyllysine, and bovine serum albumin. *J. Biol. Chem.* **269**, 32299–32305 (1994). 7798230

[49] Wang, T., Douglass, E. F. Jr., Fitzgerald, K. J. & Spiegel, D. A. A “turn-on” fluorescent sensor for methylglyoxal. *J. Am. Chem. Soc.* **135**, 12429–12433 (2013). 10.1021/ja406077j 23931147

[50] Rabbani, N. & Thornalley, P. J. Dicarbonyls linked to damage in the powerhouse: glycation of mitochondrial proteins and oxidative stress. *Biochem. Soc. Trans.* **36**, 1045–1050 (2008). 10.1042/BST0361045 PMC2639773 18793186

[51] Moraru, A. et al. Elevated levels of the reactive metabolite methylglyoxal recapitulate progression of type 2 diabetes. *Cell Metab.* **27**, 926–934 (2018). 10.1016/j.cmet.2018.02.003 29551588

[52] Eruslanov, E. & Kusmartsev, S. Identification of ROS using oxidized DCFDA and flow-cytometry. *Methods Mol. Biol.* **594**, 57–72 (2010). 10.1007/978-1-60761-411-1_4 20072909

[53] Pan, C., Li, B. & Simon, M. C. Moonlighting functions of metabolic enzymes and metabolites in cancer. *Mol. Cell* **81**, 3760–3774 (2021). 10.1016/j.molcel.2021.08.031 34547237

[54] Ridderstrom, M., Cameron, A. D., Jones, T. A. & Mannervik, B. Involvement of an active-site Zn2+ ligand in the catalytic mechanism of human glyoxalase I. *J. Biol. Chem.* **273**, 21623–21628 (1998). 10.1074/jbc.273.34.21623 9705294

[55] Cardozo, G. et al. Mitochondrial peroxiredoxin 3 is rapidly oxidized and hyperoxidized by fatty acid hydroperoxides. *Antioxidants* **12**, 10.3390/antiox12020408 (2023). PMC9952270 36829967

[56] Moreno-Sanchez, R., Rodriguez-Enriquez, S., Marin-Hernandez, A. & Saavedra, E. Energy metabolism in tumor cells. *FEBS J.* **274**, 1393–1418 (2007). 10.1111/j.1742-4658.2007.05686.x 17302740

[57] Komanetsky, S. M. et al. Proteomic identification of aerobic glycolysis as a potential metabolic target for methylglyoxal in adipocytes. *Nutr. Res.* **80**, 66–77 (2020). 10.1016/j.nutres.2020.06.009 32698053

[58] Cortizo, F. G. et al. The activity of glyoxylase 1 is regulated by glucose-responsive phosphorylation on Tyr136. *Mol. Metab.* **55**, 101406 (2022). 10.1016/j.molmet.2021.101406 PMC8715127 34838714

[59] Korotchkina, L. G. & Patel, M. S. Mutagenesis studies of the phosphorylation sites of recombinant human pyruvate dehydrogenase. Site-specific regulation. *J. Biol. Chem.* **270**, 14297–14304 (1995). 10.1074/jbc.270.24.14297 7782287

[60] Kluza, J. et al. Inactivation of the HIF-1alpha/PDK3 signaling axis drives melanoma toward mitochondrial oxidative metabolism and potentiates the therapeutic activity of pro-oxidants. *Cancer Res.* **72**, 5035–5047 (2012). 10.1158/0008-5472.CAN-12-0979 22865452

[61] Cremers, C. M. & Jakob, U. Oxidant sensing by reversible disulfide bond formation. *J. Biol. Chem.* **288**, 26489–26496 (2013). 10.1074/jbc.R113.462929 PMC3772196 23861395

[62] Leonard, S. E. & Carroll, K. S. Chemical ‘omics’ approaches for understanding protein cysteine oxidation in biology. *Curr. Opin. Chem. Biol.* **15**, 88–102 (2011). 10.1016/j.cbpa.2010.11.012 21130680

[63] Tabatabaie, T., Potts, J. D. & Floyd, R. A. Reactive oxygen species-mediated inactivation of pyruvate dehydrogenase. *Arch. Biochem. Biophys.* **336**, 290–296 (1996). 10.1006/abbi.1996.0560 8954577

[64] Feinstein, R. N., Berliner, S. & Green, F. O. Mechanism of inhibition of Catalase by 3-amino-1,2,4-triazole. *Arch. Biochem. Biophys.* **76**, 32–44 (1958). 10.1016/0003-9861(58)90116-4 13560010

[65] Lennicke, C., Rahn, J., Lichtenfels, R., Wessjohann, L. A. & Seliger, B. Hydrogen peroxide - production, fate and role in redox signaling of tumor cells. *Cell Commun. Signal* **13**, 39 (2015). 10.1186/s12964-015-0118-6 PMC4570748 26369938

[66] Hurd, T. R. et al. Inactivation of pyruvate dehydrogenase kinase 2 by mitochondrial reactive oxygen species. *J. Biol. Chem.* **287**, 35153–35160 (2012). 10.1074/jbc.M112.400002 PMC3471752 22910903

[67] Alan, L. & Scorrano, L. Shaping fuel utilization by mitochondria. *Curr. Biol.* **32**, R618–R623 (2022). 10.1016/j.cub.2022.05.006 35728541

[68] Percher, A. et al. Mass-tag labeling reveals site-specific and endogenous levels of protein S-fatty acylation. *Proc. Natl. Acad. Sci. USA* **113**, 4302–4307 (2016). 10.1073/pnas.1602244113 PMC4843475 27044110

[69] Fu, L., Liu, K., Ferreira, R. B., Carroll, K. S. & Yang, J. Proteome-Wide Analysis of Cysteine S-Sulfenylation Using a Benzothiazine-Based. *Probe. Curr. Protoc. Protein Sci.* **95**, e76 (2019). 10.1002/cpps.76 PMC6342663 30312022

[70] Akter, S. et al. Chemical proteomics reveals new targets of cysteine sulfinic acid reductase. *Nat. Chem. Biol.* **14**, 995–1004 (2018). 10.1038/s41589-018-0116-2 PMC6192846 30177848

[71] Shi, Y., Fu, L., Yang, J. & Carroll, K. S. Wittig reagents for chemoselective sulfenic acid ligation enables global site stoichiometry analysis and redox-controlled mitochondrial targeting. *Nat. Chem.* **13**, 1140–1150 (2021). 10.1038/s41557-021-00767-2 PMC9121980 34531572

[72] Mailloux, R. J. & Treberg, J. R. Protein S-glutathionlyation links energy metabolism to redox signaling in mitochondria. *Redox Biol.* **8**, 110–118 (2016). 10.1016/j.redox.2015.12.010 PMC4731959 26773874

[73] Talwar, D. et al. The GAPDH redox switch safeguards reductive capacity and enables survival of stressed tumour cells. *Nat. Metab.* **5**, 660–676 (2023). 10.1038/s42255-023-00781-3 PMC10132988 37024754

[74] O’Brien, M., Chalker, J., Slade, L., Gardiner, D. & Mailloux, R. J. Protein S-glutathionylation alters superoxide/hydrogen peroxide emission from pyruvate dehydrogenase complex. *Free Radic. Biol. Med.* **106**, 302–314 (2017). 10.1016/j.freeradbiomed.2017.02.046 28242228

[75] Cumming, R. C. et al. Protein disulfide bond formation in the cytoplasm during oxidative stress. *J. Biol. Chem.* **279**, 21749–21758 (2004). 10.1074/jbc.M312267200 15031298

[76] Nakamura, H. & Takada, K. Reactive oxygen species in cancer: Current findings and future directions. *Cancer Sci.* **112**, 3945–3952 (2021). 10.1111/cas.15068 PMC8486193 34286881

[77] Saed, C. T., Tabatabaei Dakhili, S. A. & Ussher, J. R. Pyruvate dehydrogenase as a therapeutic target for nonalcoholic fatty liver disease. *ACS Pharm. Transl. Sci.* **4**, 582–588 (2021). 10.1021/acsptsci.0c00208 PMC8033600 33860188

[78] Gaul, S. et al. Novel nongenetic murine model of hyperglycemia and hyperlipidemia-associated aggravated atherosclerosis. *Front. Cardiovasc. Med.* **9**, 813215 (2022). 10.3389/fcvm.2022.813215 PMC8957812 35350534

[79] Holness, M. J. & Sugden, M. C. Pyruvate dehydrogenase activities during the fed-to-starved transition and on re-feeding after acute or prolonged starvation. *Biochem. J.* **258**, 529–533 (1989). 10.1042/bj2580529 PMC1138393 2705997

[80] Scherz-Shouval, R. et al. Reactive oxygen species are essential for autophagy and specifically regulate the activity of Atg4. *EMBO J.* **26**, 1749–1760 (2007). 10.1038/sj.emboj.7601623 PMC1847657 17347651

[81] Hulett, N. A., Scalzo, R. L., Reusch, J. E. B. Glucose uptake by skeletal muscle within the contexts of type 2 diabetes and exercise: an integrated approach. *Nutrients* **14**, 10.3390/nu14030647 (2022). PMC8839578 35277006

[82] Jeoung, N. H. et al. Role of pyruvate dehydrogenase kinase isoenzyme 4 (PDHK4) in glucose homoeostasis during starvation. *Biochem. J.* **397**, 417–425 (2006). 10.1042/BJ20060125 PMC1533314 16606348

[83] Kohda, Y. et al. Thiamine ameliorates diabetes-induced inhibition of pyruvate dehydrogenase (PDH) in rat heart mitochondria: investigating the discrepancy between PDH activity and PDH E1alpha phosphorylation in cardiac fibroblasts exposed to high glucose. *J. Pharm. Sci.* **113**, 343–352 (2010). 10.1254/jphs.09359fp 20644337

[84] Lydell, C. P. et al. Pyruvate dehydrogenase and the regulation of glucose oxidation in hypertrophied rat hearts. *Cardiovasc. Res.* **53**, 841–851 (2002). 10.1016/s0008-6363(01)00560-0 PMC2131743 11922894

[85] Bhatti, J. S. et al. Oxidative stress in the pathophysiology of type 2 diabetes and related complications: Current therapeutics strategies and future perspectives. *Free Radic. Biol. Med.* **184**, 114–134 (2022). 10.1016/j.freeradbiomed.2022.03.019 35398495

[86] Goodpaster, B. H. & Sparks, L. M. Metabolic flexibility in health and disease. *Cell Metab.* **25**, 1027–1036 (2017). 10.1016/j.cmet.2017.04.015 PMC5513193 28467922

[87] Park, J. M. et al. Hyperpolarized NMR study of the impact of pyruvate dehydrogenase kinase inhibition on the pyruvate dehydrogenase and TCA flux in type 2 diabetic rat muscle. *Pflugers Archiv**:**Eur. J. Physiol.* **473**, 1761–1773 (2021). 10.1007/s00424-021-02613-3 PMC9219576 34415396

[88] Pearson-Stuttard, J. et al. Type 2 diabetes and cancer: an umbrella review of observational and mendelian randomization studies. *Cancer Epidemiol. Biomark. Prev.* **30**, 1218–1228 (2021). 10.1158/1055-9965.EPI-20-1245 PMC9398112 33737302

[89] S3-Leitlinie: Chirurgie der Adipositas und metabolischer Erkrankungen. *AWMF Registry* (2018).

[90] Poljo, A. et al. The role of bariatric surgery on beta-cell function and insulin resistance in patients with nonalcoholic fatty liver disease and steatohepatitis. *Surg. Obes. Relat. Dis.* **19**, 1421–1434 (2023). 10.1016/j.soard.2023.07.005 37666725

[91] Bedossa, P. et al. Histopathological algorithm and scoring system for evaluation of liver lesions in morbidly obese patients. *Hepatology* **56**, 1751–1759 (2012). 10.1002/hep.25889 22707395

[92] Doench, J. G. et al. Optimized sgRNA design to maximize activity and minimize off-target effects of CRISPR-Cas9. *Nat. Biotechnol.* **34**, 184–191 (2016). 10.1038/nbt.3437 PMC4744125 26780180

[93] Woodard, L. E. & Wilson, M. H. piggyBac-ing models and new therapeutic strategies. *Trends Biotechnol.* **33**, 525–533 (2015). 10.1016/j.tibtech.2015.06.009 PMC4663986 26211958

[94] Chretien, D. et al. An improved spectrophotometric assay of pyruvate dehydrogenase in lactate dehydrogenase contaminated mitochondrial preparations from human skeletal muscle. *Clin. Chim. Acta* **240**, 129–136 (1995). 10.1016/0009-8981(95)06145-6 8548923

[95] Dagley, M. J. & McConville, M. J. DExSI: a new tool for the rapid quantitation of 13C-labelled metabolites detected by GC-MS. *Bioinformatics* **34**, 1957–1958 (2018). 10.1093/bioinformatics/bty025 PMC5972663 29360933

[96] Percher, A., Thinon, E. & Hang, H. Mass-tag labeling using acyl-PEG exchange for the determination of endogenous protein S-fatty acylation. *Curr. Protoc. Protein Sci.* **89**, 14 17 11-14 17 11 (2017). 10.1002/cpps.36 PMC5645067 28762493

[97] Rabbani, N. & Thornalley, P. J. Measurement of methylglyoxal by stable isotopic dilution analysis LC-MS/MS with corroborative prediction in physiological samples. *Nat. Protoc.* **9**, 1969–1979 (2014). 10.1038/nprot.2014.129 25058644

