# Supplementary material for: Regulation of PDH via cysteine oxidation on DLAT
Source: Nat Commun. 2026 Oct 14;17:10784. doi: 10.1038/s41467-026-78266-6 (PMC13669365; doi:10.1038/s41467-026-78266-6)

Supplementary Information

Regulation of PDH via cysteine oxidation on DLAT

Fabiola Garcia-Cortizo, Tobias Weber, Hana Nůsková, Daniel Pfaff, Michael Büttner, Angela Wirth, Elisabeth Kliemank, Gernot Poschet, Marc Freichel, Thomas Fleming, Alba Sulaj, Peter Paul Nawroth, Julia Szendroedi and Aurelio A. Teleman

Contents

Supplementary Figures .... p. 3

Uncropped scans of all blots in Supplementary Figures .... p. 23

Supplementary Figure 1

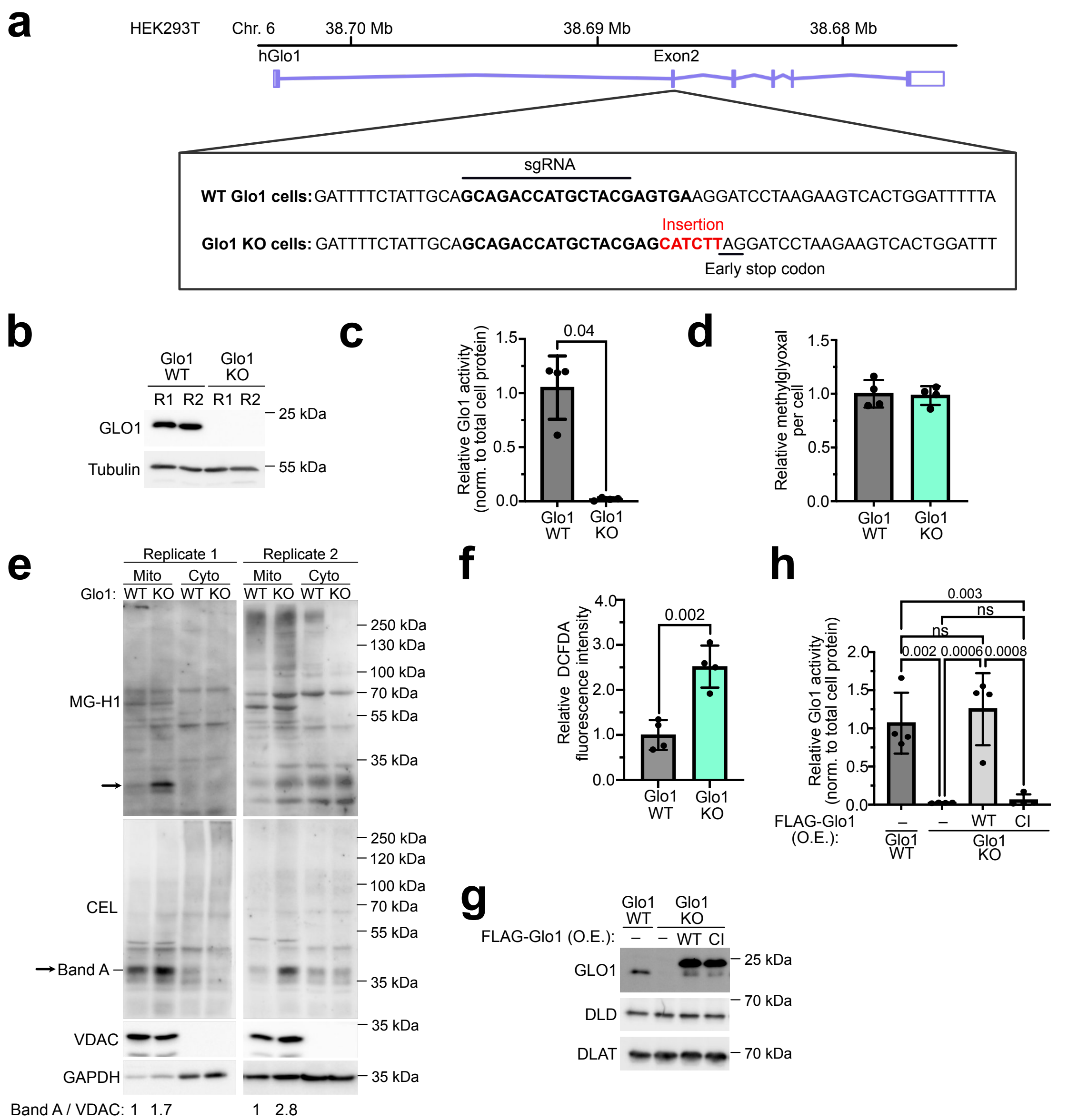

Supplementary Figure 1: Characterization of Glo1^{KO} HEK293T cells

(a) Molecular characterization of the GLO1 knockout (GLO1^{KO}) HEK293T cells generated using CRISPR/Cas9.

(b) GLO1^{KO} HEK293T cells have no detectable GLO1 protein. n=2 biological replicates.

(c) GLO1^{KO} HEK293T cells have no detectable GLO1 activity. n=4 biological replicates, significance by two-tailed unpaired t-test.

(d) Free intracellular MG is not increased in $GLO1^{KO}$ cells. MG levels in WT or $GLO1^{KO}$ cells were measured by derivatization and mass spectrometry. n=5 biological replicates.
(e) HEK293T $GLO1^{KO}$ cells have a mild increase in MG adducts (MG-H1 on arginines and CEL on lysines) in mitochondria. n=3 biological replicates.
(f) $GLO1^{KO}$ HEK293T cells have elevated ROS levels. n=4 biological replicates, significance by two-tailed unpaired t-test.
(g) Control for expression of FLAG-tagged wildtype (WT) or catalytically inactive (CI) GLO1 protein in $GLO1^{KO}$ HEK293T cells. n=2 biological replicates.
(h) Stable transfection of WT GLO1 but not catalytically inactive (CI) GLO1 restores GLO1 activity in $GLO1^{KO}$ HEK293T cells. n=4 biological replicates, significance by one way ANOVA corrected for multiple comparisons with Tukey test.
All data are presented as mean values +/- Std. Dev. Source data are provided as a Source Data file.

(continued on next page)

Supplementary Figure 2

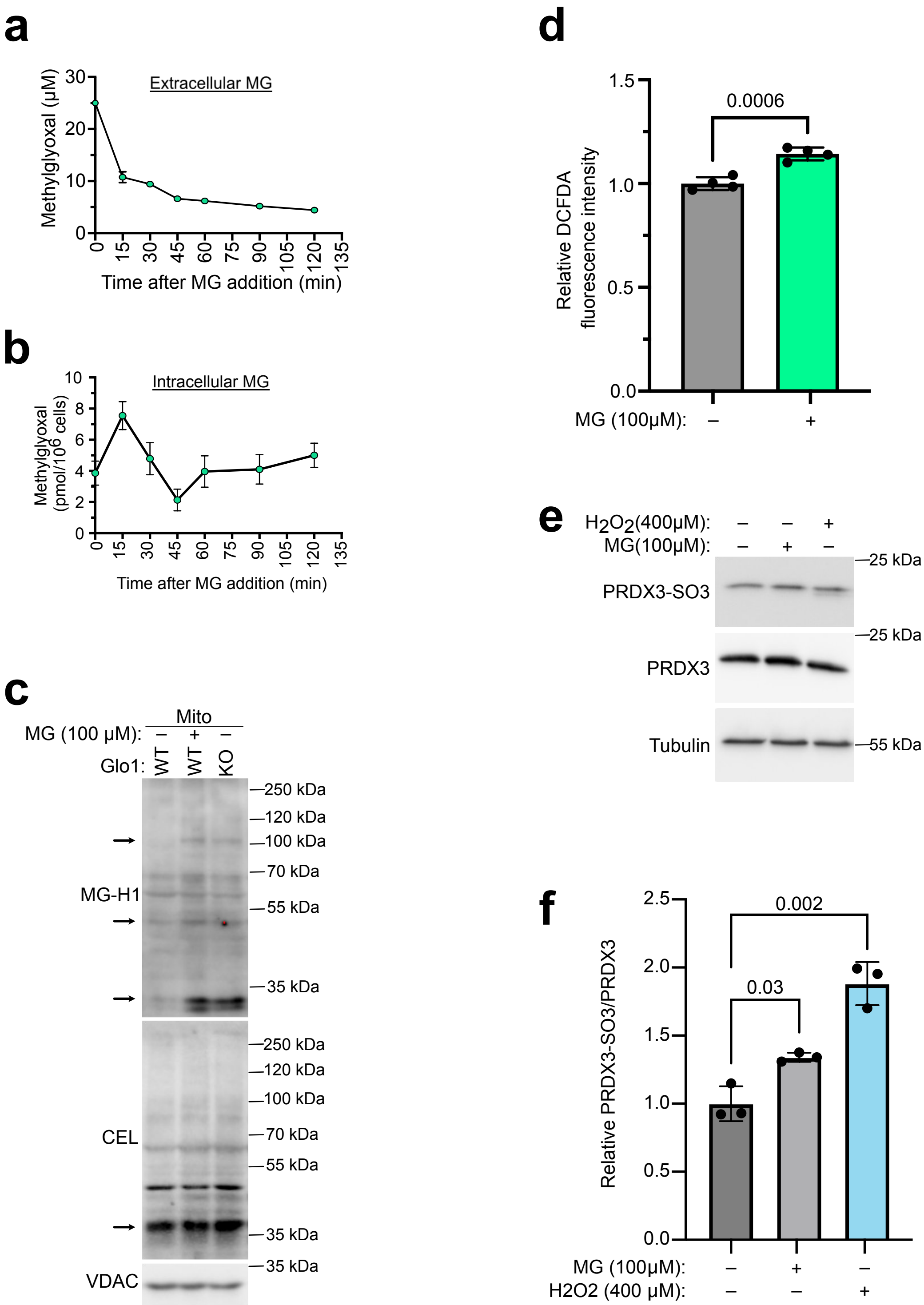

Supplementary Figure 2: Support to main Figure 1.

(a-b) Methylglyoxal (MG) added to the medium of HEK293T cells at 25 µM concentration is rapidly detoxified by the cells (a), causing intracellular MG

levels to increase transiently, peaking 15 minutes after addition (b). n=3 biological replicates.

(c) HEK293T cells incubated with 100 µM MG for 1 h show a similar increase in MG adducts (MG-H1 and CEL) in their mitochondria as HEK293T GLO1^{KO} cells.

(d) Treatment of HEK293T cells with MG leads to elevated ROS levels, quantified with the dye DCFDA. n=4 biological replicates, significance by two-tailed unpaired t-test.

(e-f) Both H_2O_2 and MG increase hyperoxidation of PRDX3, assayed via an antibody that detects sulfonylated PRDX3 (SO_3-PRDX3). (e) Representative western blot. (f) Quantification of 3 biological replicates. Significance by one way ANOVA corrected for multiple comparisons with Šídák test.

All data are presented as mean values +/- Std. Dev. Source data are provided as a Source Data file.

(continued on next page)

Supplementary Figure 3

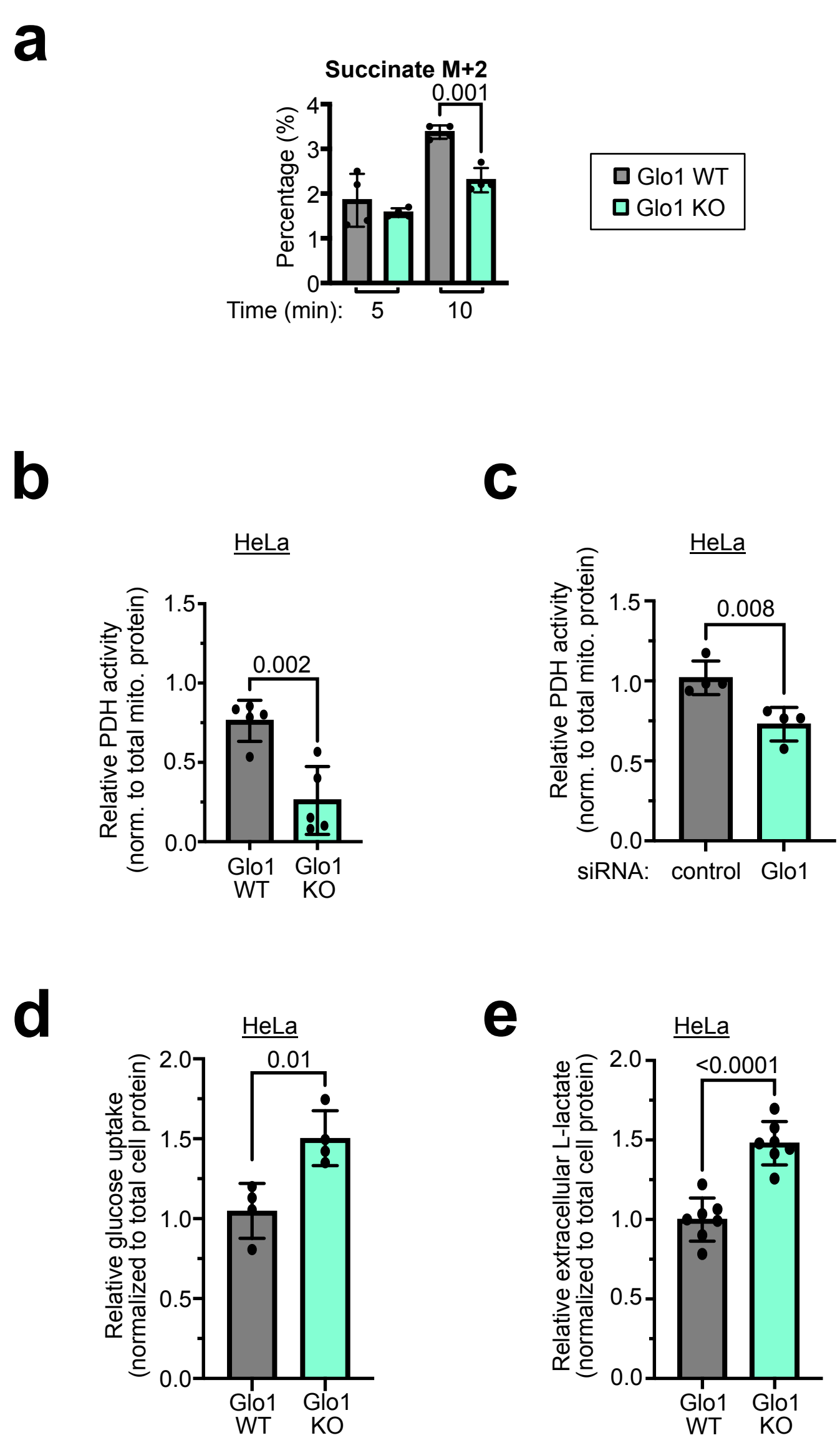

Supplementary Figure 3: Glo1^{KO} cells have reduced PDH activity and reduced glycolytic flux into the TCA cycle.

(a) GLO1^{KO} HEK293T cells have reduced carbon flux from glycolysis into the TCA cycle. Shown is the percent labeling with 13C carbon after incubation for 5 or 10 minutes with 13C glucose. n=4 biological replicates, significance by one way ANOVA corrected for multiple comparisons with Šídák test.

(b) PDH activity is decreased in GLO1^{KO} HeLa cells. n=5 biological replicates, significance by two-tailed unpaired t-test.

(c) PDH activity is decreased in GLO1 knockdown HeLa cells. siRNA targeting Renilla luciferase used as negative control. n=4 biological replicates, significance by two-tailed unpaired t-test.

(d) $GLO1^{KO}$ HeLa cells have higher glucose uptake, measured as the drop in glucose in the medium over a 24 hour period, normalized to cellular protein. n=4 biological replicates, significance by two-tailed unpaired t-test.

(e) $GLO1^{KO}$ HeLa cells produce more L-lactate, measured as the increase in lactate in medium over a 24 hour period. n=7 biological replicates, significance by two-tailed unpaired t-test.

All data are presented as mean values +/- Std. Dev. Source data are provided as a Source Data file.

(continued on next page)

Supplementary Figure 4

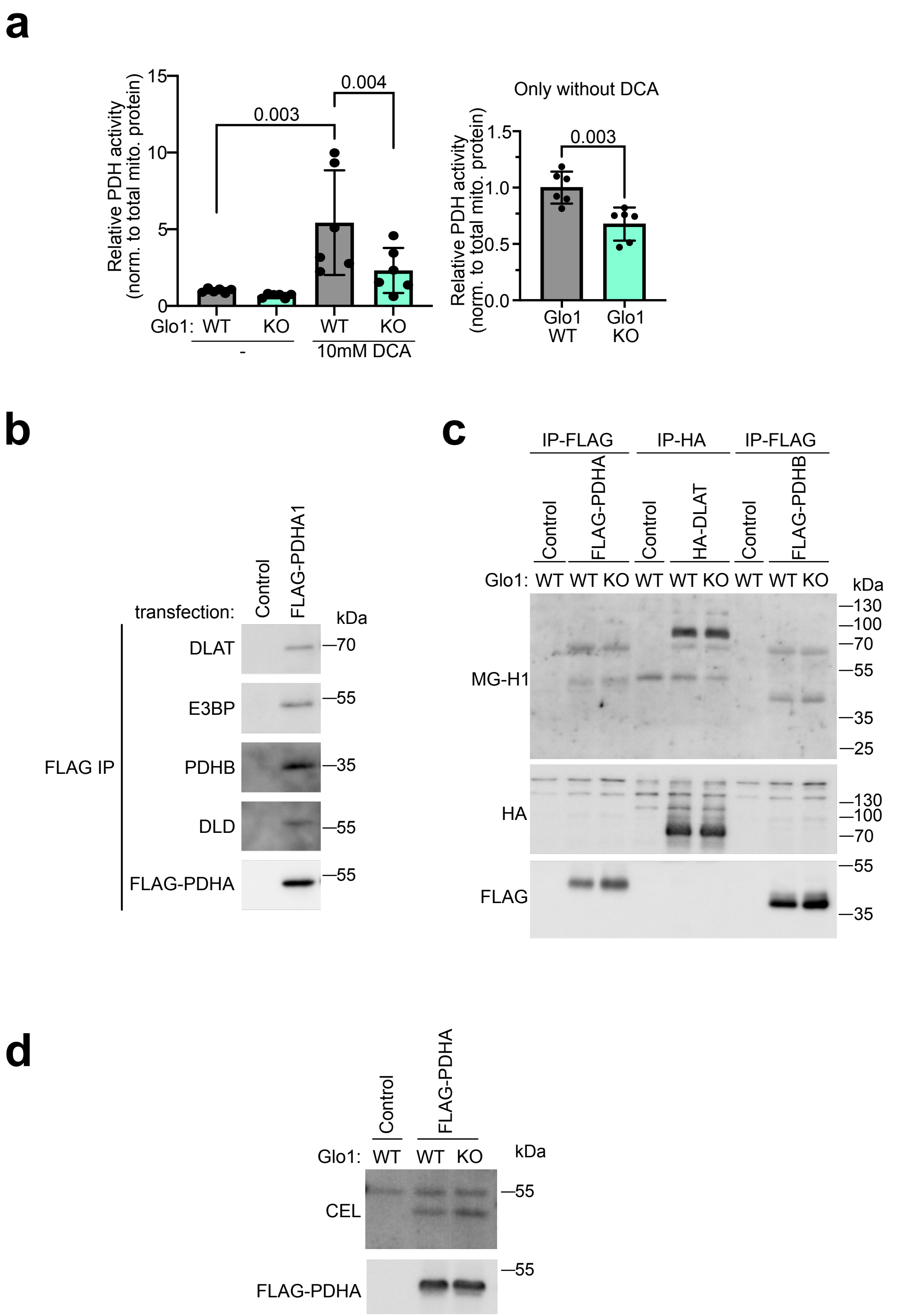

Supplementary Figure 4: MG adducts on PDH subunits.

(a) GLO1^{KO} HEK293T cells have reduced PDH activity compared to GLO1^{WT} cells also in the presence of dichloroacetate (DCA), an inhibitor of PDK and hence PDH phosphorylation. n=6 biological replicates, (left panel) significance

by one way ANOVA corrected for multiple comparisons with Tukey test or (right panel) significance by two-tailed unpaired t-test.

(b) All PDH subunits are present in a FLAG-PDHA immunoprecipitation (IP) from HEK293T cells. n=2 biological replicates.

(c) There is no detectable increase in the level of MG-H1 adducts on PDH subunits PDHA, DLAT and PDB in $GLO1^{KO}$ HEK293T cells. Subunits were immunoprecipitated via their epitope tags, and then immunoblotted with anti-MG-H1 antibody.

(d) CEL modification of PDHA is mildly elevated in $GLO1^{KO}$ HEK293T cells in this one replicate shown, out of three replicates. In the other two replicates, CEL levels were equal in $GLO1^{WT}$ and $GLO1^{KO}$ cells. PDHA is immunoprecipitated via its FLAG tag and subjected to immunoblotting with anti-CEL antibody. n=3 biological replicates.

All data are presented as mean values +/- Std. Dev. Source data are provided as a Source Data file.

(continued on next page)

Supplementary Figure 5

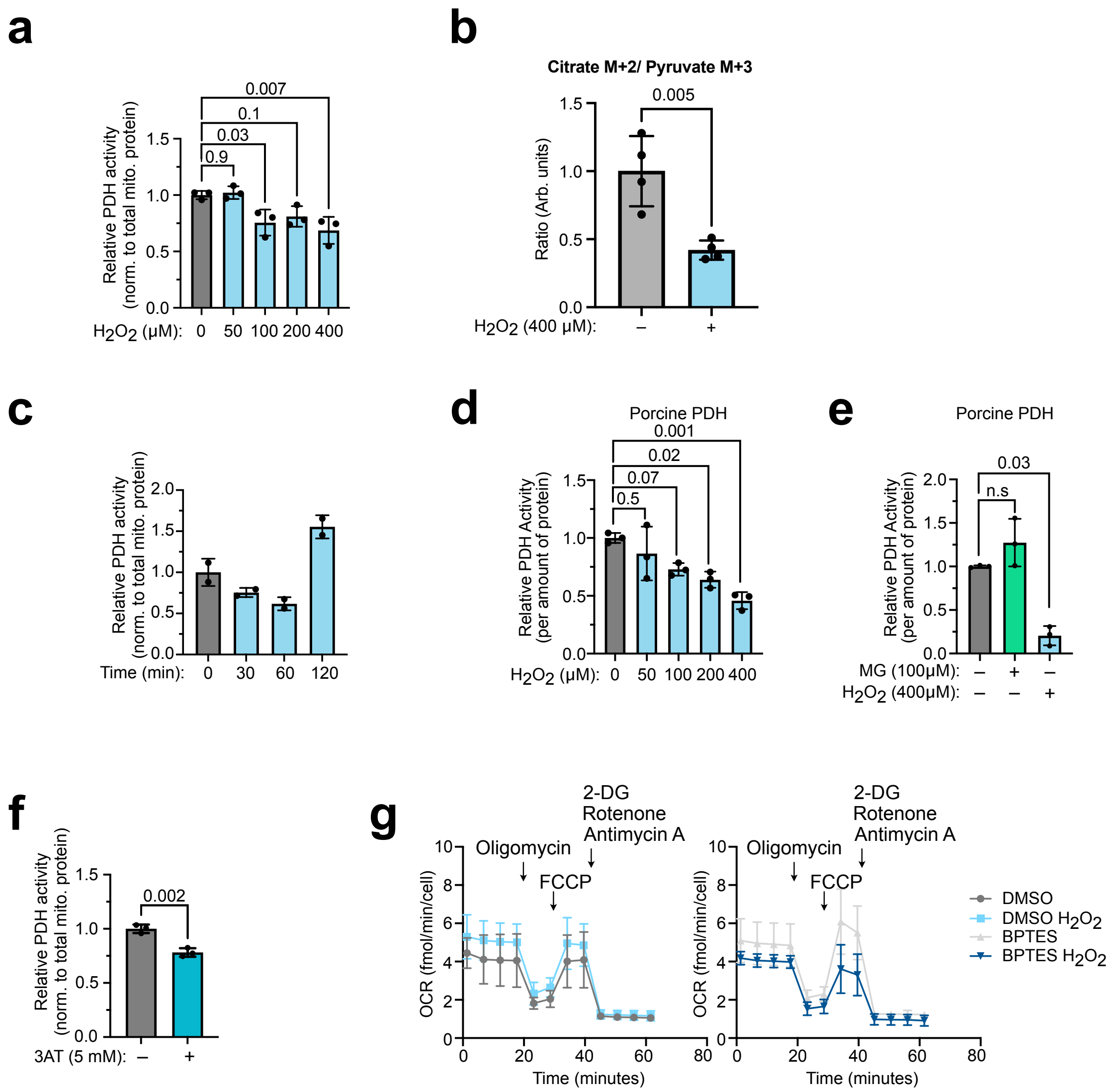

Supplementary Figure 5: H_2O_2 inhibits PDH activity

(a) Dose-response curve, treating HEK293T cells with H_2O_2 and measuring PDH activity reveals that 100µM H_2O_2 is sufficient to inhibit PDH. n=3 biological replicates, significance by one way ANOVA corrected for multiple comparisons with Šídák test.

(b) Treatment with H_2O_2 reduces carbon flux from glycolysis into the TCA cycle. Shown is the ratio in percent labeling with 13C carbon of citrate to pyruvate after incubation for 10 minutes with 13C glucose. n=4 biological replicates, significance by two-tailed unpaired t-test.

(c) Inhibition of PDH by H_2O_2 is reversible. HEK293T cells were treated with 400μM H_2O_2 and PDH activity was then measured at the indicated timepoints. n=2 biological replicates.

(d) Activity of purified porcine PDH is reduced upon treatment in vitro with the indicated concentrations of H_2O_2 for 1 hour at 37°C, indicating it is a direct effect. n=3 biological replicates. Significance by one way ANOVA corrected for multiple comparisons with Šídák test.

(e) The activity of purified porcine PDH is reduced upon treatment in vitro with H_2O_2 but not MG for 1 hour at 37°C. n=3 biological replicates. Significance by one way ANOVA corrected for multiple comparisons with Šídák test.

(f) PDH activity is impaired in HEK293T cells incubated with a catalase inhibitor, 3-amino-1,2,4-triazole (3AT), for 6h. n=3 biological replicates, significance by two-tailed unpaired t-test.

(g) The increase in respiration observed upon treatment of HEK293T cells with H_2O_2 results from increased glutaminolysis. Cells were treated with or without 200μM H_2O_2 in combination with 3μM BPTES or DMSO as a negative control for 1 hour, and then oxygen consumption was measured using the SeaHorse Mito Stress Test Kit. Oxygen consumption is normalized per cell. n=3 biological replicates on different days with multiple wells each. All cells were assayed together on one plate, but results are shown in two separate graphs for visualization to avoid overlap. In the presence of the glutaminolysis inhibitor BPTES, H_2O_2 causes a drop in respiration (right panel), as would be expected from a decrease in PDH activity.

All data are presented as mean values +/- Std. Dev. Source data are provided as a Source Data file.

(continued on next page)

Supplementary Figure 6

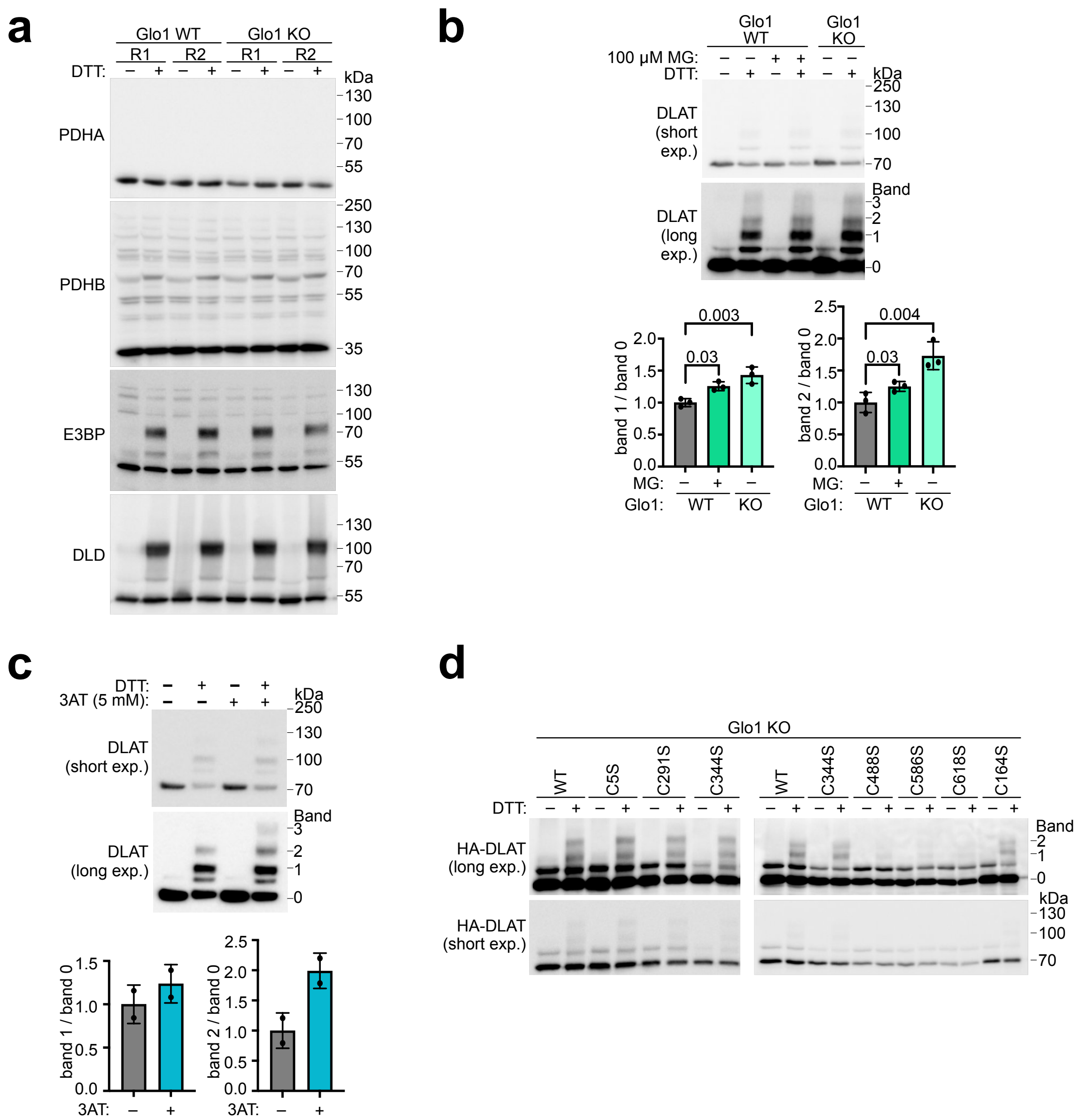

Supplementary Figure 6: DLAT, but not other subunits of PDH, is oxidized in GLO1^{KO} HEK293T cells.

(a) There is no detectable increase in oxidation of PDHA, PDHB, E3BP or DLD in GLO1^{KO} HEK293T cells, assayed by PEG exchange (see Fig. 2f). n=2 biological replicates.

(b) Oxidation of DLAT increases in HEK293T cells treated with methylglyoxal (MG) (100μM, 1h). Representative blot (top) of 3 biological replicates, quantified below, significance by one way ANOVA corrected for multiple comparisons with Tukey test.

(c) PDH oxidation is increased in $GLO1^{WT}$ HEK293T cells treated with a catalase inhibitor 3AT for 6 hours. Representative blot (top) of 2 biological replicates, quantified below.

(d) Mutagenesis screen reveals that oxidation of DLAT is reduced upon mutation of cysteine 488, 586 or 618. n=2 biological replicates.

All data are presented as mean values +/- Std. Dev. Source data are provided as a Source Data file.

(continued on next page)

Supplementary Figure 7

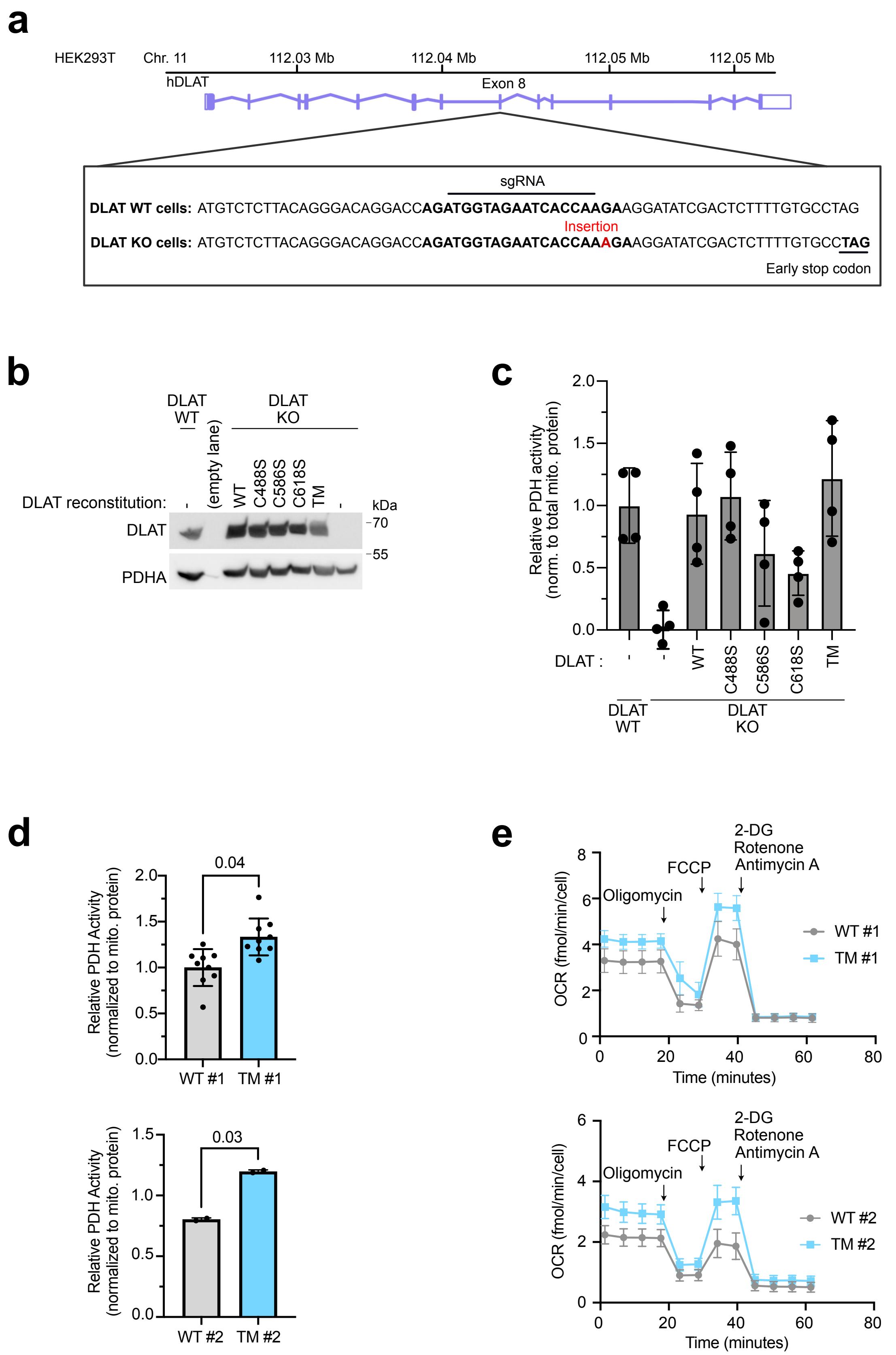

Supplementary Figure 7: Triple-mutant C488S/C585S/C618S DLAT has elevated activity

(a) Molecular characterization of the $DLAT^{KO}$ knockout cell line identifies a homozygous frame-shift mutation that causes a premature stop codon in the coding sequence.

(b) DLAT protein levels in control cells, $DLAT^{KO}$ cells, and DLAT knockout cells stably transfected to express either wildtype DLAT or mutant DLAT. (TM = triple mutant). n=3 biological replicates.

(c) PDH activity in control cells, $DLAT^{KO}$ cells, and $DLAT^{KO}$ cells stably transfected to express either wildtype DLAT or mutant DLAT. (TM = triple mutant). n=4 biological replicates.

(d) Triple mutant DLAT enables higher PDH activity than wildtype DLAT. PDH activity was measured in DLAT knockout cells stably expressing either wildtype DLAT (WT) or C488S/C586S/C608S triple mutant (TM) DLAT as shown in panel B. Two separate sets of cells were generated (#1 and #2). The western blot in panel B corresponds to set #1. n=8 biological replicates for #1 and n=2 for #2. Significance by two-tailed paired t-test.

(e) Cells expressing C488S/C586S/C608S triple mutant (TM) DLAT have higher respiration than cells expressing wildtype DLAT, consistent with the elevated PDH activity in these cells (panel D). Respiration was assayed with a SeaHorse Mito Stress Test Kit. Oxygen consumption is normalized per cell. n=3 biological replicates on different days with multiple wells each.

All data are presented as mean values +/- Std. Dev. Source data are provided as a Source Data file.

(continued on next page)

Supplementary Figure 8

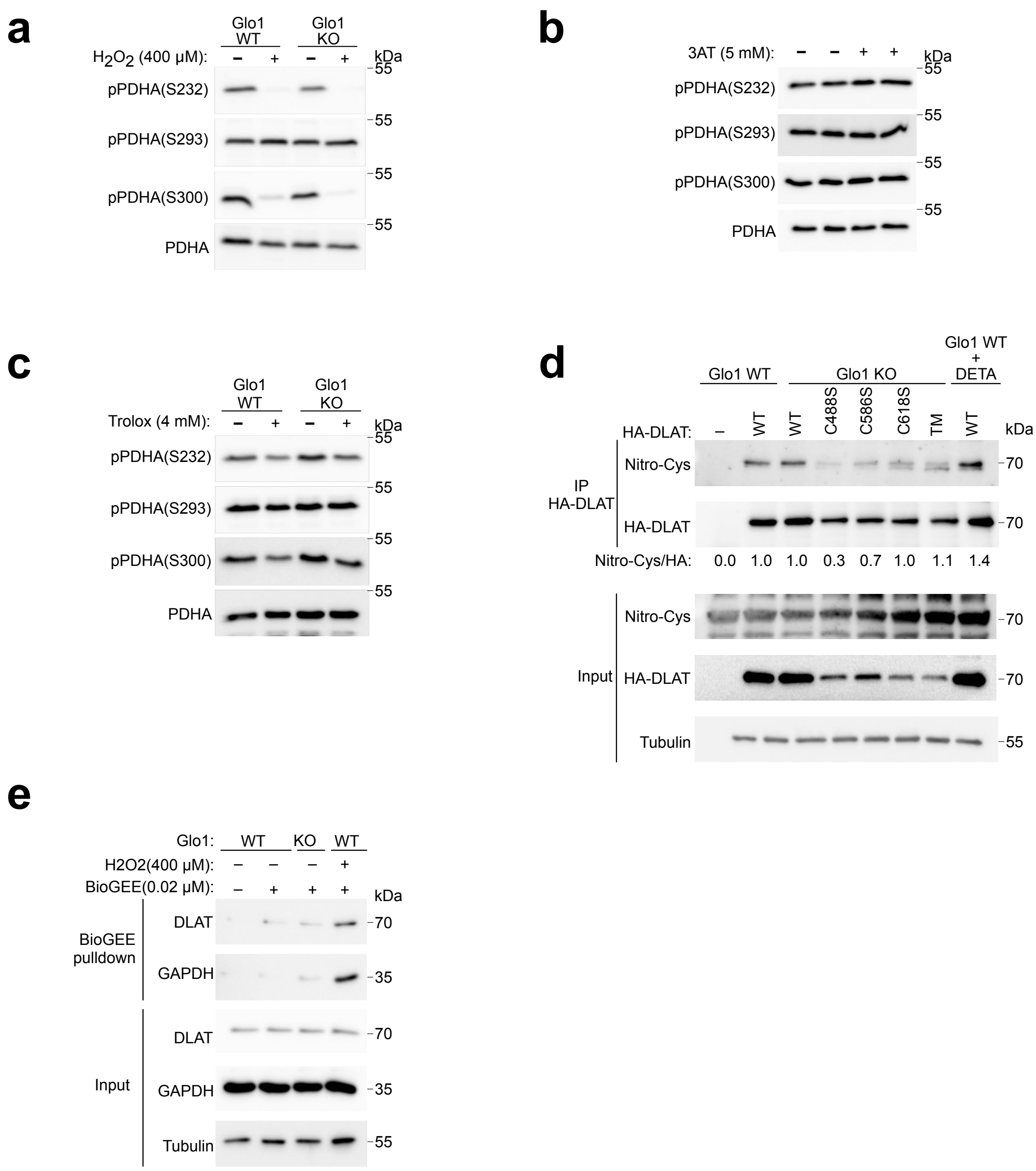

Supplementary Figure 8: DLAT is oxidized, but not differentially glutathionylated or nitrosylated, in GLO1^{KO} HEK293T cells.

(a) Phosphorylation of PDHA is decreased in WT and GLO1^{KO} HEK293T cells incubated with 400uM H_2O_2 for 1 hour. Representative of 2 biological replicates

(b) Phosphorylation of PDHA does not change in WT HEK293T cells upon catalase inhibition with 3AT for 6 hours. n=3 biological replicates.

(c) PDH phosphorylation is decreased in WT and GLO1^{KO} HEK293T cells incubated for 1 hour with 4mM trolox. Representative of 2 biological replicates.
(d) DLAT is not differentially nitrosylated in GLO1^{KO} compared to GLO1^{WT} HEK293T cells. Assessed by IP with HA-DLAT and WB. Biological replicates n=2.
(e) DLAT is not more gluthathionylated in GLO1^{KO} compared to GLO1^{WT} HEK293T cells. Assessed by pull down of biotinylated glutathione ethyl ester (BioGEE). n=2 biological replicates.

(continued on next page)

Supplementary Figure 9

a

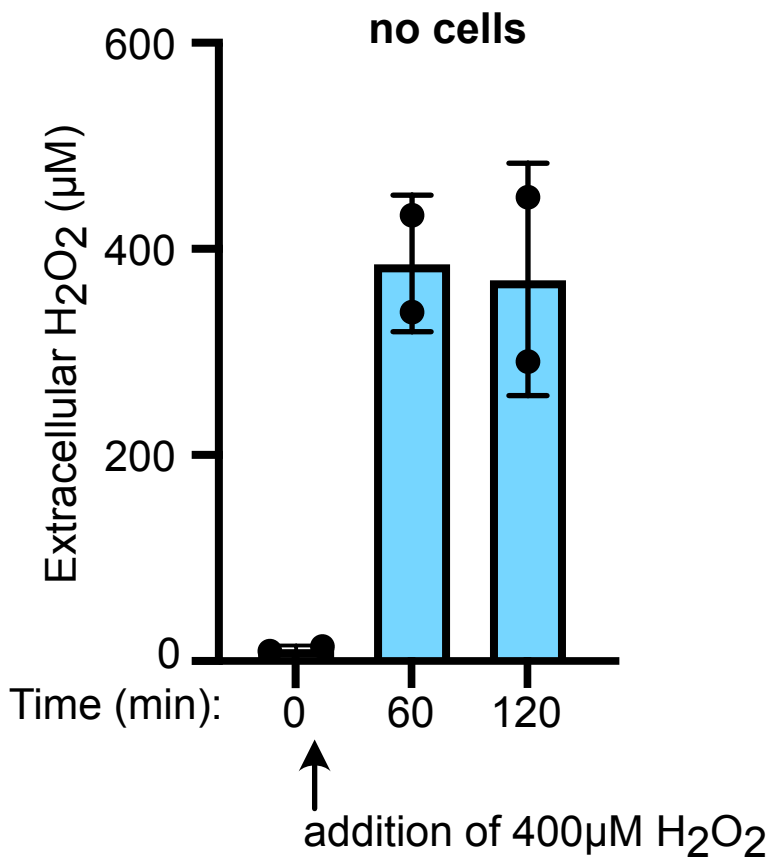

b

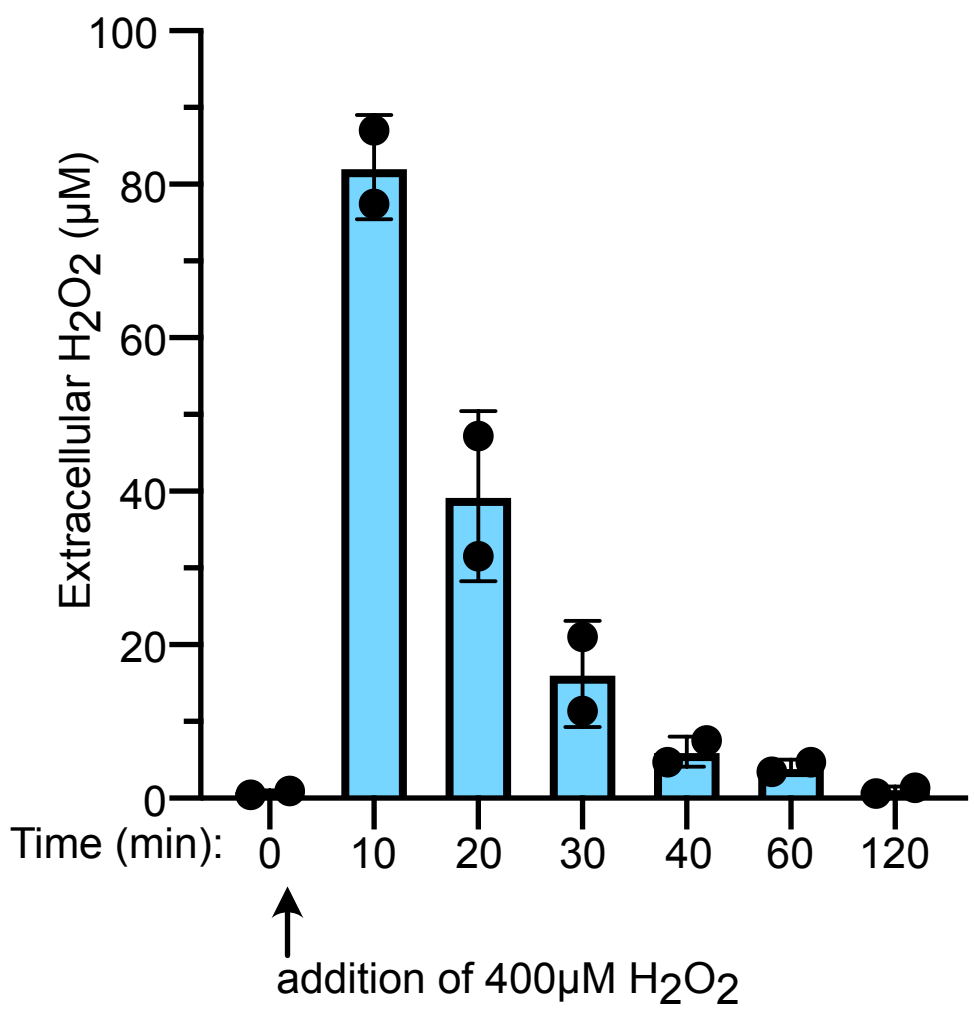

Supplementary Figure 9: Setup of the H_2O_2 detoxification assay

(a) H_2O_2 levels are stable in DMEM medium in the absence of cells for roughly 2 hours. 400µM H_2O_2 was added to DMEM medium in wells lacking cells, and H_2O_2 levels were then measured in the medium at 60 and 120 minutes after addition. n=2 biological replicates.

(b) H_2O_2 is rapidly detoxified by cells. 400µM H_2O_2 was added to wells containing cells, and then H_2O_2 levels in the medium were quantified at the indicated timepoints. n=2 biological replicates.

All data are presented as mean values +/- Std. Dev. Source data are provided as a Source Data file.

(continued on next page)

Supplementary Figure 10

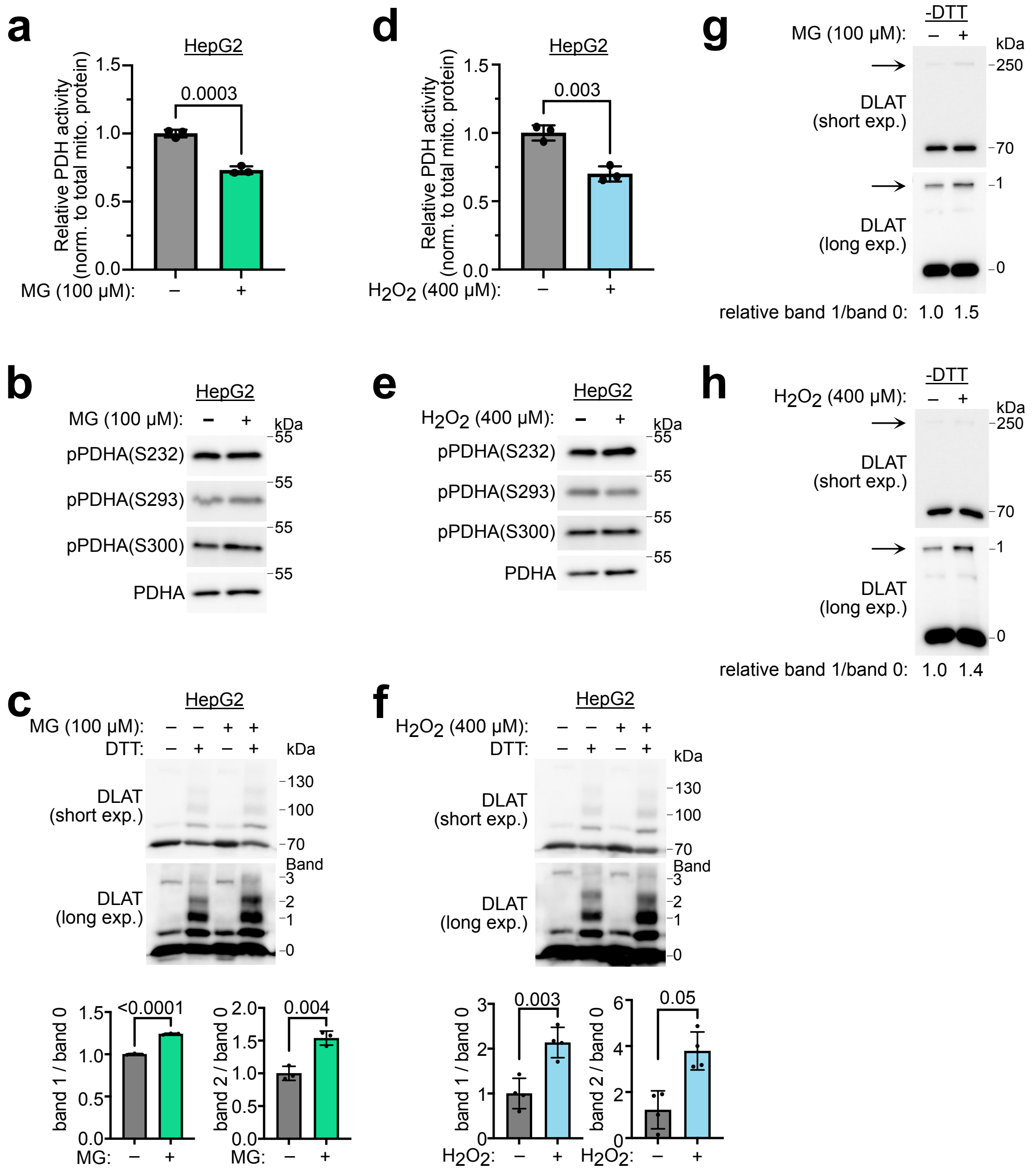

Supplementary Figure 10: Methylglyoxal and oxidative stress reduce PDH activity in HepG2 cells.

(a) PDH activity is inhibited in HepG2 cells incubated with 100µM methylglyoxal (MG) for 1 hour. n=3 biological replicates. Significance by two-tailed unpaired t-test.

(b) PDH phosphorylation is not elevated in HepG2 cells incubated with 100µM MG for 1 hour. n=3 biological replicates.

(c) Oxidation of DLAT increases in HepG2 cells treated for 1 hour with 100μM MG. Representative blot (top) of 3 biological replicates, quantified below. Significance by two-tailed unpaired t-test.
(d) PDH activity is inhibited in HepG2 cells incubated with 400μM H_2O_2 for 1 hour. n=3 biological replicates. Significance by two-tailed unpaired t-test.
(e) PDH phosphorylation on S232 is increased in HepG2 cells incubated with 400μM H_2O_2 for 1 hour.
(f) Oxidation of DLAT increases in HepG2 cells treated for 1 hour with 400μM H_2O_2. Representative blot (top) of 4 biological replicates, quantified below. Significance by two-tailed unpaired t-test.
(g) Incubation of HepG2 cells with 100μM MG for 1 hour increases levels of the high molecular weight band of DLAT. n=2 biological replicates.
(h) Incubation of HepG2 cells with 400μM H_2O_2 for 1 hour increases levels of the high molecular weight band of DLAT. n=2 biological replicates.
All data are presented as mean values +/- Std. Dev. Source data are provided as a Source Data file.

(continued on next page)

<u>Uncropped scans of all blots in Supplementary Figures</u>

(start on next page)

Supplementary Figure 1

B

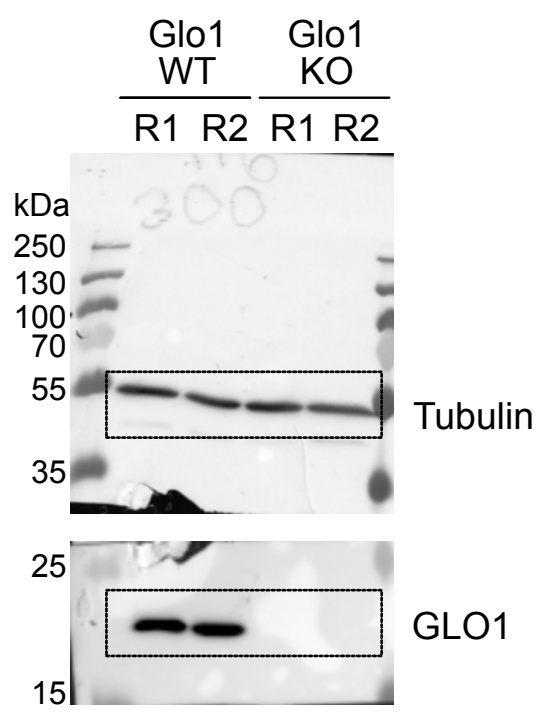

E

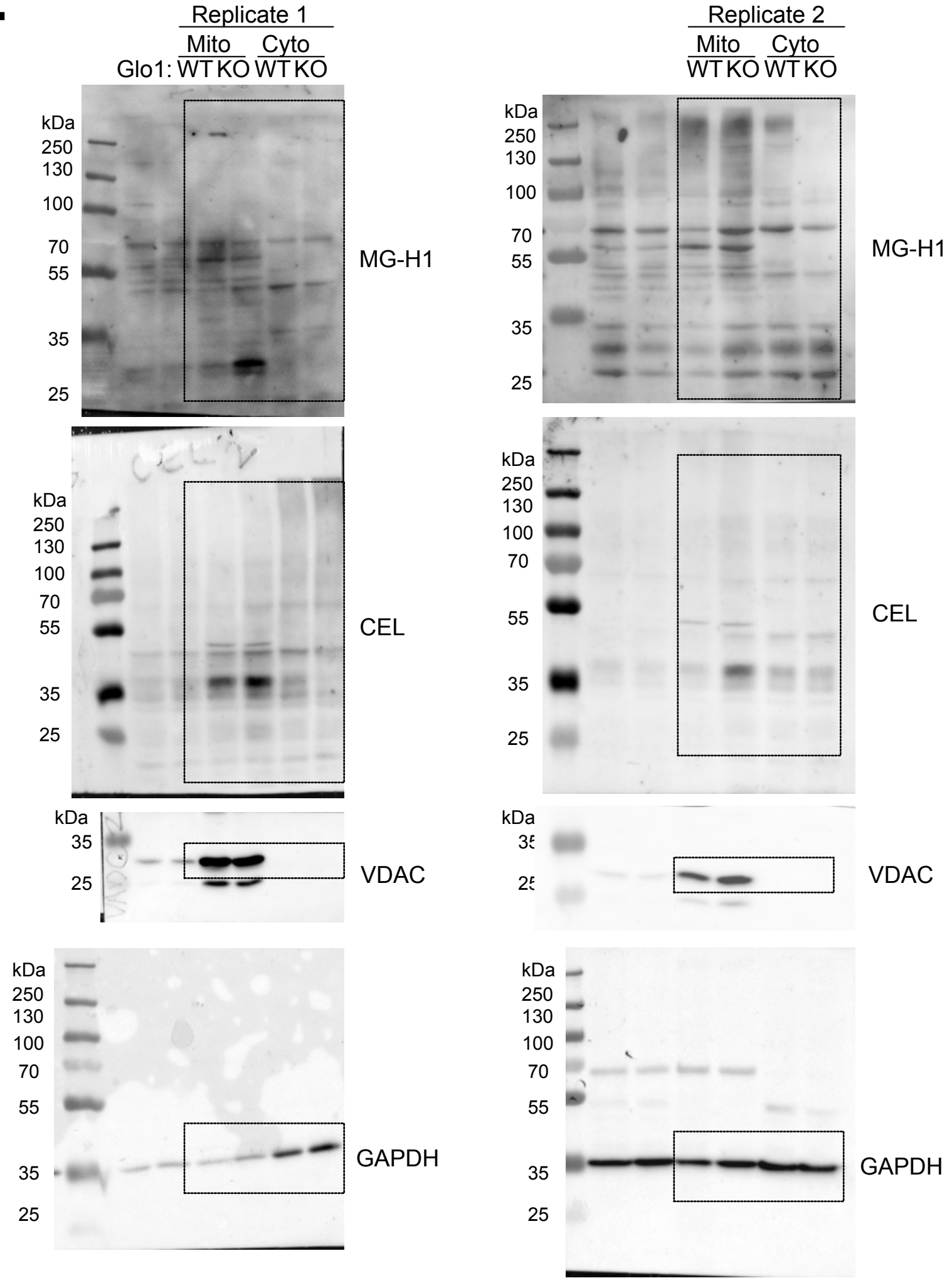

G

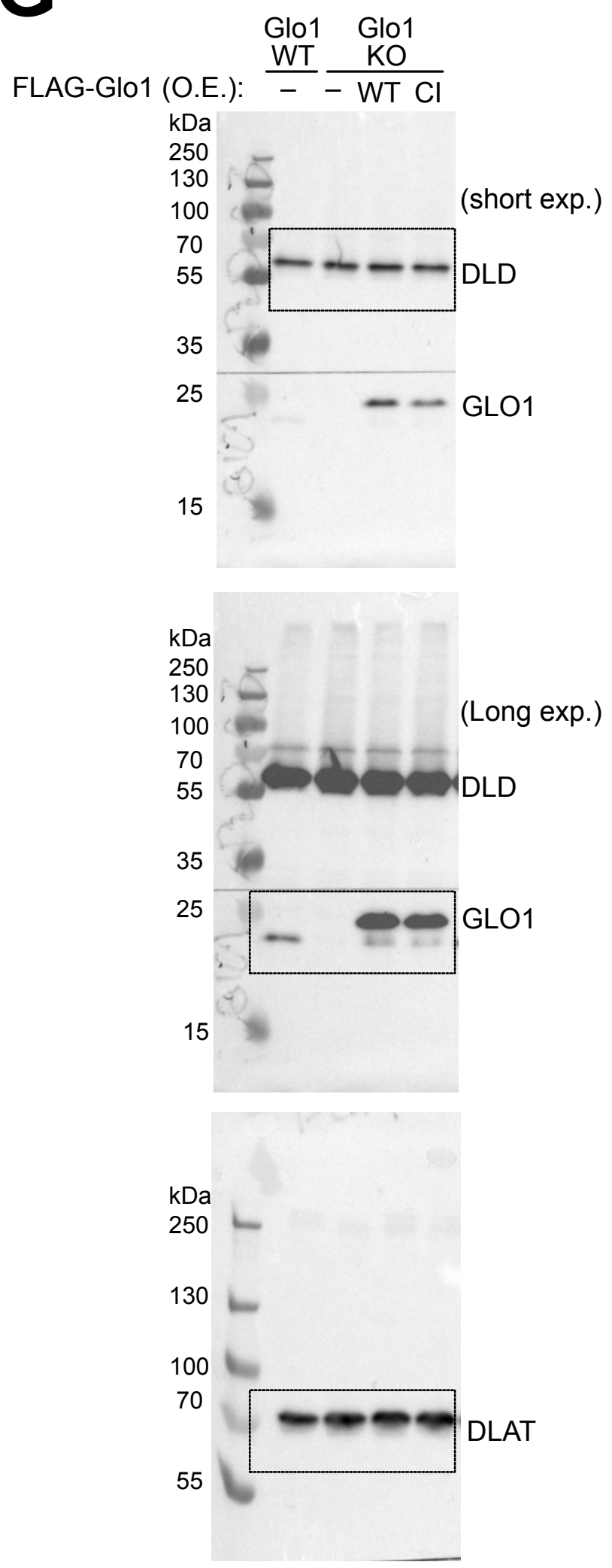

Supplementary Figure 2

C

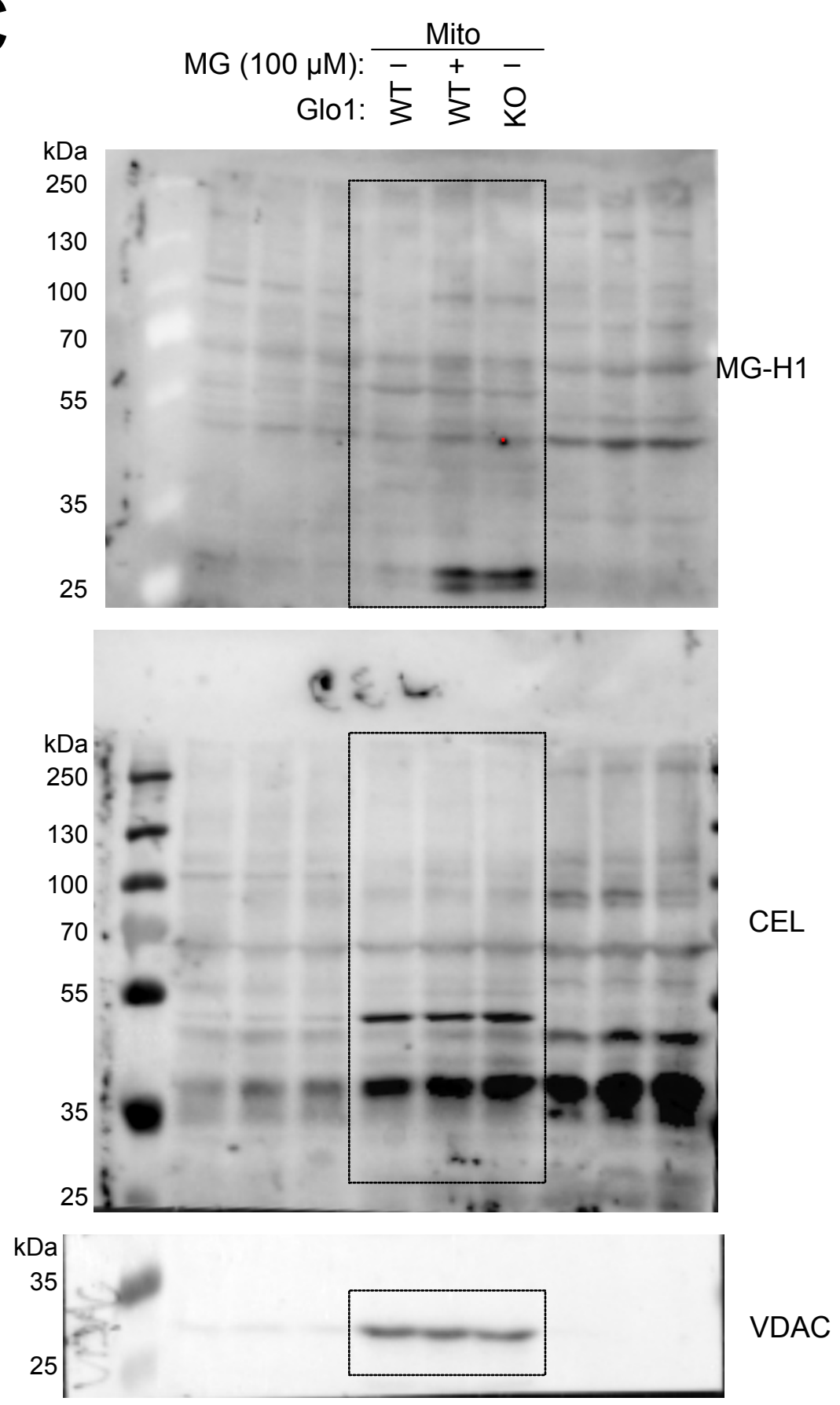

E

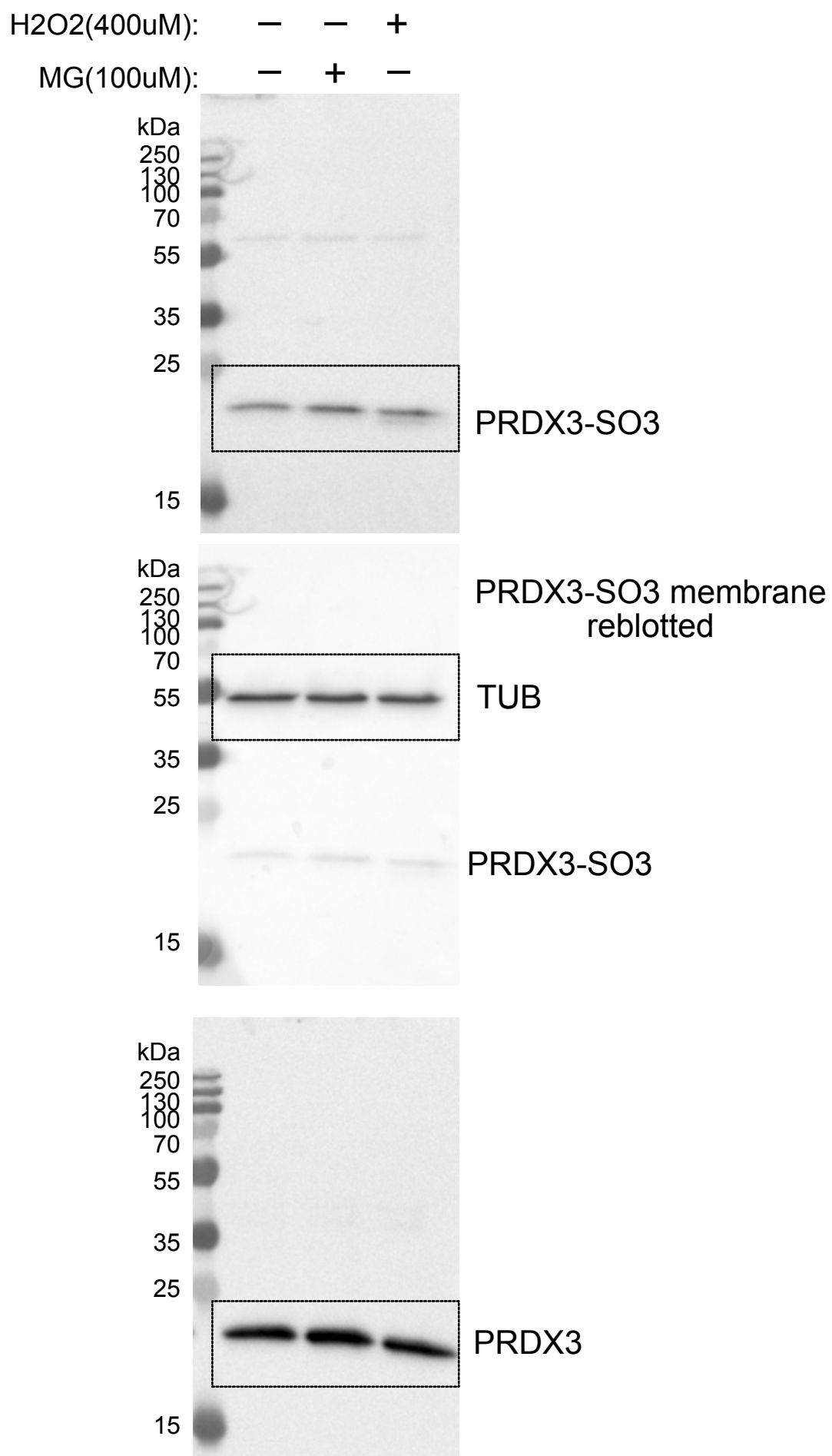

Supplementary Figure 4

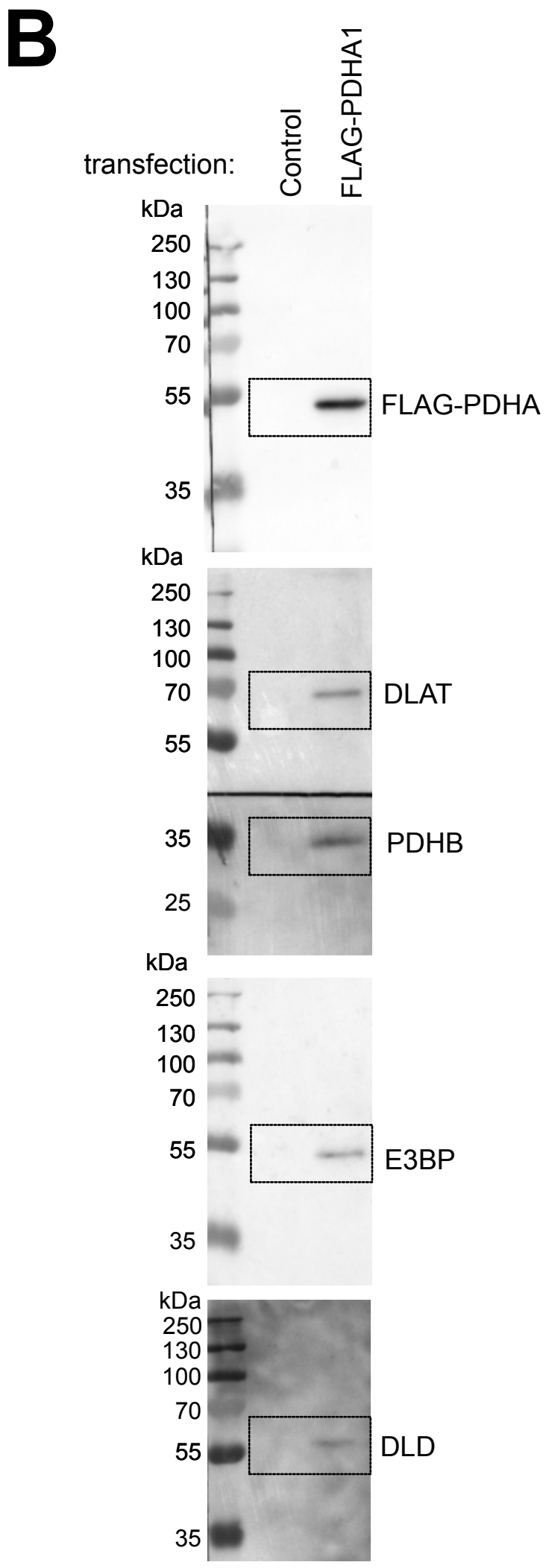

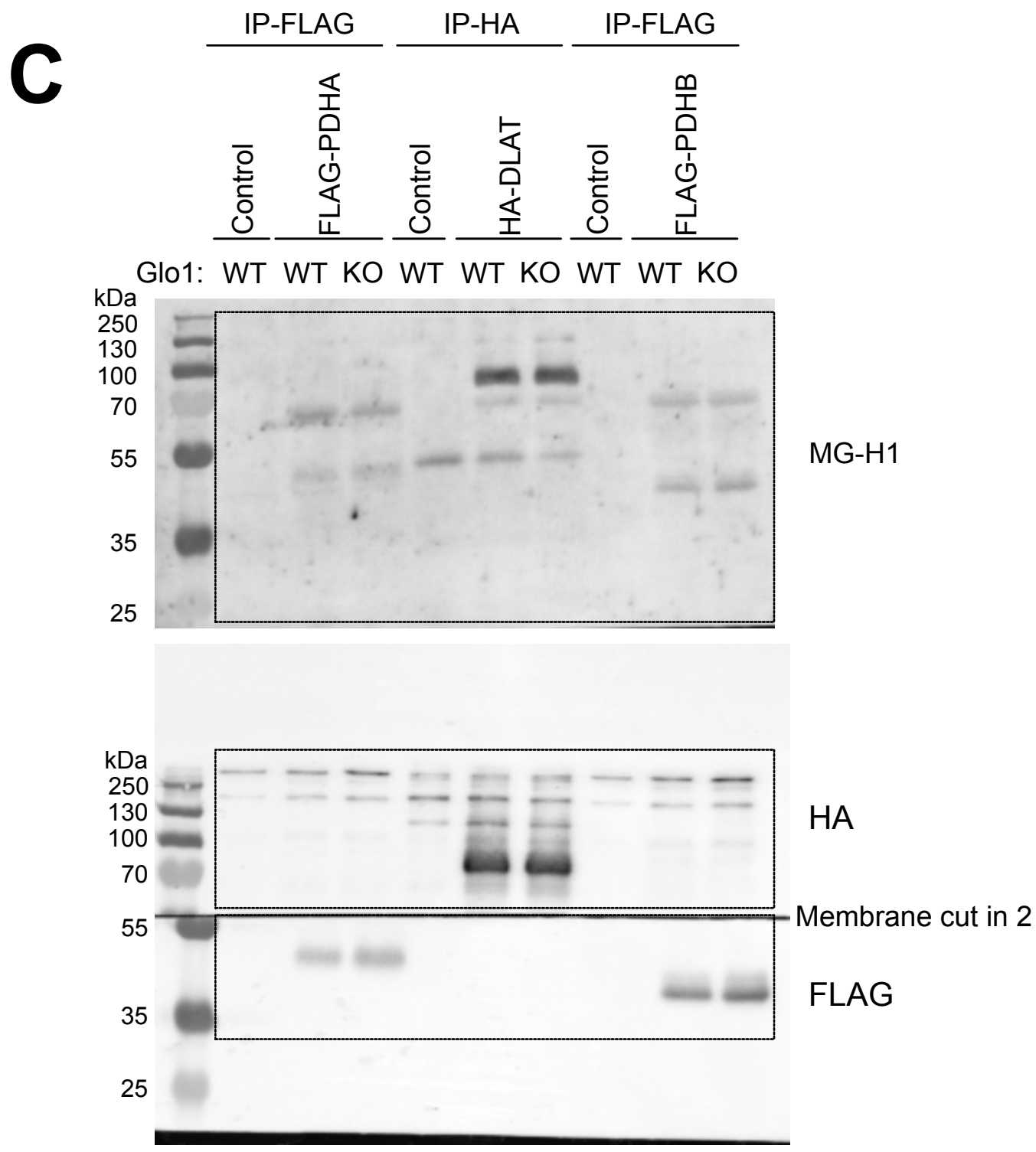

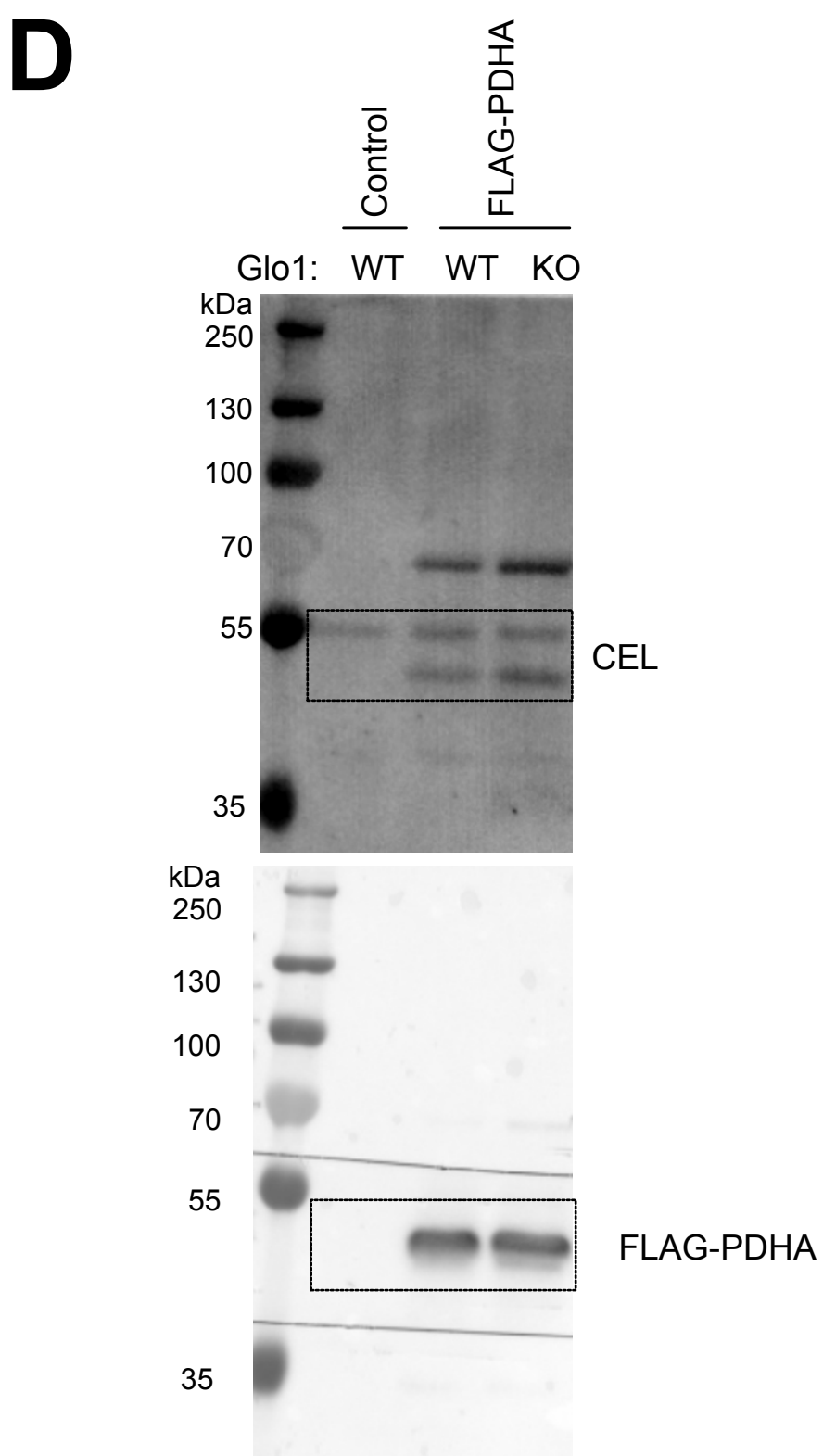

Supplementary Figure 6

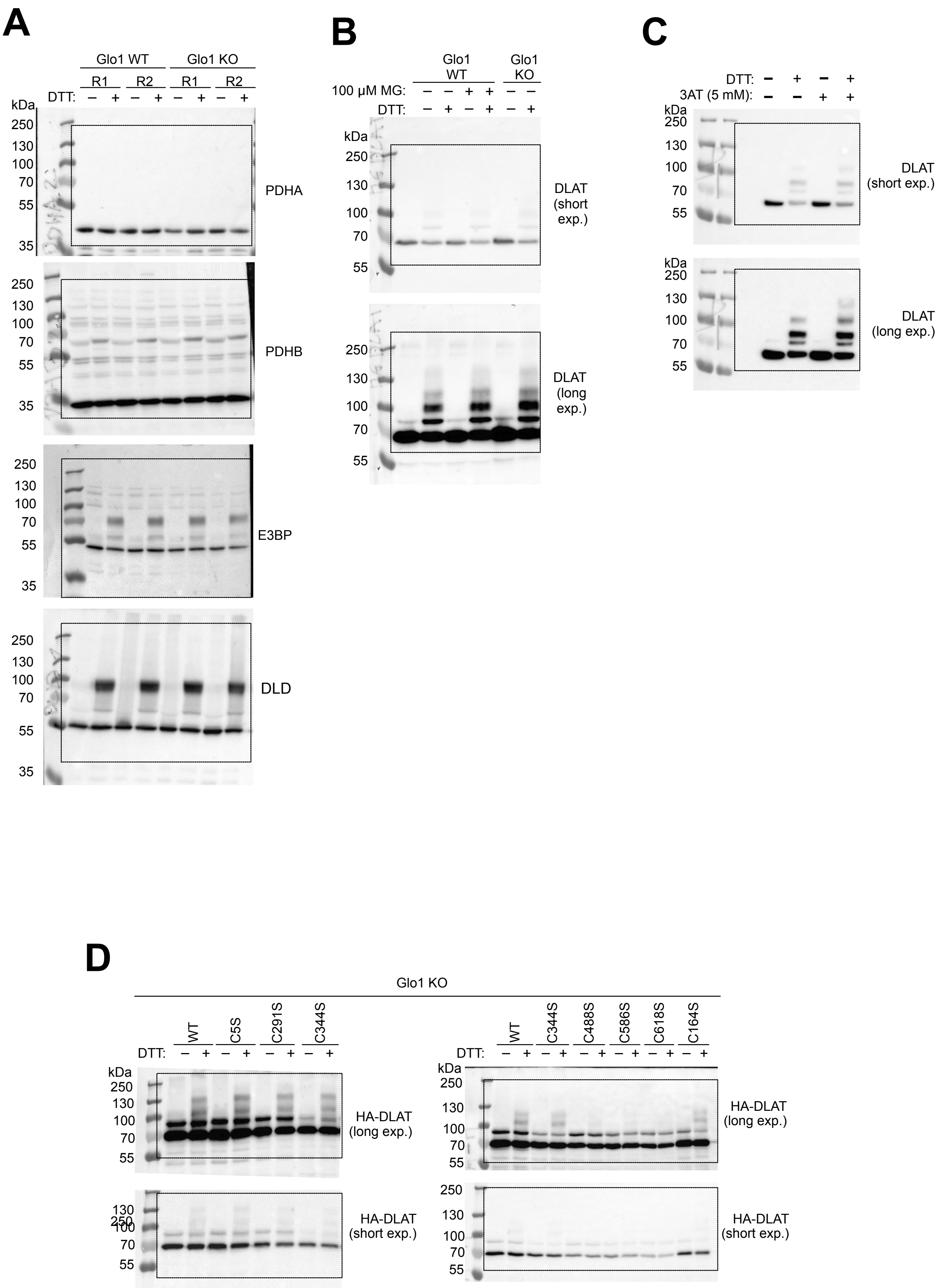

Supplementary Figure 7

B

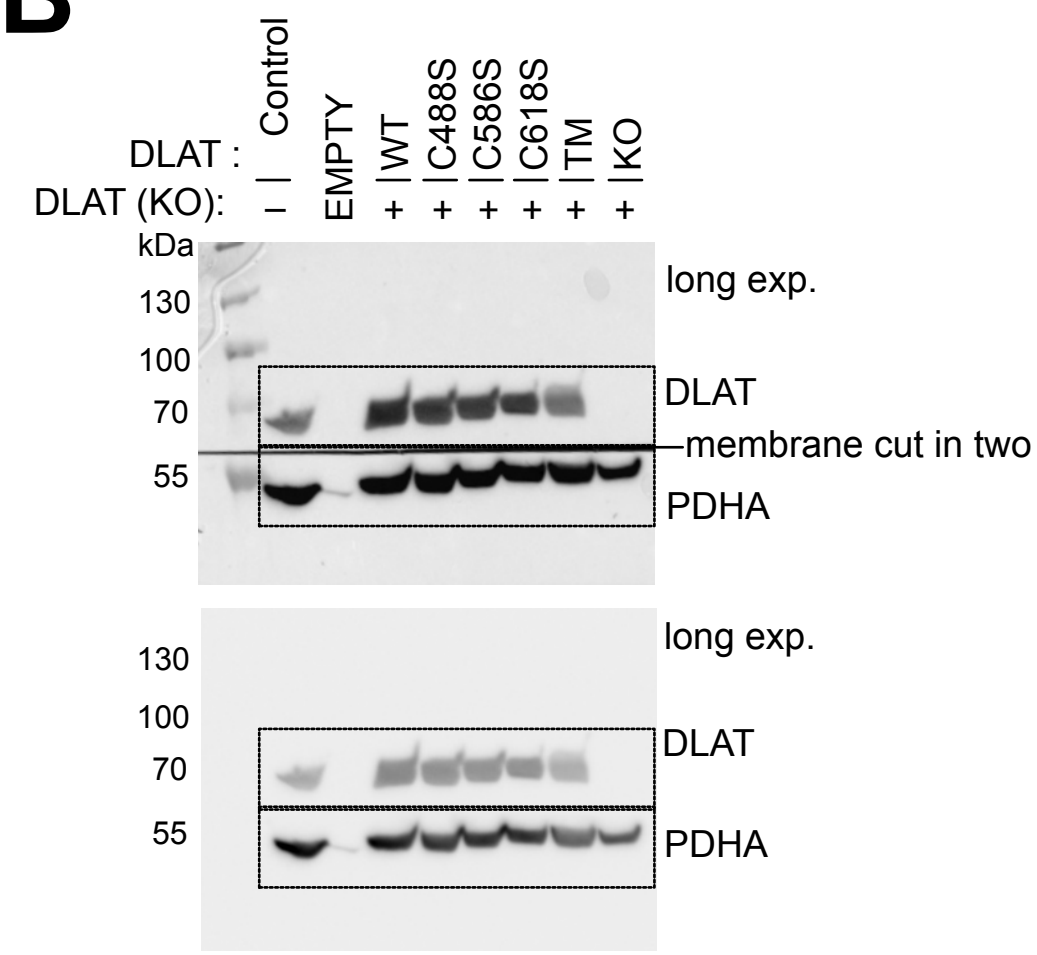

Supplementary Figure 8

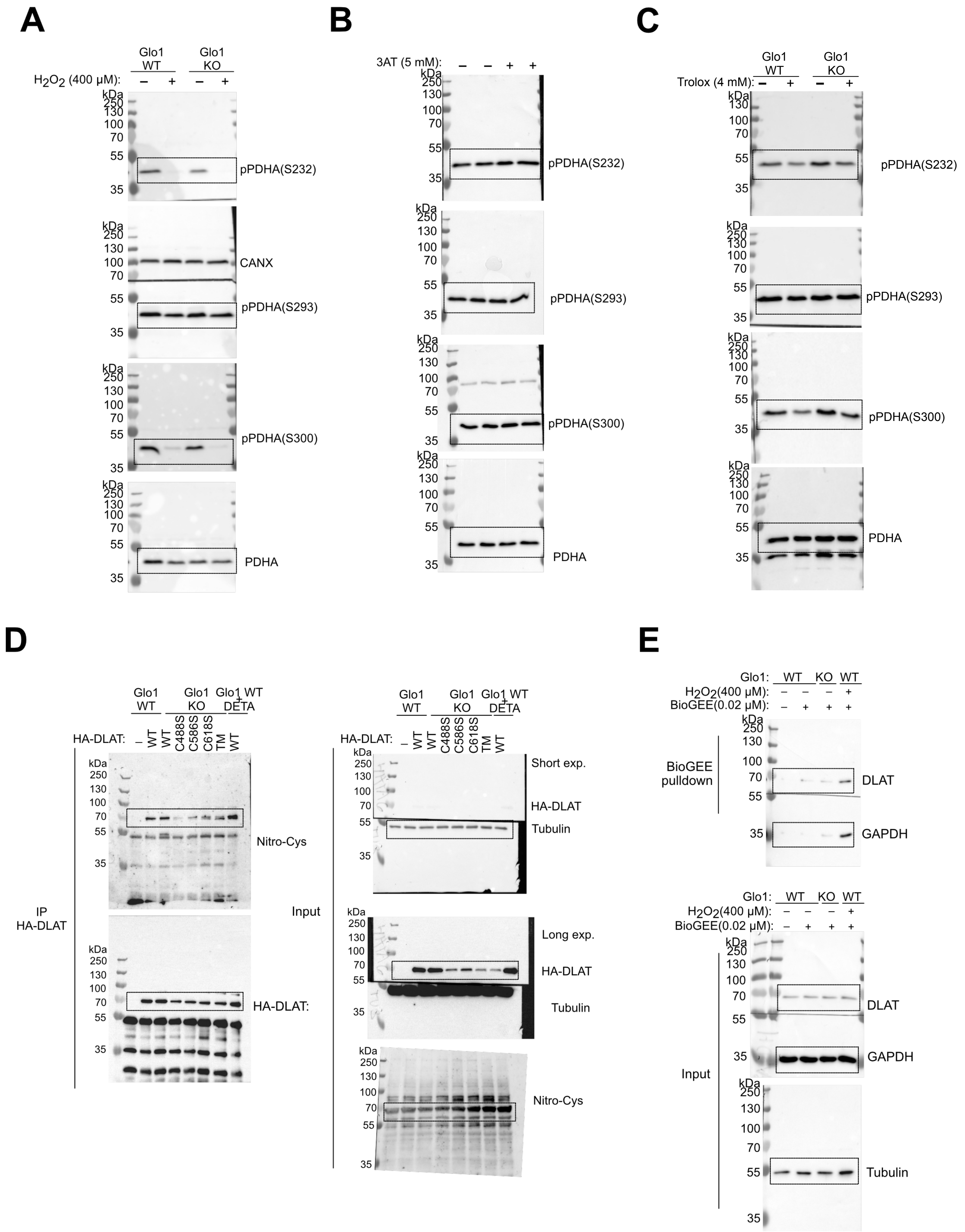

Supplementary Figure 10

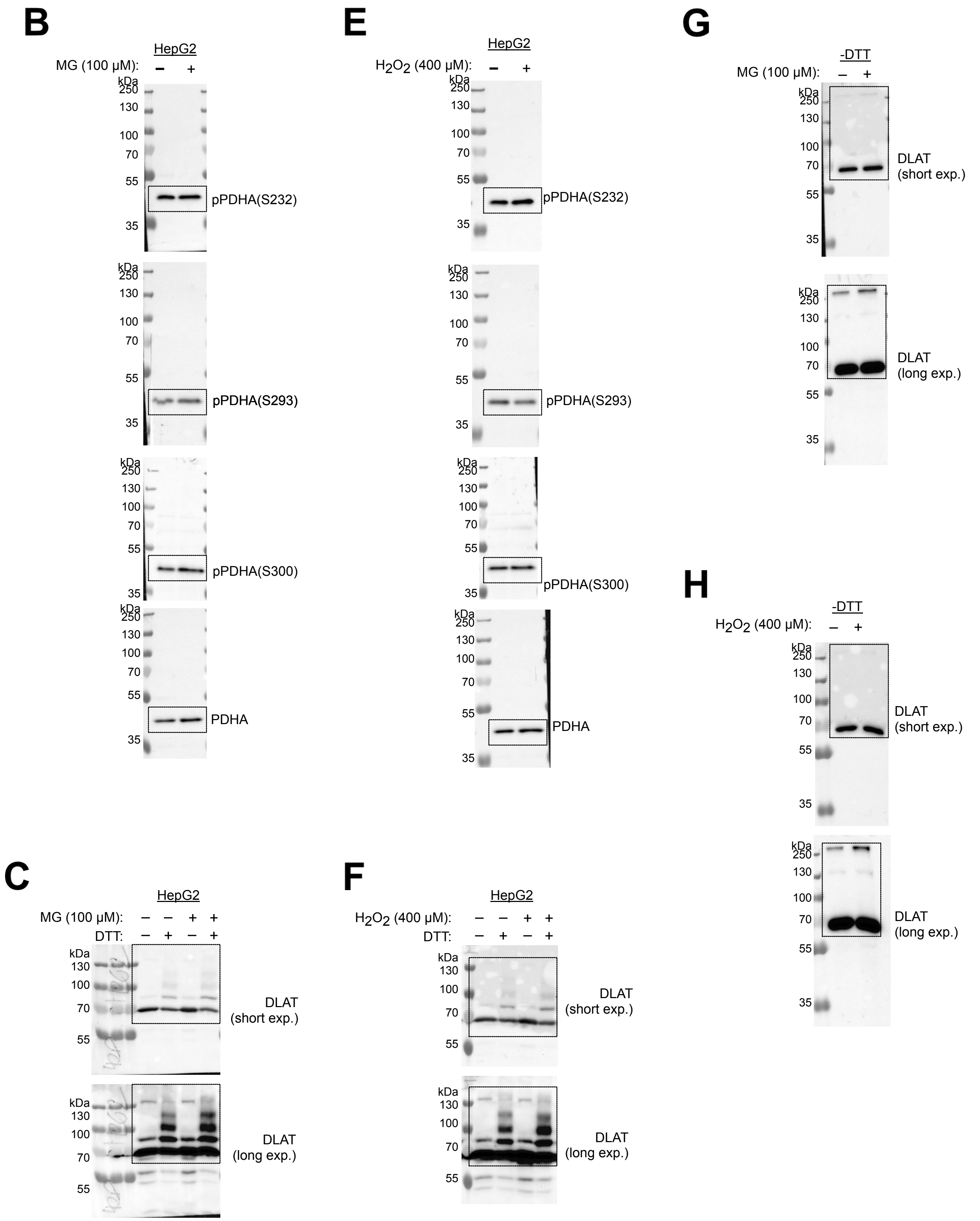

Supplement: Supplementary file 1 [file 41467_2026_78266_MOESM1_ESM.pdf]
