# Supplementary material for: Regulation of PDH via cysteine oxidation on DLAT
Source: Nat Commun. 2026 Oct 14;17:10784. doi: 10.1038/s41467-026-78266-6 (PMC13669365; doi:10.1038/s41467-026-78266-6)
Supplement: Supplementary file 2 [file 41467_2026_78266_MOESM2_ESM.pdf]

Description of Additional Supplementary Files

Supplementary Data 1: Metabolomics data.
Supplementary Data 2: clinical parameters
Supplementary Data 3: Sequences of oligos and siRNAs.
