# Supplementary material for: Regulation of PDH via cysteine oxidation on DLAT
Source: Nat Commun. 2026 Oct 14;17:10784. doi: 10.1038/s41467-026-78266-6 (PMC13669365; doi:10.1038/s41467-026-78266-6)
Supplement: Supplementary file 3 [file 41467_2026_78266_MOESM6_ESM.pdf]

nature portfolio

Corresponding author(s): Aurelio Teleman

Last updated by author(s): 25/8/2026

Reporting Summary

Nature Portfolio wishes to improve the reproducibility of the work that we publish. This form provides structure for consistency and transparency in reporting. For further information on Nature Portfolio policies, see our Editorial Policies and the Editorial Policy Checklist.

Statistics

For all statistical analyses, confirm that the following items are present in the figure legend, table legend, main text, or Methods section.

n/a	Confirmed	
☐	☒	The exact sample size (*n*) for each experimental group/condition, given as a discrete number and unit of measurement
☐	☒	A statement on whether measurements were taken from distinct samples or whether the same sample was measured repeatedly
☐	☒	The statistical test(s) used AND whether they are one- or two-sided<br>*Only common tests should be described solely by name; describe more complex techniques in the Methods section.*
☒	☐	A description of all covariates tested
☐	☒	A description of any assumptions or corrections, such as tests of normality and adjustment for multiple comparisons
☐	☒	A full description of the statistical parameters including central tendency (e.g. means) or other basic estimates (e.g. regression coefficient) AND variation (e.g. standard deviation) or associated estimates of uncertainty (e.g. confidence intervals)
☐	☒	For null hypothesis testing, the test statistic (e.g. *F*, *t*, *r*) with confidence intervals, effect sizes, degrees of freedom and *P* value noted<br>*Give P values as exact values whenever suitable.*
☒	☐	For Bayesian analysis, information on the choice of priors and Markov chain Monte Carlo settings
☒	☐	For hierarchical and complex designs, identification of the appropriate level for tests and full reporting of outcomes
☒	☐	Estimates of effect sizes (e.g. Cohen's *d*, Pearson's *r*), indicating how they were calculated

Our web collection on statistics for biologists contains articles on many of the points above.

Software and code

Policy information about availability of computer code

Data collection

The software running the Biorad ChemiDoc is commercially available. For analysis, ImageLab 5.2.1 software was used, which is freely available on the BioRad website.

Data analysis

All software used in this study is publicly or commercially available:

Data analysis:
Image Lab 5.2.1
Microsoft Excel 16.16.27
GraphPad Prism 9.0.2

Figure preparation: Affinity Photo v1 and Affinity Designer v1

For manuscripts utilizing custom algorithms or software that are central to the research but not yet described in published literature, software must be made available to editors and reviewers. We strongly encourage code deposition in a community repository (e.g. GitHub). See the Nature Portfolio guidelines for submitting code & software for further information.

Data

Policy information about availability of data

All manuscripts must include a data availability statement. This statement should provide the following information, where applicable:

- Accession codes, unique identifiers, or web links for publicly available datasets
- A description of any restrictions on data availability
- For clinical datasets or third party data, please ensure that the statement adheres to our policy

All data supporting the findings of this study are available within the paper and its Supplementary Information.

Research involving human participants, their data, or biological material

Policy information about studies with human participants or human data. See also policy information about sex, gender (identity/presentation), and sexual orientation and race, ethnicity and racism.

Reporting on sex and gender	The sex of the participants is reported in the Suppl. Table 1.
Reporting on race, ethnicity, or other socially relevant groupings	n/a
Population characteristics	n/a
Recruitment	Reported in M&M. For liver biopsies, participants were prospectively recruited at the Study Center for Diabetes and Metabolism, University Hospital Heidelberg, among individuals scheduled for bariatric surgery who met the indication for surgery according to national guidelines. All participants provided written informed consent prior to study procedures. For muscle biopsies, healthy volunteers were prospectively recruited for the FFAIR study at the Study Center for Diabetes and Metabolism, University Hospital Heidelberg, and provided written informed consent prior to study procedures.
Ethics oversight	Reported in M&M.

Note that full information on the approval of the study protocol must also be provided in the manuscript.

Field-specific reporting

Please select the one below that is the best fit for your research. If you are not sure, read the appropriate sections before making your selection.

☒ Life sciences ☐ Behavioural & social sciences ☐ Ecological, evolutionary & environmental sciences

For a reference copy of the document with all sections, see nature.com/documents/nr-reporting-summary-flat.pdf

Life sciences study design

All studies must disclose on these points even when the disclosure is negative.

Sample size	No sample size calculations were performed. Statistical analyses were done on the data to check for statistical significance, as indicated in the figures.
Data exclusions	No data were excluded.
Replication	All experiments were repeated two to three times with independent biological replicates. All attempts at replication were successful. For immunofluorescence and biochemical experiments, one representative experiment is shown.
Randomization	No randomization was used to allocate samples in experiments comparing different cell lines. When comparing different treatments within the same cell line, samples were assigned randomly to control or treatment group.
Blinding	No blinding was used since experiments required frequent intervention by investigators to maintain cell lines, thus precluding effective blinding.

Reporting for specific materials, systems and methods

We require information from authors about some types of materials, experimental systems and methods used in many studies. Here, indicate whether each material, system or method listed is relevant to your study. If you are not sure if a list item applies to your research, read the appropriate section before selecting a response.

Materials & experimental systems

n/a	Involved in the study
	☒ Antibodies
	☒ Eukaryotic cell lines
☒	Palaeontology and archaeology
	☒ Animals and other organisms
	☒ Clinical data
☒	Dual use research of concern
☒	Plants

Methods

n/a	Involved in the study
☒	ChIP-seq
☒	Flow cytometry
☒	MRI-based neuroimaging

Antibodies

Antibodies used

The following primary antibodies were used:
CEL (Cell Signaling Technology #44358)
DLAT (Cell Signaling Technology #12362)
DLAT (Proteintech, #13426-1-AP)
DLD (Santa Cruz Biotechnology sc-365977)
E3BP (Santa Cruz Biotechnology sc-393644)
FLAG tag (Cell Signaling Technology #14693)
GAPDH (Cell Signaling Technology #2118)
Glutathione (D8) (Santa Cruz Biotechnology sc-52399)
HA tag (Cell Signaling Technology #2367)
LDH (Cell Signaling Technology #2012)
MTC1 (Cell signaling Technology #85680)
PDHA (Cell Signaling Technology #3205)
MG-H1 ref. 48
PDHB (Proteintech #14744-1-AP)
pPDH(S232) (Proteintech #29582-1-150)
pPDH(S297) (Abcam ab92696) was used 1:5000
pPDH(S232) (Proteintech #80572-1-RR)
Pyruvate dehydrogenase E2/E3bp (Abcam ab110333)
S-Nitroso-Cys (Biozol Diagnostica APD NISC11-A)
Tubulin (Sigma Aldrich #T9026) was used 1:5000
VDAC (Cell Signaling Technology #4661)

All antibodies were diluted 1:1000 unless indicated otherwise. All antibodies were validated on the manufacturer's website, except DLAT which was validated with a KO in this study, and MG-H1 which was validated in ref. 48.

Secondary antibodies were used 1:10,000 and are the following:
anti-rabbit HRP (Jackson ImmunoResearch #111-035-003)
anti-mouse HRP (Jackson ImmunoResearch #115-035-003)
anti-guinea pig HRP (Jackson ImmunoResearch #106-035-003)

Validation

Primary antibodies were validated on the manufacturer's websites, except for MG-H1 antibody, which was validated in ref. 48

Eukaryotic cell lines

Policy information about cell lines and Sex and Gender in Research

Cell line source(s)

HEK293T: ATCC #CRL-3216

Authentication

None

Mycoplasma contamination

No mycoplasma contamination was detected by qPCR.

Commonly misidentified lines
(See ICLAC register)

None of these cell lines were used.

Animals and other research organisms

Policy information about studies involving animals; ARRIVE guidelines recommended for reporting animal research, and Sex and Gender in Research

Laboratory animals	Species: mouse<br>Strains: C57BL/6N and Glo1-/-<br>Age: For DLAT oxidation in different tissues of control mice: 10-week-old. For HGHCi mice, 16-week-old.
Wild animals	None
Reporting on sex	For DLAT oxidation in different tissues of control mice, male mice were used. For the HGHCi mice, both male and female mice were used.
Field-collected samples	None
Ethics oversight	All animal experiments were approved by the ethical authorities (Regierungspräsidium Karlsruhe, Germany) and performed in compliance with the German Animal Welfare Act for the care and use of laboratory animals, with the rules of the regulatory authorities in Baden-Württemberg, with the ARRIVE guidelines and with the guidelines of Directive 2010/63/EU.<br><br>Experiments with the GLO1KO mice were approved by the ethical authorities (Regierungspräsidium Karlsruhe, Germany), approval numbers 35-9185.81/G-144/21.<br><br>For wildtype animals, all procedures were approved by the Animal Care and Use Committee at the regional authority in Karlsruhe, Germany (T-94/14).

Note that full information on the approval of the study protocol must also be provided in the manuscript.

Clinical data

Policy information about clinical studies

All manuscripts should comply with the ICMJE guidelines for publication of clinical research and a completed CONSORT checklist must be included with all submissions.

Clinical trial registration	Liver biopsies: ChARMeD study, local ethic number S-887/2021, registered at the German Clinical Trials Register DRKS00028484<br>Muscle biopsies: FFAIR study, local ethic number S-883/2021, registered at the German Clinical Trials Register DRKS00028505
Study protocol	For both studies, summary of study protocol is provided in the corresponding registration site at the German Clinical Trials Register. A full study protocol (original version in German) can be provided from the principal investigator of the study upon request. The study was approved by the ethics committee of Heidelberg University
Data collection	Both studies are currently ongoing, and participant recruitment is being conducted at the Clinic of Endocrinology, Diabetology, Metabolic Diseases, and Clinical Chemistry (Internal Medicine 1) at the University Hospital of Heidelberg, Germany. Participant evaluation and data collection are carried out at the Study Center for Diabetes and Metabolism at the University Hospital of Heidelberg.<br>Recruitment in the ChARMeD study started on July 7, 2022.<br>Recruitment in the FFAIR study started on August 28, 2023.
Outcomes	ChARMeD study:<br>Primary outcome: change in insulin sensitivity, energy metabolism and lipid metabolism in liver, skeletal muscle and adipose tissue after bariatric surgery.<br>Secondary outcome: Detailed characterization of metabolically ill obese individuals, particularly with respect to mitochondrial function in liver, skeletal muscle, and adipose tissue.<br><br>The study program of the ChARMeD study is organized in mandatory and optional modules:<br>Module 1: Basic Module (Mandatory) at baseline (within 6 weeks prior to surgery), 6, 12 & 24 months postoperatively<br>- Medical history collection (pre-existing conditions, previous surgeries, if applicable for type 2 diabetes patients: blood sugar control, known diabetes complications)<br>- Somatometry (weight, height, waist circumference, hip circumference, neck circumference, waist-to-hip ratio), vital signs (blood pressure, heart rate)<br>- Handgrip strength measurement<br>- Bioelectrical impedance analysis (including phase angle)<br>- Resting ECG<br>- Basic ultrasound examination (intima media thickness, liver diameter, kidney ultrasound and Renal Resistive Index)<br>- Liver stiffness (Fibroscan)<br>- Fasting blood sample collection (among others: cell blood count, liver transaminases, creatinine, urea, eGFR, hsCRP, troponin T, NTproBNP, leptin, adiponectin), albumin-to-creatinine-ratio<br>- Mixed meal test (+ plasma samples for gut peptide determination, +VAS hunger/satiety at timepoints 0', 30', 60', 90', 120', 150', 180' minutes)<br>- Advanced Glycation End-products (AGEs) skin fluorescence measurement<br>- Clinical neuropathy screening score (neuropathy symptoms score (NSS), neuropathy disability score (NDS))<br>- Questionnaires (SF12, Reflux Symptom Index – RSI, Pittsburgh Sleep Quality Index – PSQI, Sigstad's Dumping Scoring Questionnaire, Dumping Symptom Rating Scale)

- Blood samples for biobank (centrifuged plasma & serum, whole blood, white blood cells)
- Stool sample collection (for gut microbiome analysis)
- Bone density measurement through DXA scan (only if clinically indicated)
- Intraoperative collection of subcutaneous/visceral adipose (SAT/VAT) tissue samples
- Intraoperative liver biopsy
- Intraoperative skeletal muscle biopsy (Rectus abdominis)

Module 2: Metabolism Module (Optional) at baseline (within 6 weeks prior to surgery), 6, 12 & 24 months postoperatively
- Euglycemic Hyperinsulinemic Clamp (EEC)
- Indirect calorimetry (before and after EEC)
- 24-hour urine collection (nitrogen determination)
- Muscle biopsy (M. V. lateralis)
- SAT biopsy with 6-mm Bergström needle

Module 3: Neuropathy Module (Optional) at baseline (within 6 weeks prior to surgery), 4 weeks, 6, 12 & 24 months postoperatively
- Nerve conduction velocity (N. suralis, N. peroneus, N. tibialis, N. medianus, N. ulnaris)
- Quantitative sensory testing (CDT, WDT, TSL, CPT, HPT, MDT, MPS, WUR, DMA, VDT, PTT)
- Heart rate variability (HRV at rest, E/I ratio, 30:15)
- Peg-board test
- Skin biopsy
- Corneal confocal microscopy
- (only at the 4-week postoperative visit) fasting blood sample collection, including blood samples for biobank (see Basic Module)

Module 4: Cardiovascular Module (Optional) at baseline (within 6 weeks prior to surgery), 6, 12 & 24 months postoperatively
- Echocardiography
- Pulmonary function test
- 6-minute walk test
- Ankle-brachial-index, pulse wave velocity
- Cardiac MRI

Module 5: Imaging Module (Optional) at baseline (within 6 weeks prior to surgery), 6, 12 & 24 months postoperatively
- 1H-MRS of the liver
- 31P-MRS of the liver
- Whole-body fat distribution and muscle mass
- Liver vessel imaging
- BOLD-MRI of the kidney

FFAIR study:
Primary outcome: mitochondrial function measured by mitochondrial respiration in skeletal muscle and SAT, accumulation of reactive metabolites and AGEs in skeletal muscle and SAT, detoxification of reactive metabolites (measurement of glyoxalase and aldo-keto reductase activity)
Secondary outcome: investigation of the combined effects of hyperlipidemia, hyperglycemia, and hyperinsulinemia on tissue-specific metabolism in skeletal muscle and SAT, as well as on circulating markers of mitochondrial stress.

The study program of the FFAIR study includes:
- Anamnesis (questionnaire) and clinical basic examination: medical history, blood sugar control, known diabetes complications, medication, allergies, height, weight, waist, and hip circumference. A three-day dietary protocol (according to the German Nutrition Society [DGE]) is maintained before the clamp examination. Validated questionnaires (SF-12, PHQ-D) assess quality of life and health status.
- basic laboratory in blood samples: complete blood count (white blood cells, red blood cells, platelets, hemoglobin, hematocrit, mean corpuscular hemoglobin, mean corpuscular volume, mean corpuscular hemoglobin concentration, sodium, potassium, calcium, magnesium, phosphate, triglycerides, total cholesterol, LDL cholesterol, HDL cholesterol, Lp(a), creatinine, suPAR, calculated GFR, GOT/AST, GPT/ALT, GGT, alkaline phosphatase, cholinesterase, hsCRP, total bilirubin, urea, uric acid, ferritin, transferrin, transferrin saturation, iron, total protein, albumin
- urine analysis (spot urine): urine status (test strips), albumin, protein.
- Hormones, diabetes, glucose: fasting glucose, C-peptide, HOMA-IR, HbA1c, TSH.
- Markers of dicarbonyl Stress: dicarbonyl compounds, including methylglyoxal, glyoxal, N(ε)-(carboxymethyl)lysine (CML), N(ε)-(carboxyethyl)lysine (CEL), and oxidative stress-related AGEs (advanced glycation end products) in plasma and urine.
- DNA damage and repair mechanisms: RAGE and γH2AX expression in peripheral mononuclear blood cells.
- Activation of protective mechanisms: Glyoxylase 1 and 2, aldoketo-reductase, aldehyde dehydrogenase activity and expression, glutathione oxidized/reduced ratio (GSH/GSSG), MnSOD, catalase.
- Protein quality control system: Hsp70, Vms1.
- Senescence markers: suPAR, INK4A, INK4B, Cip1, β-galactosidase, TGF-beta, IL-6.
- Samples for biobank: plasma for metabolomics/biomarkers: various stabilized plasma samples (Li-heparin, EDTA plasma, peripheral blood mononuclear cell isolation, whole blood, citrate blood, serum, spot urine, 24-hour urine collection) collected in a fasting state for baseline characterization and during intervention (clamp examination) to identify biomarkers associated with diabetes complications.
- Anthropometry: weight, height, BMI, waist circumference
- Resting ECG
- Blood pressure measurement
- Oral glucose tolerance test (0, 30, 60, 90, and 120 minutes)
- Hyperinsulinemic-Euglycemic Clamp with two treatments: lipid Infusion: using a lipid emulsion (ClinOleic®) and control Infusion: with glycerol. Participants are randomly assigned to a sequence of treatments, performed on separate days.
- Indirect calorimetry
- Tissue biopsies (muscle from the quadriceps (vastus lateralis) and subcutaneous abdominal fat) sampled through aspiration biopsy before and after lipid/glycerol infusion as well as after insulin.

Plants

Seed stocks	*Report on the source of all seed stocks or other plant material used. If applicable, state the seed stock centre and catalogue number. If plant specimens were collected from the field, describe the collection location, date and sampling procedures.*
Novel plant genotypes	*Describe the methods by which all novel plant genotypes were produced. This includes those generated by transgenic approaches, gene editing, chemical/radiation-based mutagenesis and hybridization. For transgenic lines, describe the transformation method, the number of independent lines analyzed and the generation upon which experiments were performed. For gene-edited lines, describe the editor used, the endogenous sequence targeted for editing, the targeting guide RNA sequence (if applicable) and how the editor was applied.*
Authentication	*Describe any authentication procedures for each seed stock used or novel genotype generated. Describe any experiments used to assess the effect of a mutation and, where applicable, how potential secondary effects (e.g. second site T-DNA insertions, mosiacism, off-target gene editing) were examined.*
