# Supplementary material for: Regulation of PDH via cysteine oxidation on DLAT
Source: Nat Commun. 2026 Oct 14;17:10784. doi: 10.1038/s41467-026-78266-6 (PMC13669365; doi:10.1038/s41467-026-78266-6)
Supplement: Supplementary file 4 [file 41467_2026_78266_MOESM7_ESM.pdf]

nature portfolio

Peer Review File

Regulation of PDH via cysteine oxidation on DLAT

Corresponding Author: Dr Aurelio Teleman

This file contains all reviewer reports in order by version, followed by all author rebuttals in order by version.

Version 0:

Reviewer comments:

Reviewer #1

(Remarks to the Author)
Garcia-Cortizo et al describes an interesting finding as to regulation of PDH through cysteine oxidation on DLAT. The authors found that PDH activity is inhibited by knockout of Glyoxalase1(GLO1), which detoxifies methylglyoxal (MG), as well as MG treatment. They further identified Cys488, Cys586 or Cys618 of DLAT are critical for the MG-dependent change in PDH activity because all C to S DLAT mutation abolished oxidation on DLAT and H2O2-dependent decrease in PDH activity. They also demonstrated that PDH oxidation can be regulated in mice with MG and signifiant correlation between PDH oxidation and activity was observed in human liver samples. Although these findings are quite interesting and might provide an important insights into PDH complex regulation by oxidation status, the current manuscript does not have any functional studies as to how cysteine oxidation of DLAT affects overall metabolism and cell properties as well as any diseases. These shortcomings make this study fall short of impact and significance. Specific comments are below.

Major comments
1. Does all C to S DLAT mutation affect any cellular functions such as cell proliferation, migration, and/or adhesion in response to MG? MG is one of major metabolites associated with diabetes, liver dysfunction and atherosclerosis. It is important to examine if all C to S DLAT mutation affects any of these disease progressions (e.g. HFD-induced diabetes).

2. In line with the comment above, MG is known to regulate cancer growth as well but with more complexity because low dose of MG promotes while high dose of MG inhibits tumor growth. Does all C to S DLAT mutation affect any tumor metabolism and growth in response to MG? The authors should test if all C to S DLAT mutant expression would affect TCA cycle, cancer cell proliferation, and tumor growth in mice in response to MG treatment if they would focus on cancer.

Minor comments
3. It is unclear if C to S mutation on DLAT would affect basal activity of PDH. Such information would be helpful for readers to understand regulation of PDH by cysteine oxidation.

Reviewer #2

(Remarks to the Author)
This paper makes the provocative claim that PDH regulation by cysteine oxidation on the DLAT subunit (E2) supersedes the well-known regulation via E1-alpha phosphorylation. If true, this would be a very important finding with great relevance. The PDH complex sits at the critical junction between the Warburg effect and mitochondrial oxidative phosphorylation, and its regulation (whether activated or inactivated) determines the cellular source of acetyl-CoA. Overall, the paper is nicely written. However, I do not feel the conclusions are sufficiently supported by the experiments and data presented. Most experiments lack important control and rigorous methodology. Significantly more rigorous and compelling data are needed to convincingly conclude that DLAT oxidation is a bona fide regulatory mechanism for PDH activity and flux.

1. Concerns About PDH Activity Measurement
I have major concerns regarding how PDH activity is measured. The assay used essentially monitors NAD-to-NADH conversion via an enzymatic kit. It requires the isolation of mitochondria and subsequent incubation with substrates for the PDH complex (including pyruvate and NADH). First, there is no confirmation of mitochondrial purity. It's extremely difficult to purify mitochondria free to cytosolic or other organelle contaminants. Even IP of mitochondria can pull down cytosolic lactate dehydrogenase (LDH) and other membrane organelles. Many cytosolic enzymes that also use NAD/NADH (e.g., LDH,

which uses NAD and pyruvate) could co-purify with mitochondria. Second, the assay runs for at least 15–20 minutes, during which substantial changes in the mitochondrial content might occur. In addition, there is no clear normalization for the assay. For example, how are the PDH activities in GLO1^WT and GLO1 KO cells normalized when they come from different cell lines and distinct mitochondrial preparations?

In addition, the tracing data in Figure 1K lack important controls. Citrate M+2 tracing from U13C Glucose should be conducted over a time course (e.g., 2, 5, 10, 15 minutes) and normalized to pyruvate M+3 tracing if U-13C glucose is used as the tracer. The α-KG, succinate, and fumarate tracing results show very low percentage values (single-digit or even 0.1%), which raises questions about their accuracy. While 13C tracing would indeed be a more reliable indicator of PDH activity, more rigorous execution and replication of these experiments are needed.

2. Correlational Nature of the Data
Much of the data is correlative. The characterization of GLO1-deficient cells and their MG levels is nicely done. However, the conclusion that GLO1 deficiency leads to increased mitochondrial redox stress, which then results in decreased PDH activity, is highly speculative. These conclusions rely primarily on correlations between PDH activity (already in question) and treatments that raise or lower mitochondrial ROS. Similarly, there is no direct evidence that cysteine oxidation of DLAT at the three serine residues actually regulates PDH activity or flux. Demonstrating this would require a detailed characterization of DLAT oxidation biochemically or genetically (see below) and its manipulation, followed by rigorous PDH activity assay. In addition, PDH activity is regulated acutely (within seconds to minutes). Most of the authors treatment (i.e. GLO1 KO or drug treatment) spans hours to days, during which time the cells would have had time to undergo other secondary changes.

3. Weak Characterization of DLAT Oxidation
The characterization of DLAT oxidation is weak both biochemically and genetically. Biochemically, the authors should employ more established methods for studying DLAT disulfide bond formation, such as mass spectrometry or chromatography, or perform assays with purified DLAT protein. Genetically, the authors should generate DLAT knockout cells (rather than relying on cells expressing endogenous WT DLAT) and perform mutagenesis studies to assess the functional consequences of specific cysteine oxidations.

Reviewer #3

(Remarks to the Author)
The paper explores PDH regulation and this is an interesting area as 4 of the 5 kinases within mitochondria regulate it. PDH integrates a wide range of metabolic signals and is a key node in cancer. While the work is explores and interesting area I have a number of significant concerns.

1 The authors use the term ROS as if its one chemical with one reactivity and one type of target. They also move between MG and H_2O_2 as if they are equivalent when the former irreversibly alkylates thiols and amines and the latter reversibly oxidises cysteines. The authors should rewrite the paper so that it makes less use of fuzzy concepts like ROS and more use of the specific chemistry the authors believe is occurring.

2 Currently the MG and H_2O_2 parts of the paper are not well linked. Its not clear to this reviewer why MG which is a glycolysis breakdown product from upstream glucose should have the same regulatory outcome as H_2O_2 which probably forms from downstream O_2. The manuscript suggests MG acts via upregulation of H_2O_2 by an unidentified mechanism. The rationale for moving to H_2O_2 seems to be increased DCFDA signal from MG, but DCFDA is not a clean tool and it isn't compartmentalised. The authors should prove this link by showing that MG causes increased mitochondrial H_2O_2/O_2.- or shifts the redox state in the vicinity of PDH by measuring peroxiredoxin 3 dimerisation and/or aconitase inactivation.

3 The 400 uM H_2O_2 concentrations used here and long incubation times might harm/kill HEK cells. Many processes could be negatively and non-specifically affected under these harsh and unlikely to be physiological conditions. More needs to be done to show that the loss of PDH activity under these settings is a specific reversible regulatory response. This is particularly true since lower H_2O_2 concentrations (100-200 µM for 5 min) in the same cell lines have the opposite affect via oxidation of thiols on PDHK2 (doi: 10.1074/jbc.M112.400002, doi: 10.1074/jbc.M703591200).

4 The authors conclude that PDH oxidation dominates phosphorylation. The authors should titrate H_2O_2 into their system and measure PDH phosphorylation and activity to show the H_2O_2 concentration range this is true for. Furthermore they should show that loss in PDH activity is reversible after an hour of H_2O_2 exposure if the oxidant is removed.

5 Finally, I have concerns about exploring PDH regulation solely in highly glycolytic transformed cell lines as the results may have limited applicability. The paper would benefit from using fresh isolated heart mitochondria and showing that MG and much lower H_2O_2 concentrations (~10 uM as there is no cytosolic GSH/GPerox system to pass through) can cause similar PDH regulation.

Version 1:

Reviewer comments:

Reviewer #1

(Remarks to the Author)
The authors adequately addressed this reviewer's concerns using triple C to S mutant of DLAT. Just one minor concerns is that the Y -axis of tumor growth curve in Fig. 4H might be cm3 not mm3. Also it is recommended to show tumor weights from the xenograft study in Fig. 4H.

Reviewer #2

(Remarks to the Author)
I appreciate the authors' extensive efforts in addressing my concerns and the new data. As indicated in the title: "Regulation of PDH via cysteine oxidation on DLAT" and the abstract, the KEY message and novelty is the discovery of a new PDH activity regulation via cysteine oxidation. This really ought to be the emphasis of the paper and need to be more strongly supported. Without definitively showing this to be true, the remaining physiological/in vivo experiments do not have sufficient scientific rationale. I understand that other reviewers requested more physiological/in vivo relevance that are in figures 4-6, but the most significant finding in my opinion is that PDH complex can be regulated by DLAT (E2) oxidation despite many decades of studies on E1alpha phosphorylation.

To this end, with the exception of 1 cell-based 13C tracing figure (supplemental figure 3, which uses only Glo KO instead of direct DLAT oxidation manipulation), the authors' PDH activity assays have relied extensively on an in vitro PDH activity assay that is not a standard. I understand there are technical limitations in other aspects, however, U13C glucose tracing as the authors have done in the paper, is easily doable and a much more robust, unbiased, and physiological readout of PDH activity in intact cells.

For me to be convinced of the central conclusion, I would really need to see the following two U13C tracing experiments, which should not take more than 1-2 weeks and for which the authors have already have all the cell lines/reagents/demonstrated capability:
1. U13C glucose tracing in H2O2 treated cells - the authors results should predict a decrease in Cit m+2/Pyr m+3 with H2O2 due to suppressed PDH
2. U13C glucose tracing in DLAT KO cells reconstituted with WT or C-S mutant DLAT - the authors results would predict an increase in Cit m+2/Pyr m+3 in the C-S mutant.

I also encourage the author to move supplemental figure 3B into the main paper as U13C tracing is a much more widely accepted and physiologic readout of PDH activity in cells. I would encourage authors to remove Succinate and aKG as the readout of PDH activity is a ratio of citrate m+2/pyruvate m+3.

Reviewer #3

(Remarks to the Author)

Open Access This Peer Review File is licensed under a Creative Commons Attribution 4.0 International License, which permits use, sharing, adaptation, distribution and reproduction in any medium or format, as long as you give appropriate

credit to the original author(s) and the source, provide a link to the Creative Commons license, and indicate if changes were made.
In cases where reviewers are anonymous, credit should be given to 'Anonymous Referee' and the source.
The images or other third party material in this Peer Review File are included in the article's Creative Commons license, unless indicated otherwise in a credit line to the material. If material is not included in the article's Creative Commons license and your intended use is not permitted by statutory regulation or exceeds the permitted use, you will need to obtain permission directly from the copyright holder.
To view a copy of this license, visit https://creativecommons.org/licenses/by/4.0/

Reviewer #1

Garcia-Cortizo et al describes an **interesting finding** as to regulation of PDH through cysteine oxidation on DLAT. The authors found that PDH activity is inhibited by knockout of Glyoxalase1(GLO1), which detoxifies methylglyoxal (MG), as well as MG treatment. They further identified Cys488, Cys586 or Cys618 of DLAT are critical for the MG-dependent change in PDH activity because all C to S DLAT mutation abolished oxidation on DLAT and H2O2-dependent decrease in PDH activity. They also demonstrated that PDH oxidation can be regulated in mice with MG and signifiant correlation between PDH oxidation and activity was observed in human liver samples. **Although these findings are quite interesting and might provide an important insights into PDH complex regulation** by oxidation status, the current manuscript does not have any functional studies as to how cysteine oxidation of DLAT affects overall metabolism and cell properties as well as any diseases. These shortcomings make this study fall short of impact and significance. Specific comments are below.

We thank the reviewer for the encouraging words and for the constructive suggestions.

We agree with the reviewer that our original submission was missing functional studies on the downstream consequences of regulation of PDH by oxidation. We have now done an extensive set of additional experiments to address this point, leading to multiple new figures, which we believe significantly strengthen the manuscript. We now provide the following new data:

1. We generated DLAT knockout cells (which surprisingly are viable). These cells have no DLAT protein (Suppl. Fig. 7B) and no PDH activity (Suppl. Fig. 7C). As expected, these cells are highly glycolytic, and acidify the culture medium extremely quickly. We then stably reconstituted these knockouts with either wildtype DLAT or mutant DLAT where Cys488, Cys586 and Cys618 are mutated to serine and therefore cannot be oxidized. As requested by the reviewer, this allowed us to perform functional studies on the effects of DLAT oxidation on metabolism and other cellular properties.
2. We then tested the effect of these mutations on PDH activity. As expected, mutation of these three cysteines to serine does not impair PDH activity. In fact, if anything, it increases baseline PDH activity (Suppl. Fig. 7C-D). This is expected because oxidation on these cysteines reduces PDH activity (Fig. 2) and there is some baseline oxidation on these cysteines in the absence of oxidative stress in cell lines in culture (Fig. 2G), in vivo in humans (Fig. 6A,E) and in vivo in mice (Fig. 5E-G).
3. Consistent with the data in our original submission, mutation of these three cysteines causes PDH activity to no longer drop in response to H_2O_2 treatment (Fig. 3C). (We had similar data in our original submission, except they were obtained using overexpression of mutant DLAT in a wildtype background.) This

is an important result because it shows that these three cysteines mediate the effect of oxidative stress on PDH activity, and that the drop in PDH activity upon H_2O_2 treatment is a specific effect, and not simply damaging the cells.

4. One would expect that elevated PDH activity should cause more flux from glycolysis into the TCA cycle. Consistent with this, we find that cells reconstituted with C488S/C586S/C618S DLAT have increased respiration, assayed via a Seahorse assay (Suppl. Fig. 7E). As requested by the reviewer, these data show downstream functional consequences on metabolism which are consistent with the increased PDH activity in these cells.
5. It is well established that a reduction in metabolic flux at the lower end of the glycolytic pathway (for example, at the level of pyruvate kinase) leads to a rerouting of flux from glycolysis into the pentose phosphate pathway, thereby generating NADPH to combat oxidative stress (e.g. PMID 18154684, 26190262). In this context, the regulation of PDH by oxidative stress would make a lot of sense: it creates a homeostatic loop in which oxidative stress decreases PDH activity, consequently increasing flux through the pentose phosphate pathway and boosting NADPH production to counteract the stress. Consistent with this model, we find that cells expressing the C488S/C586S/C618S DLAT mutant - where PDH activity can no longer adjust to oxidative stress - exhibit lower NADPH levels (Fig. 4A) and reduced glutathione (Fig. 4B) in response to H_2O_2 compared to control cells.
6. Consistent with this, cells expressing the C488S/C586S/C618S DLAT mutant have impaired capacity to detoxify H_2O_2 (Suppl. Fig. 9 and Fig. 4C).
7. Consistent with this, cells expressing the C488S/C586S/C618S DLAT mutant have increase protein oxidation (Fig. 4D-E) and impaired survival (Fig. 4F-G) in response to an oxidative challenge.
8. Previous work has shown that cells with impaired capacity to fight oxidative stress are also impaired in tumor formation (PMID 22052977). Consistent with this, we find that cells expressing the C488S/C586S/C618S DLAT mutant form xenograft tumors more slowly than isogenic control cells (Fig. 4H).

Together, these data show the downstream consequences of DLAT oxidation on metabolism, on the oxidative stress response, and on tumor formation. We thank the reviewer for suggesting these experiments because we believe they have made the manuscript much stronger and more complete.

Major comments

1. Does all C to S DLAT mutation affect any cellular functions such as cell proliferation, migration, and/or adhesion in response to MG? MG is one of major metabolites associated with diabetes, liver dysfunction and atherosclerosis. It is important to examine if all C to S DLAT mutation affects any of these disease progressions (e.g. HFD-induced diabetes).

Although we happened to discover DLAT oxidation while studying MG, we think these observations are more relevant to oxidative stress. We do not want to claim that DLAT oxidation is the major downstream effector of MG on cell behavior. We have therefore rewritten the manuscript to reduce the focus on MG and diabetes and to focus instead on oxidative stress.

As detailed above, we now provide evidence that the triple DLAT mutation affects cellular metabolism (respiration), the redox status of the cell, the ability to fight and resist oxidative stress, and, as the reviewer suggested, cell proliferation in xenografts. We therefore provide in the revised manuscript the metabolic and physiological relevance that the reviewer is asking for here, but in the context of oxidative stress and tumor growth.

(That said, since diabetes patients have reduced PDH activity and reduced metabolic flexibility, it will be exciting in future studies to see if regulation of PDH by oxidation also plays a role in this context.)

2. In line with the comment above, MG is known to regulate cancer growth as well but with more complexity because low dose of MG promotes while high dose of MG inhibits tumor growth. Does all C to S DLAT mutation affect any tumor metabolism and growth in response to MG? The authors should test if all C to S DLAT mutant expression would affect TCA cycle, cancer cell proliferation, and tumor growth in mice in response to MG treatment if they would focus on cancer.

The reviewer had a good intuition. Indeed, as detailed above, the triple C to S DLAT mutation affects both respiration (Suppl. Fig. 7E) and tumor growth in mice (Fig. 4H).

Minor comments

3. It is unclear if C to S mutation on DLAT would affect basal activity of PDH. Such information would be helpful for readers to understand regulation of PDH by cysteine oxidation.

We now provide new data in Suppl. Figure 7C-D which show that the triple mutant C488S/C586S/C618S DLAT does not cause a decrease in PDH activity. In fact, if anything, as expected, it causes a mild increase in PDH activity, consistent with the fact that the oxidation of these three cysteines is inhibitory. (In order to control for batch effects, we separately generated two sets of DLAT knockout cells reconstituted with either WT or triple-mutant DLAT, which we call set #1 and #2 in Suppl. Fig. 7D-E).

In this context, we would like to point out that Reviewer 2 had doubts whether our PDH assay works correctly. We spent a lot of time at the beginning of the project to optimize our PDH activity assay and include, for instance, an LDH inhibitor in the assay mix to block LDH activity. Since we now have DLAT knockouts, we can prove that our assay is specific because the DLAT knockouts have no detectable PDH activity above background, Suppl. Fig. 7C.

Reviewer #2

This paper makes the provocative claim that PDH regulation by cysteine oxidation on the DLAT subunit (E2) supersedes the well-known regulation via E1-alpha phosphorylation. If **true, this would be a very important finding with great relevance.** The PDH complex sits at the critical junction between the Warburg effect and mitochondrial oxidative phosphorylation, and its regulation (whether activated or inactivated) determines the cellular source of acetyl-CoA. Overall, the paper is nicely written.

We thank the reviewer for these supportive comments. We also thank the reviewer for the constructive suggestions below, which led us to add a large amount of new data to the manuscript, which has significantly improved it.

We have added an entire new main Figure studying the downstream consequences of DLAT oxidation on cellular metabolism and phenotypes. When cells are exposed to oxidative stress, they execute a metabolic shift, blocking glycolytic flux into the TCA cycle to shunt glycolytic carbons toward the pentose phosphate pathway. This generates NADPH which cells use to detoxify H_2O_2 and to reduce oxidized cysteines. We find that DLAT oxidation is required for cells to mount an effective antioxidant response. Mutations preventing DLAT oxidation render PDH resistant to oxidative inhibition (new Fig. 3C), impair NADPH generation (new Fig. 4A), reduce peroxide detoxification (new Fig. 4C), and sensitize cells to oxidative damage (new Fig. 4D-E) and death (new Fig. 4F-G). We also performed in vivo experiments using xenografts to study the relevance of this mechanism. Cells in the core of a tumor are exposed to oxidative stress, and previous work has shown that cells with impaired capacity to fight oxidative stress are also impaired in tumor formation (PMID 22052977). To test this, we generated xenografts in mice with cells carrying either wildtype DLAT or TM DLAT. Although TM cells proliferate as quickly as WT cells in cell culture, the TM cells formed tumors more slowly than WT cells (new Fig. 4H). Given these data, showing relevance of DLAT oxidation within the context of oxidative stress, we have re-written the manuscript. We hope the reviewer agrees the manuscript is significantly improved.

Finally, we would like to point out that we rephrased the manuscript and removed statements that PDH regulation by oxidation is dominant over regulation by phosphorylation (although the data indicate this is the case) to not risk sounding dismissive towards previous work by others.

However, I do not feel the conclusions are sufficiently supported by the experiments and data presented. Most experiments lack important control and rigorous methodology. Significantly more rigorous and compelling data are needed to convincingly conclude that DLAT oxidation is a bona fide regulatory mechanism for PDH activity and flux.

1. Concerns About PDH Activity Measurement
I have major concerns regarding how PDH activity is measured. The assay used essentially monitors NAD-to-NADH conversion via an enzymatic kit. It requires the isolation of mitochondria and subsequent incubation with substrates for the PDH complex (including pyruvate and NADH). First, there is no confirmation of mitochondrial purity. It's extremely difficult to purify mitochondria free to cytosolic or other organelle contaminants. Even IP of mitochondria can pull down cytosolic lactate dehydrogenase (LDH) and other membrane organelles. Many cytosolic enzymes that also use NAD/NADH (e.g., LDH, which uses NAD and pyruvate) could co-purify with mitochondria.

We apologize for the confusion, because all of these questions raised by the reviewer are due to the fact that we did not explain well enough in the original manuscript the efforts we undertook at the beginning of the project to establish a robust assay for PDH activity. Indeed, as the reviewer points out, any contaminating LDH activity will interfere with our measurements because it can convert the pyruvate in the assay to lactate, thereby consuming NADH. For this reason, we include an LDH inhibitor (GNE-140) in all of our enzymatic assays (except for the HeLa data, Suppl. Fig. 3C-D, where we used a commercial kit). In the original submission, we mentioned this inhibitor in our Materials & Methods but didn't highlight it in the results section. We optimized this at the time by performing LDH activity assays (by adding lactate as the substrate instead of pyruvate) and we made sure that the concentration of LDH inhibitor is working efficiently (Reviewer Figure 1):

Reviewer Figure 1: 200µM GNE-140 inhibits LDH activity in cell extracts.

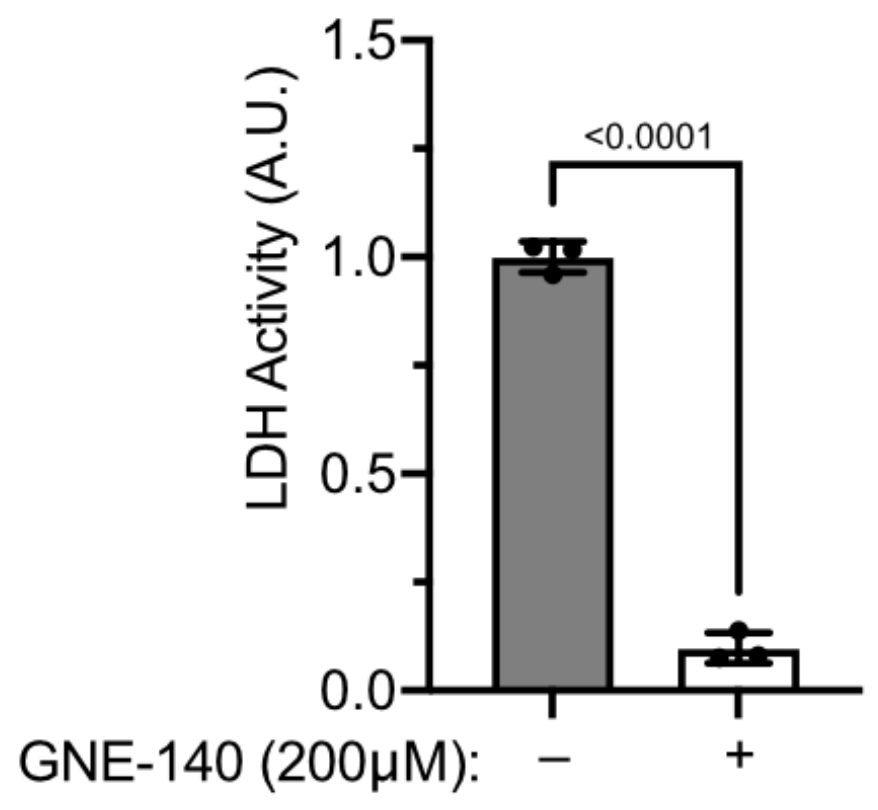

Indeed, this enables us to even measure PDH activity in whole cell lysates, but nonetheless for the data in this manuscript we purified mitochondria to ensure that we are measuring mitochondrial PDH activity. We now provide new data showing that our PDH activity assay is indeed specific. We were able to generate DLAT knockout cells, which surprisingly are viable (see western blot confirmation in Suppl. Fig. 7B). If we perform a PDH activity assay on these knockout cells, we obtain no detectable PDH activity above background (Suppl. Fig. 7C) proving that our assay is specific for PDH.

Second, the assay runs for at least 15-20 minutes, during which substantial changes in the mitochondrial content might occur.

After purifying mitochondria, we lyse the mitochondria for the activity assay. Hence no changes in mitochondrial content can occur during the measurement.

In addition, there is no clear normalization for the assay. For example, how are the PDH activities in GLO1^WT and GLO1 KO cells normalized when they come from different cell lines and distinct mitochondrial preparations?

We normalize all PDH activity measurements to the total amount of protein in the individual mitochondrial preps. This normalizes for differences in mitochondrial content between cells lines and between preps. (This was mentioned in the Methods and also in the labeling of the y-axis of the figures, but we now also mention this explicitly in the Results text to clarify this for the reader.)

In addition, the tracing data in Figure 1K lack important controls. Citrate M+2 tracing from U13C Glucose should be conducted over a time course (e.g., 2, 5, 10, 15 minutes) and normalized to pyruvate M+3 tracing if U-13C glucose is used as the tracer. The α-KG, succinate, and fumarate tracing results show very low percentage values (single-digit or even 0.1%), which raises questions about their accuracy. While 13C tracing

would indeed be a more reliable indicator of PDH activity, more rigorous execution and replication of these experiments are needed.

As suggested by the reviewer, we now normalize the citrate M+2 to pyruvate M+3 (Suppl. Fig. 3B). Consistent with the data showed in the original submission, this ratio shows that in Glo1^{KO} cells the conversion of pyruvate to citrate is reduced, which is a specific readout for PDH activity. In the case of citrate, the labeling percentage is 5% at 5 minutes of labeling, and 20% at 10 minutes of labeling, which are within a reliable range. This is the key result from the entire metabolic flux analysis because it specifically measures the conversion of pyruvate (the PDH substrate) to citrate (the most proximal TCA intermediate after acetyl-CoA).

We agree with the reviewer that the incorporation rates for fumarate are low, so we have removed these data from the manuscript because they are superfluous.

(For succinate and aKG, the incorporation rate is around 1%, but we feel uncomfortable removing them because the aKG data do not fit with the rest of the manuscript, and we would feel like we're hiding something. However, if the reviewer feels it is better to remove the succinate and aKG data because they are less reliable, we are happy to do so.)

To support the tracing experiments, we now also perform additional assays to show that PDH activity is reduced in the Glo1 knockouts, in addition to the PDH activity assays:

1. We perform respiration assays and find that oxygen consumption is reduced in the Glo1 knockouts compared to control cells (Fig 1G).
2. We see that the rate of acidification of the medium (a readout for lactate secretion) is increased (Fig 1J), in agreement with our original data showing that lactate secretion increases (Fig 1I).

Combined, this divergent effect on lactate secretion versus respiration agrees with our enzymatic assay showing reduced PDH activity.

In addition, we now also support these data with respiration assays on cells expressing a C488S/C586S/C618S triple mutant DLAT, and see that, as expected, respiration is increased (Suppl. Fig. 7E).

2. Correlational Nature of the Data

Much of the data is correlative. The characterization of GLO1-deficient cells and their MG levels is nicely done. However, the conclusion that GLO1 deficiency leads to increased mitochondrial redox stress, which then results in decreased PDH activity, is highly speculative. These conclusions rely primarily on

correlations between PDH activity (already in question) and treatments that raise or lower mitochondrial ROS.

To answer this concern, we would like to break down the chain of causality mentioned by the reviewer into 3 steps, and provide new data as evidence for each one:

1. Glo1 deficiency or elevated MG causes mitochondrial redox stress
2. This leads to DLAT oxidation on Cys488, Cys586 and Cys618
3. Oxidation of DLAT on these three sites causes reduced PDH activity

1. Glo1 deficiency causes elevated mitochondrial redox stress

We did not delve much into this issue in the original submission because a number of studies in the published literature show that elevated MG causes oxidative stress in a variety of cell types:

• PMID 41049288 ("Methylglyoxal induces oxidative stress and ferroptosis of renal tubular epithelial cells in acute and chronic kidney injury mice"): shows that in HK-2 renal tubular epithelial cells MG treatment "increased intracellular reactive oxygen species (ROS) levels ... and decreased glutathione (GSH) levels"
• PMID 25343013 shows that treatment of cells with MG "promoted the production of reactive oxygen species (ROS) and depleted glutathione (GSH) content" in HepG2 cells
• PMID 11897769 concludes that "methylglyoxal increases oxidative stress" in vascular smooth muscle cells
• PMID 24040147 shows that prostate cancer cells with a Glo1 allele variant with low activity have elevated ROS and lower levels of reduced glutathione
• PMID 27956549 shows that Glo1 knockout Schwann cells have elevated levels of cysteine nitrosylation
• PMID 17487230 "Methylglyoxal, oxidative stress, and hypertension". This review discusses how elevated MG leads to ROS production.

We now provide several pieces of data that show this is indeed the case in our system as well. Suppl. Figure 2D shows that addition of MG to cells for 1 hour increases ROS levels, measured using DCFDA. This dye measures the levels of hydrogen peroxide and hydroxyl radicals, all of which oxidize proteins. These data complement the data in Suppl. Fig. 1F which show elevated ROS in Glo1 KO cells. We now also show in Suppl. Fig. 2E-F that upon treatment of cells with MG, hyperoxidation of PRDX3 increases - a typical marker for oxidative stress. These data are not correlative, but experiments where we specifically perturb the system in a controlled way to increase MG, and then measure the consequences.

2. This leads to DLAT oxidation on Cys488, Cys586 and Cys618

• Fig. 2G and Suppl. Fig. 6B show that Glo1 knockouts as well as MG treatment increase DLAT oxidation by directly assaying DTT-sensitive oxidation on DLAT.

• Fig. 2H shows that elevation of oxidative stress via treatment with H_2O_2 increases DLAT oxidation by directly assaying DTT-sensitive oxidation on DLAT.
• Fig. 2E shows that if we counteract endogenous oxidative stress by treating cells with Trolox, this reduces DLAT oxidation.
• Fig. 3B and Suppl. Fig. 6D show that this oxidation occurs on Cys488, Cys586 and Cys618
• In Fig. 2E, we experimentally reverse the reduced activity of PDH in Glo1KO cells by treating them with the anti-oxidant Trolox. This proves epistatically that the reason why Glo1 KO cells have reduced PDH activity is increased oxidation.

Also these experiments are not correlative, but controlled, perturbation experiments where we specifically manipulate one variable (e.g. adding MG or H_2O_2) and then measure the outcome of DLAT oxidation.

<u>3. Oxidation of DLAT on these three sites causes reduced PDH activity</u>
We now provide new data which are not correlative, but targeted perturbation experiments, which convincingly show that oxidation of Cys488, Cys586 and Cys618 causes reduced PDH activity.
• We generated DLAT knockout cells (which surprisingly are viable, see western blot confirmation in Suppl. Fig. 7B). We then stably reconstituted these knockouts with either wildtype DLAT or mutant DLAT where Cys488, Cys586 and Cys618 are mutated to serine and therefore cannot be oxidized.
• We then tested the effect of these mutations on PDH activity. As expected, mutation of these three cysteines to serine does not impair PDH activity. In fact, if anything, it increases baseline PDH activity (Suppl. Fig. 7C-D). This is expected because oxidation on these cysteines reduces PDH activity (Fig. 2) and there is some baseline oxidation on these cysteines in the absence of oxidative stress in cell lines in culture (Fig. 2G), in vivo in humans (Fig. 6) and in vivo in mice (Fig. 5).
• Mutation of these three cysteines causes PDH activity to no longer drop in response to H_2O_2 treatment (Fig. 3C). This is an important result because it shows that these three cysteines mediate the effect of oxidative stress on PDH activity.

Similarly, there is no direct evidence that cysteine oxidation of DLAT at the three serine residues actually regulates PDH activity or flux. Demonstrating this would require a detailed characterization of DLAT oxidation biochemically or genetically (see below) and its manipulation, followed by rigorous PDH activity assay.

As suggested by the reviewer, and detailed below, we now provide new genetic data in Suppl. Fig. 7 and Fig 3C showing that mutation of DLAT Cys488, Cys586 and Cys618 to serine, which abolishes the oxidation on these sites, causes PDH activity to become resistant to oxidative stress. Hence, the drop in PDH activity caused by oxidative stress is due to oxidation of these three cysteines. (Please note that mutation

of these three sites to serine does not impair PDH activity in any way - indeed, it increases its activity, consistent with the fact that DLAT has some basal oxidation on these sites, as observed by our PEG exchange assay in Fig. 2G).

In addition, PDH activity is regulated acutely (within seconds to minutes). Most of the authors treatment (i.e. GLO1 KO or drug treatment) spans hours to days, during which time the cells would have had time to undergo other secondary changes.

We agree with the reviewer that the Glo1KO cells are chronically mutant, and hence they have elevated MG levels for hours and days. However, we also confirmed all of the key results with treatments done for only 60 minutes:

- The fact that DLAT becomes oxidized on Cys488, Cys586 and Cys618 was done with a 60 minute treatment of H_2O_2 (Fig. 2G for shifting of WT protein, and Fig. 3A-B for shift of the mutants).
- Likewise, we also see a decrease in DLAT oxidation when cells are treated with Trolox for 60 minutes, to reduce endogenous ROS (Fig 2I).
- We see reduced DLAT activity when cells are treated with H_2O_2 for 60 minutes (Fig 2D).
- We see that Trolox re-activates PDH activity in Glo1 KO within 60 minutes (Fig. 2E).
- We see that DLAT activity is rescued upon a 60-minute H_2O_2 treatment if Cys488, Cys586 and Cys618 are mutated to serine (Fig. 3C).

Furthermore, we now provide new data showing that the activity of purified PDH decreases if it is treated with H_2O_2 in vitro (Suppl. Fig. 5C) excluding any possible secondary changes.

3. Weak Characterization of DLAT Oxidation

The characterization of DLAT oxidation is weak both biochemically and genetically. Biochemically, the authors should employ more established methods for studying DLAT disulfide bond formation, such as mass spectrometry or chromatography, **or perform assays with purified DLAT protein.** Genetically, the authors should generate DLAT knockout cells (rather than relying on cells expressing endogenous WT DLAT) and perform mutagenesis studies to assess the functional consequences of specific cysteine oxidations.

There are essentially two mass spectrometry methods used to identify cysteine oxidation. The most cutting-edge method is to isolate the protein of interest in non-reducing conditions, trypsinize it, and then identify the disulfide-bonded peptides based on their fragmentation spectra. We tried this method, but unfortunately the cysteines of interest were not observable, perhaps because they don't 'fly well'.

The more standard mass spectrometry method is to trypsinize a protein, treat half of the sample with TCEP to reduce the disulfide bond, leading to a +2 Da mass shift, and then to compare the masses of the two samples by mass spectrometry. The method we are using is conceptually exactly the same, except that we are inducing a larger mass shift, so that we can observe it on an SDS-PAGE gel. It is true that the mass spectrometer can identify the site that has the mass shift, but we combined our method with point mutagenesis to successfully identify the oxidized sites. Hence the method we are using is essentially the same as the mass spec method.

In addition, we are using band-shifting to show that DLAT migrates as a trimer under non-reducing conditions compared to reducing conditions (Fig. 3D-F), and would like to point out that this is a standard method to show disulfide bonding (e.g. PMID 29091273).

We have now done the genetic experiments suggested by the reviewer:

1. We generated DLAT knockout cells (which surprisingly are viable). These cells have no DLAT protein (Suppl. Fig. 7B) and no PDH activity (Suppl. Fig. 7C). As expected, these cells are highly glycolytic, and acidify the culture medium extremely quickly. We then stably reconstituted these knockouts with either wildtype DLAT or mutant DLAT where Cys488, Cys586 and Cys618 are mutated to serine and therefore cannot be oxidized (Suppl. Fig. 7B).
2. We then tested the effect of these mutations on PDH activity. As expected, mutation of all three cysteines to serine ("TM") does not impair PDH activity. In fact, if anything, it increases baseline PDH activity (Suppl. Fig. 7C-D). This is expected because oxidation on these cysteines reduces PDH activity (Fig. 2) and there is some baseline oxidation on these cysteines in the absence of oxidative stress in cell lines in culture (Fig. 2G), in vivo in humans (Fig. 6A,E) and in vivo in mice (Fig. 5E-G).
3. Consistent with the data in our original submission, mutation of these three cysteines causes PDH activity to no longer drop in response to H_2O_2 treatment (Fig. 3C). (We had similar data in our original submission, except they were generated using overexpression of mutant DLAT in a wildtype background.) There is a partial contribution from mutation of each of the three cysteins, with the triple-mutant (TM) showing the best rescue.

Together, these data prove that the reduced PDH activity caused by oxidative stress is specifically due to oxidation of these three cysteines, and no other secondary or non-specific effects.

Furthermore, as suggested, we performed assays using purified PDH protein. We find that incubation of purified PDH with H_2O_2 in vitro causes reduced PDH activity (Suppl. Fig. 5C) whereas incubation with MG does not (Suppl. Fig. 5D), supporting our original data that the effect of H_2O_2 on PDH is direct, whereas the effect of MG is not.

Reviewer #3

The paper explores PDH regulation and this is an interesting area as 4 of the 5 kinases within mitochondria regulate it. PDH integrates a wide range of metabolic signals and is a key node in cancer. While the work is explores and interesting area I have a number of significant concerns.

We thank the reviewer for pointing out the interest in our manuscript. We have now added a large amount of new data in response to the reviewer's comments, which we believe has made the manuscript significantly stronger.

Furthermore, we also improved the manuscript by toning down the comparison between the effects of oxidation versus phosphorylation on PDH activity - we have completely removed claims that oxidation is dominant over other mechanisms that have been previously published because we simply want to make the point that oxidation of PDH is one important regulatory mechanism, and we don't want to risk sounding dismissive of the contribution of other mechanisms to PDH regulation. We feel that, while trying to convince editors about the importance of our findings, we had not hit the right tone in the original submission, so we thank the reviewer for pointing this out.

1 The authors use the term ROS as if its one chemical with one reactivity and one type of target. They also move between MG and H2O2 as if they are equivalent when the former irreversibly alkylates thiols and amines and the latter reversibly oxidises cysteines. The authors should rewrite the paper so that it makes less use of fuzzy concepts like ROS and more use of the specific chemistry the authors believe is occurring.

We have now re-written the manuscript to:
1. use more specific terms for the chemistry
2. clarify the link between MG and oxidative stress (please see below).

2 Currently the MG and H2O2 parts of the paper are not well linked. Its not clear to this reviewer why MG which is a glycolysis breakdown product from upstream glucose should have the same regulatory outcome as H2O2 which probably forms from downstream O2. The manuscript suggests MG acts via upregulation of H2O2 by an unidentified mechanism. The rationale for moving to H2O2 seems to be increased DCFDA signal from MG, but DCFDA is not a clean tool and it isn't compartmentalised. The authors should prove this link by showing that MG causes increased mitochondrial H2O2/O2.- or shifts the redox state in the

vicinity of PDH by measuring peroxiredoxin 3 dimerisation and/or aconitase inactivation.

We apologize for not going into much detail in our original submission regarding the link between MG and oxidative stress, which was due to the fact that this is a fairly well established connection in the MG literature. Multiple papers and reviews point out that elevated MG leads to oxidative stress (e.g. PMID 41049288, 25343013, 11897769, 24040147, 27956549, 17487230). To make the manuscript understandable to a non-MG audience, we now explain this link explicitly in the introduction. We thank the reviewer for pointing this out. (One explanation in the literature is that MG detoxification involves reduced glutathione in a catalytic cycle; therefore, a higher MG detoxification flux engages a larger proportion of glutathione in this process. Furthermore, MG impairs expression and function of antioxidant enzymes.)

In addition, as suggested by the reviewer, we now provide data showing that elevated MG leads to increased PRDX3 hyperoxidation, using an antibody that specifically detects it (Suppl. Fig. 2E-F). This proves that indeed MG treatment causes elevated oxidative stress within mitochondria.

Please note that we also assessed PRDX3 dimerization via band shifting on an SDS gel, as suggested by the reviewer, however, both H_2O_2 treatment and MG treatment cause such a large increase in PRDX3 oxidation that the amount of dimer decreases rather than increasing (Reviewer Fig. 2). This is due to oxidation of the relevant cysteines to sulfonic acid, as detected by the PRDX3 hyperoxidation antibody (Suppl. Fig. 2E-F), which cannot participate in disulfide bonds. This has already been reported in the literature (PMID 19356151).

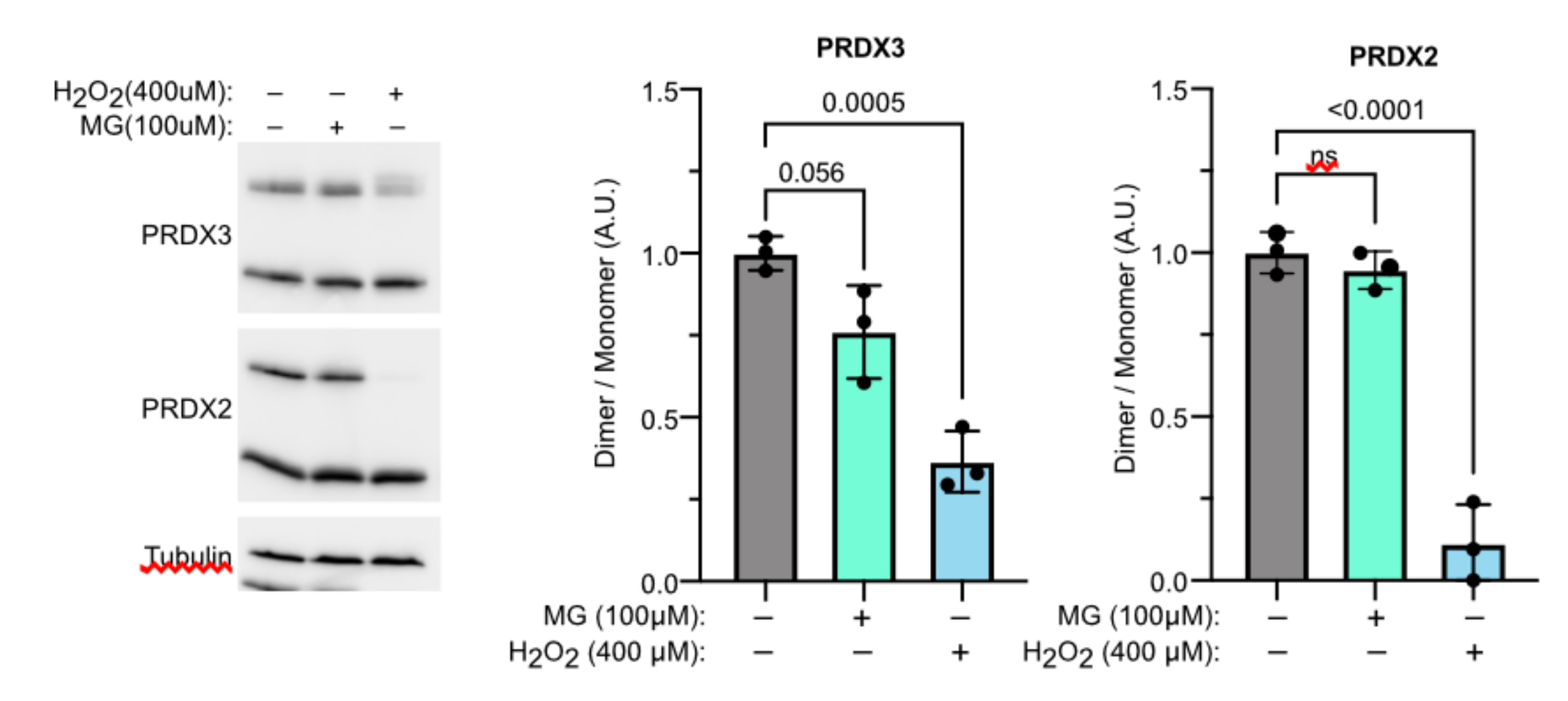

Reviewer Figure 2: Both H_2O_2 and MG treatment cause decreased dimerization of PRDX2 and PRDX3.

3 The 400 uM H2O2 concentrations used here and long incubation times might harm/kill HEK cells. Many processes could be negatively and non-specifically affected under these harsh and unlikely to be physiological conditions. More needs to be done to show that the loss of PDH activity under these settings is a specific reversible regulatory response. This is particularly true since lower H2O2 concentrations (100-200 μM for 5 min) in the same cell lines have the opposite affect via oxidation of thiols on PDHK2 (doi: 10.1074/jbc.M112.400002, doi: 10.1074/jbc.M703591200).

We now provide several pieces of new data that show that the effect of H_2O_2 on PDH activity is specific and reversible, and not due to the treatment being non-specifically toxic.

Before explaining these data showing specificity and reversibility of the effect, we would like to provide some background regarding our H_2O_2 treatments. Unlike treatment of cells with other compounds (e.g. drugs), cells are able to detoxify H_2O_2 extremely rapidly. In new Suppl. Fig. 9, we now provide data showing that if we add 400μM H_2O_2 into a 6-well plate with cells, the concentration of H_2O_2 in the medium drops to less than <20μM within 30 minutes, corresponding to >95% detoxification (Suppl. Fig. 9B). (This does not happen if we add the H_2O_2 into a well lacking cells, showing it is not due to the intrinsic instability of hydrogen peroxide (Suppl. Fig. 9A)). This means that the <u>absolute amount</u> of H_2O_2 in a well is relevant, and not only the H_2O_2 concentration, because the absolute amount will determine the time needed for the cells to detoxify the H_2O_2. A given number of cells in a well will take longer to detoxify 3mL of 200μM H_2O_2 compared to 1mL of the same 200μM H_2O_2. If the cells take longer to detoxify the H_2O_2, then the cellular proteins will be exposed to the H_2O_2 for a longer period of time and accumulate more damage. Likewise, if we double the number of cells in a well, they will detoxify the H_2O_2 twice as fast, reducing the time their proteins are exposed to H_2O_2. Hence the relationship between the H_2O_2 concentration, the volume of medium in a well, and the number of cells in the well will determine how harsh the treatment is, and not simply the H_2O_2 concentration. This is consistent with work published by others, e.g. PMID 20673847 where they conclude "Taken together, our results indicate that the cytotoxic potency of H_2O_2 administered to cultured cells as a bolus is characterized by the AUC, which depends on its initial concentration, the ability of the cells to eliminate H_2O_2, and the cell concentration.", or PMID 3597544 which shows in Fig 1 that the cytotoxicity of H_2O_2 is cell-density dependent.

In our hands, a 400μM H_2O_2 treatment in a 6-well dish is equivalent to a 100-200μM H_2O_2 treatment in a 96-well plate. As an example, please see Reviewer Figure 3 below where we treated cells with varying concentrations of H_2O_2 in either a 96-well or a 6-

well format, and then quantified the number of viable cells after 24 hours. This shows that in a 96-well format (e.g. used in SeaHorse assays referenced above by the reviewer) 200μM H_2O_2 is as harsh as 400μM H_2O_2 in a 6-well plate. For this reason, we use in the manuscript 200μM H_2O_2 when we perform assays in 96-well plates, and 400μM H_2O_2 for assays in 6-well plates. In all cases, we provide only a single pulse of H_2O_2 and then wait 60 minutes before doing the assay.

Reviewer Figure 3: Cells were treated with varying concentrations of H_2O_2 in either a 96-well plate or a 6-well plate and cell viability was then assayed after 24h

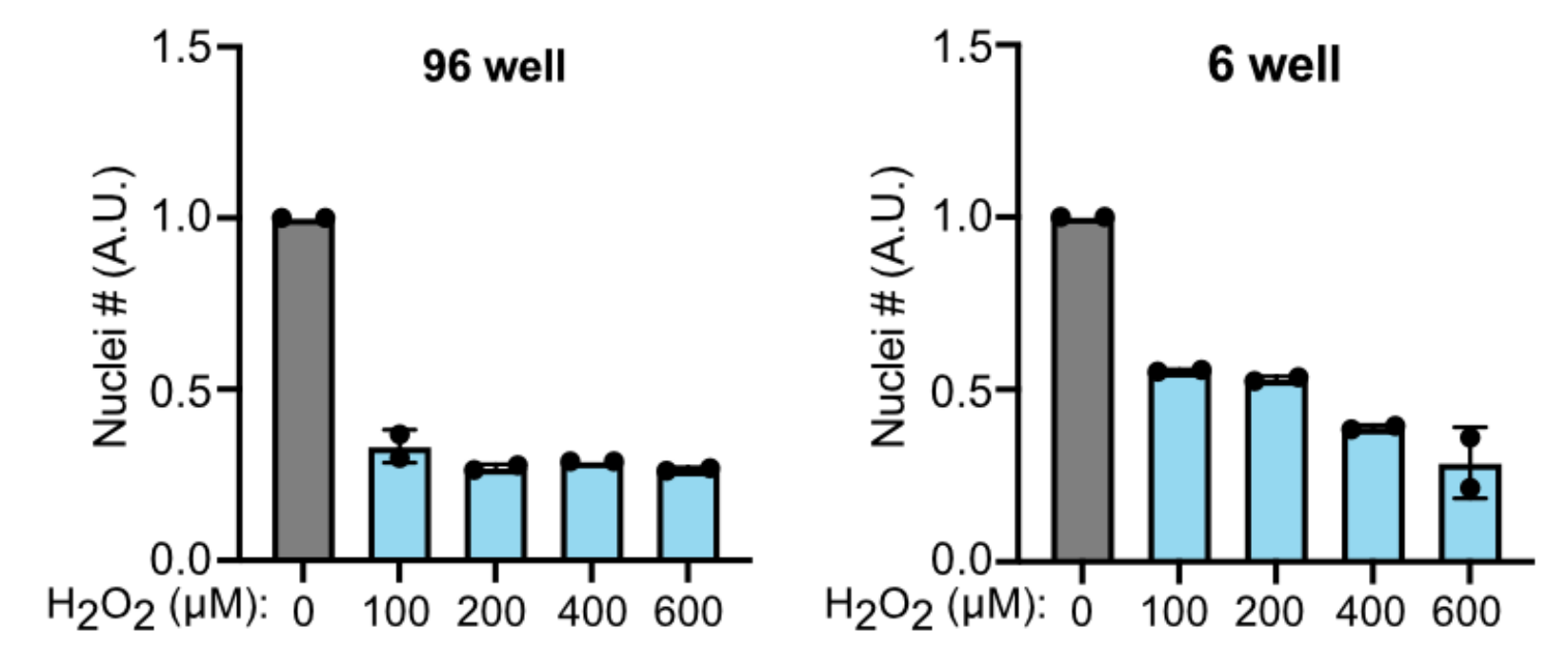

We now provide new data showing that the effect of H_2O_2 on PDH is reversible and specific:

• Supplementary Figure 5B shows that if we treat cells with 400μM H_2O_2 (in a 6-well format), then PDH activity drops at 30 and 60 min after the H_2O_2 pulse, but fully recovers by 120 min after the pulse. These results are consistent with the kinetics of H_2O_2 detoxification shown in Suppl. Fig. 9, and show that the effect on PDH is fully reversible, and not 'permanent damage' to the protein.

• Importantly, we now provide new data showing that PDH activity no longer drops upon H_2O_2 treatment if we mutate three cysteines on DLAT to serine (C488S/C586S/C618S) (Fig. 3C). This shows that the effect of H_2O_2 is specific to oxidation of these three cysteines and not due to harming or killing the cells or other non-specific effects.

The question remains, as the reviewer points out, why the previous publication Hurd et al. cited by the reviewer sees an opposite effect ? One possibility is that this due to lower H_2O_2 concentrations. To test this, we treated HEK293T cells with varying concentrations of H_2O_2 in a 6-well format and then measured PDH activity (Suppl. Fig. 5A). We see that 100μM and 200μM H_2O_2, as used in Hurd et al., cause a drop in PDH activity, not an increase, if we measure PDH activity directly with an enzymatic assay, consistent with our findings using 400μM H_2O_2. In Hurd et al., however, PDH enzymatic activity was not measured directly using an enzymatic assay, but indirectly via oxygen consumption. Oxygen consumption reflects the combined respiration of three fuel sources: 1) glucose via glycolysis and PDH, 2) glutamine via glutaminolysis, and 3) fatty acids via beta-oxidation. Since H_2O_2 can have multiple effects in a cell, we considered the possibility that the increase in oxygen consumption observed upon H_2O_2 treatment could be due to increased usage of glutamine or fatty acids, given that PDH activity is dropping. We therefore first measured oxygen consumption in cells

treated with 200µM H_2O_2 and we were able to reproduce the finding from Hurd et al. that oxygen consumption increases (Suppl. Fig. 5F, left panel). Thus 200µM H_2O_2 causes PDH activity to drop (Suppl. Fig. 5A) but oxygen consumption to increase. We then tested the effect of a fatty acid beta-oxidation inhibitor and a glutaminolysis inhibitor (BPTES) and found that in the presence of BPTES oxygen consumption no longer increases in response to H_2O_2 (Suppl. Fig. 5F, right panel), indicating that the increased oxygen consumption is due to glutaminolysis, not PDH activity. (Please note that the data from the left panel and right panel in Suppl. Fig. 5F come from the same SeaHorse plate, but we separated the graphs to make them visible, otherwise the curves would overlap each other.) Indeed, in the presence of BPTES where glutaminolysis is inhibited, H_2O_2 now causes a drop in oxygen consumption (Suppl. Fig. 5F, right panel), as expected by its inhibition of PDH.

4 The authors conclude that PDH oxidation dominates phosphorylation. The authors should titrate H2O2 into their system and measure PDH phosphorylation and activity to show the H2O2 concentration range this is true for. Furthermore they should show that loss in PDH activity is reversible after an hour of H2O2 exposure if the oxidant is removed.

If we understand correctly, the reviewer raises an interesting hypothesis, that perhaps at lower H_2O_2 concentrations the effect on PDKs dominates whereas at higher H_2O_2 concentrations the oxidative effect might dominate. As suggested by the reviewer, we now tested a range of H_2O_2 concentrations on PDH activity, but did not find a concentration that causes PDH activity to increase instead of decrease (Suppl. Fig. 5A).

Nonetheless, as discussed above, we removed our statements that the effect of oxidation dominates over phosphorylation because we do not want to sound dismissive of the regulation imparted by phosphorylation.

New Suppl. Fig. 5B shows that the drop in PDH activity is reversible.

5 Finally, I have concerns about exploring PDH regulation solely in highly glycolytic transformed cell lines as the results may have limited applicability. The paper would benefit from using fresh isolated heart mitochondria and showing that MG and much lower H2O2 concentrations (~10 uM as there is no cytosolic GSH/GPerox system to pass through) can cause similar PDH regulation.

Please note that HEK293T cells are not transformed.

Nonetheless, we can break down this issue into two questions. The first is whether oxidation of DLAT leads to reduced PDH activity. The second, is whether DLAT oxidation can be observed in a physiological context.

Regarding the first, there is no reason to think that cell immortalization or transformation can affect the mechanistic consequences of DLAT oxidation on PDH activity. Indeed, we even see this effect in vitro when purified PDH is treated with H_2O_2 (Suppl. Fig. 5C-D).

Regarding the second, it could indeed be possible, as the reviewer suggests, that the level of DLAT oxidation is different in immortalized cells versus primary cells. For this reason, we provide data from human liver samples (Fig. 6A), human muscle samples (Fig. 6E) and multiple different mouse tissues (Fig. 5G), all showing that DLAT is indeed highly oxidized *in vivo*, without needing to add any H_2O_2.

Reviewer #1 (Remarks to the Author):

The authors adequately addressed this reviewer's concerns using triple C to S mutant of DLAT. Just one minor concerns is that the Y -axis of tumor growth curve in Fig. 4H might be cm3 not mm3. Also it is recommended to show tumor weights from the xenograft study in Fig. 4H.

Indeed ! Thanks for catching that ! The units on the y-axis of Fig. 4H should have been cm3, not mm3. We have now fixed that.

Regarding the tumor weights, our Xenograft Core Facility which did the experiment said that the tumors became strongly attached to the skin. As a result, the final weight measurements are not accurate and show no significant difference. That said, as can be seen in Fig. 4H, the main difference lies in the rate of growth of the tumors before they plateau.

We thank the reviewer for the supportive comments throughout the review process, which helped make the manuscript stronger.

Reviewer #2 (Remarks to the Author):

I appreciate the authors' extensive efforts in addressing my concerns and the new data. As indicated in the title: "Regulation of PDH via cysteine oxidation on DLAT" and the abstract, the KEY message and novelty is the discovery of a new PDH activity regulation via cysteine oxidation. This really ought to be the emphasis of the paper and need to be more strongly supported. Without definitively showing this to be true, the remaining physiological/in vivo experiments do not have sufficient scientific rationale. I understand that other reviewers requested more physiological/in vivo relevance that are in figures 4-6, but the most significant finding in my opinion is that PDH complex can be regulated by DLAT (E2) oxidation despite many decades of studies on E1alpha phosphorylation.

To this end, with the exception of 1 cell-based 13C tracing figure (supplemental figure 3, which uses only Glo KO instead of direct DLAT oxidation manipulation), the authors' PDH activity assays have relied extensively on an in vitro PDH activity assay that is not a standard. I understand there are technical limitations in other aspects, however, U13C glucose tracing as the authors have done in the paper, is easily doable and a much more robust, unbiased, and physiological readout of PDH activity in intact cells.

We thank the reviewer for his/her comments. As detailed below, we have done the requested U13C glucose tracing experiments (which took a while because our

metabolomics core facility relocated to another buildings the past month) and we obtained the expected results that are consistent with the rest of the manuscript.

We would like to point out that the biochemical enzymatic assay and the U13C tracing experiment complement each other - i.e. the tracing approach also has drawbacks. The rate at which glycolytic pyruvate is converted to citrate (and hence the ratio of Cit m+2/Pyr m+3 measured by the tracing experiment) is influenced by the rate of pyruvate transport into mitochondria (which is affected by levels of the Mitochondrial Pyruvate Carrier), and by activity levels of citrate synthase, which condenses oxaloacetate with the acetyl-CoA coming from PDH to generate citrate:

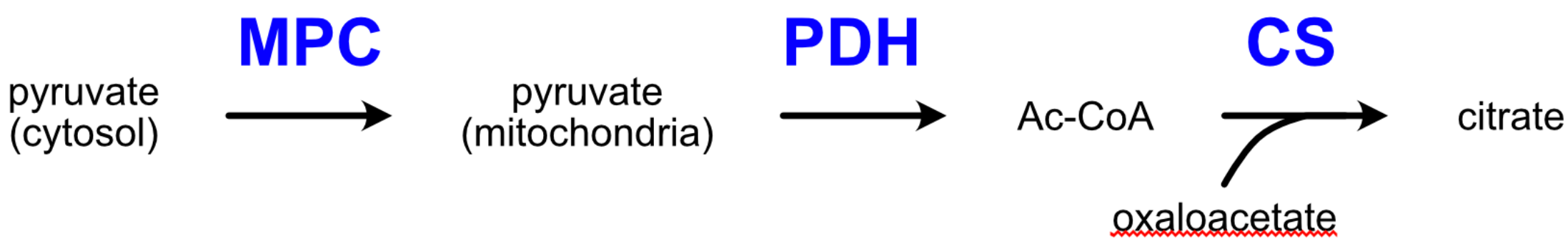

Hence the metabolic tracing method measures the combined activity of these three complexes. In contrast, the biochemical assay on PDH is the standard for measuring specifically PDH activity. Thus the two approaches complement each other nicely.

```
For me to be convinced of the central conclusion, I would really need to see the following two U13C tracing experiments, which should not take more than 1-2 weeks and for which the authors have already have all the cell lines/reagents/demonstrated capability:
1. U13C glucose tracing in H2O2 treated cells - the authors results should predict a decrease in Cit m+2/Pyr m+3 with H2O2 due to suppressed PDH
```

We have now done this experiment, and, as predicted by the reviewer, the Cit m+2/Pyr m+3 ratio drops significantly upon treatment with H2O2 (figure below, now included as Suppl. Fig. 5.) The experiment was done with exactly the same conditions as the PDH biochemical assay in main Figure 2D to be directly comparable.

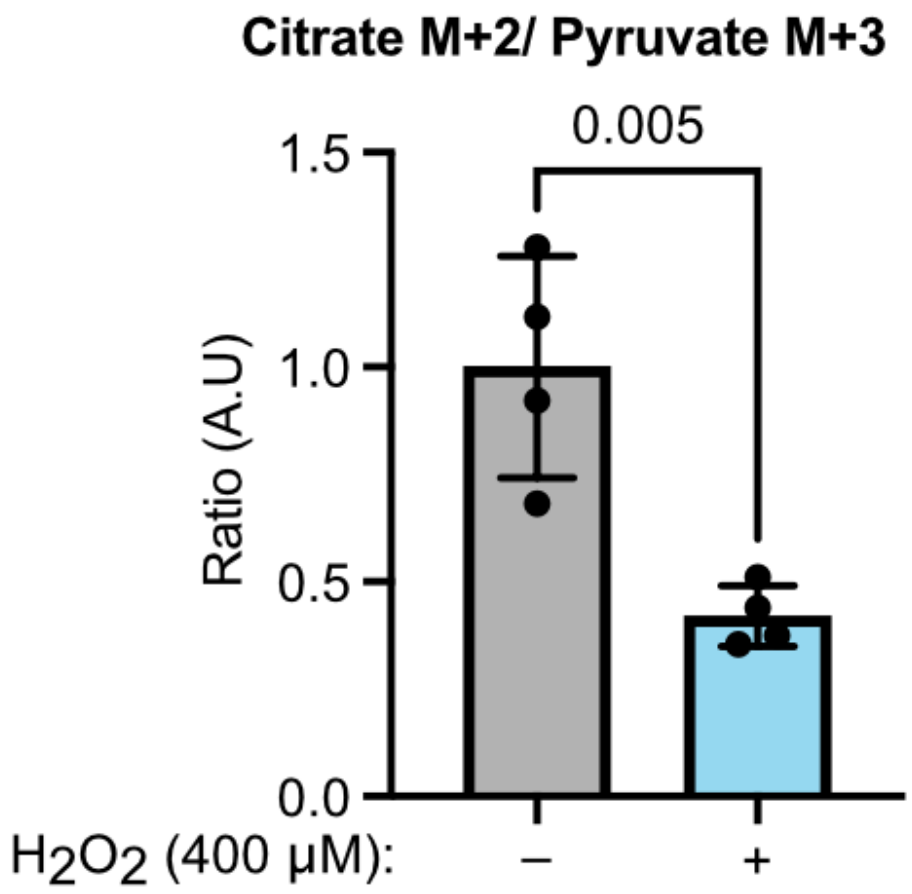

These results are consistent with our biochemical PDH activity assays, thereby strengthening the findings. They show that flux from pyruvate to citrate does not increase upon H2O2 treatment, as would be expected from the drop in PDH phosphorylation caused by H2O2 (Suppl. Fig. 8A), but decreases, in accordance with the PDH biochemical activity assays.

2. U13C glucose tracing in DLAT KO cells reconstituted with WT or C-S mutant DLAT - the authors results would predict an increase in Cit m+2/Pyr m+3 in the C-S mutant.

As requested, we have done this experiment (figure below) comparing the Cit m+2/Pyr m+3 ratio in DLAT KO cells reconstituted with wildtype DLAT ("WT") or the triple C-S mutant ("TM"). This shows the expected mild but not significant increase in the TM that is entirely in line with the biochemical assay shown in main Fig. 3C (column 1 vs 11).

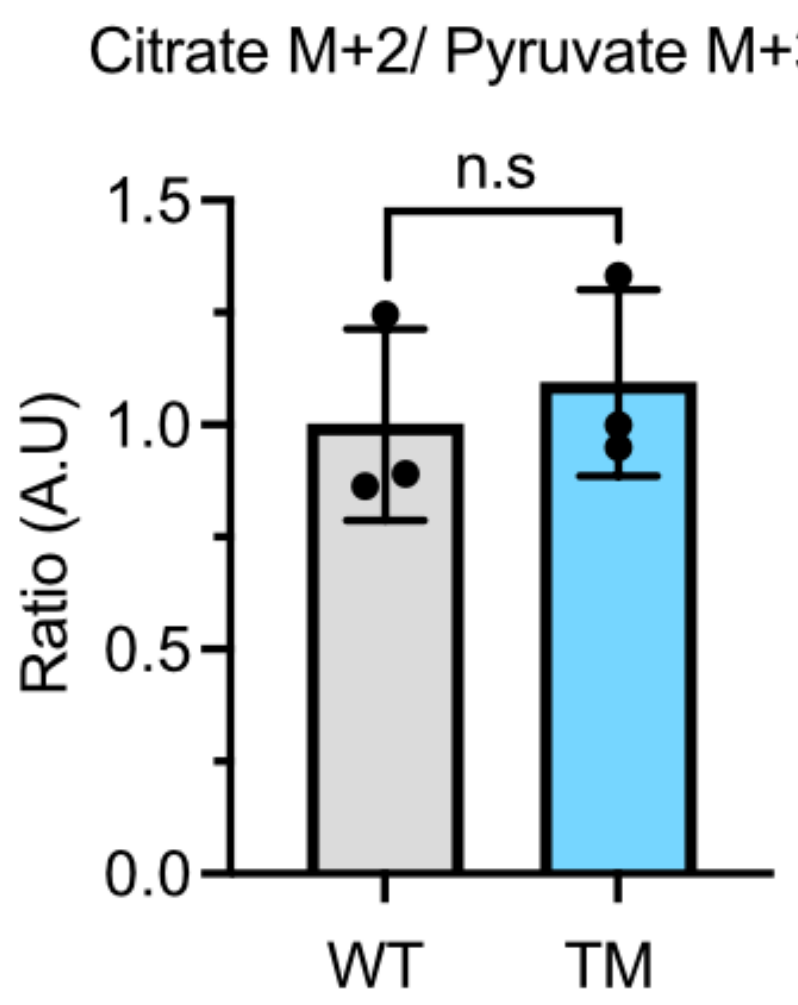

I also encourage the author to move supplemental figure 3B into the main paper as U13C tracing is a much more widely accepted and physiologic readout of PDH activity in cells. I would encourage authors to remove Succinate and aKG as the readout of PDH activity is a ratio of citrate m+2/pyruvate m+3.

As suggested, we have moved Suppl. Fig. 3B into main Figure 1 (now Fig. 1H) and we removed the aKG data. We kept the succinate data because they were clean and statistically significant in Suppl. Fig. 3A, not as a readout for PDH activity (which we agree with the reviewer, they are not), but as a readout for a metabolic effect further downstream in the TCA cycle, which we now made more clear in the main text.
