# Supplementary material for: Regulation of PDH via cysteine oxidation on DLAT
Source: Nat Commun. 2026 Oct 14;17:10784. doi: 10.1038/s41467-026-78266-6 (PMC13669365; doi:10.1038/s41467-026-78266-6)
Supplement: Supplementary file 5 [file 41467_2026_78266_MOESM8_ESM.zip › Uncropped Blots Figure 2.pdf]

Figure 2

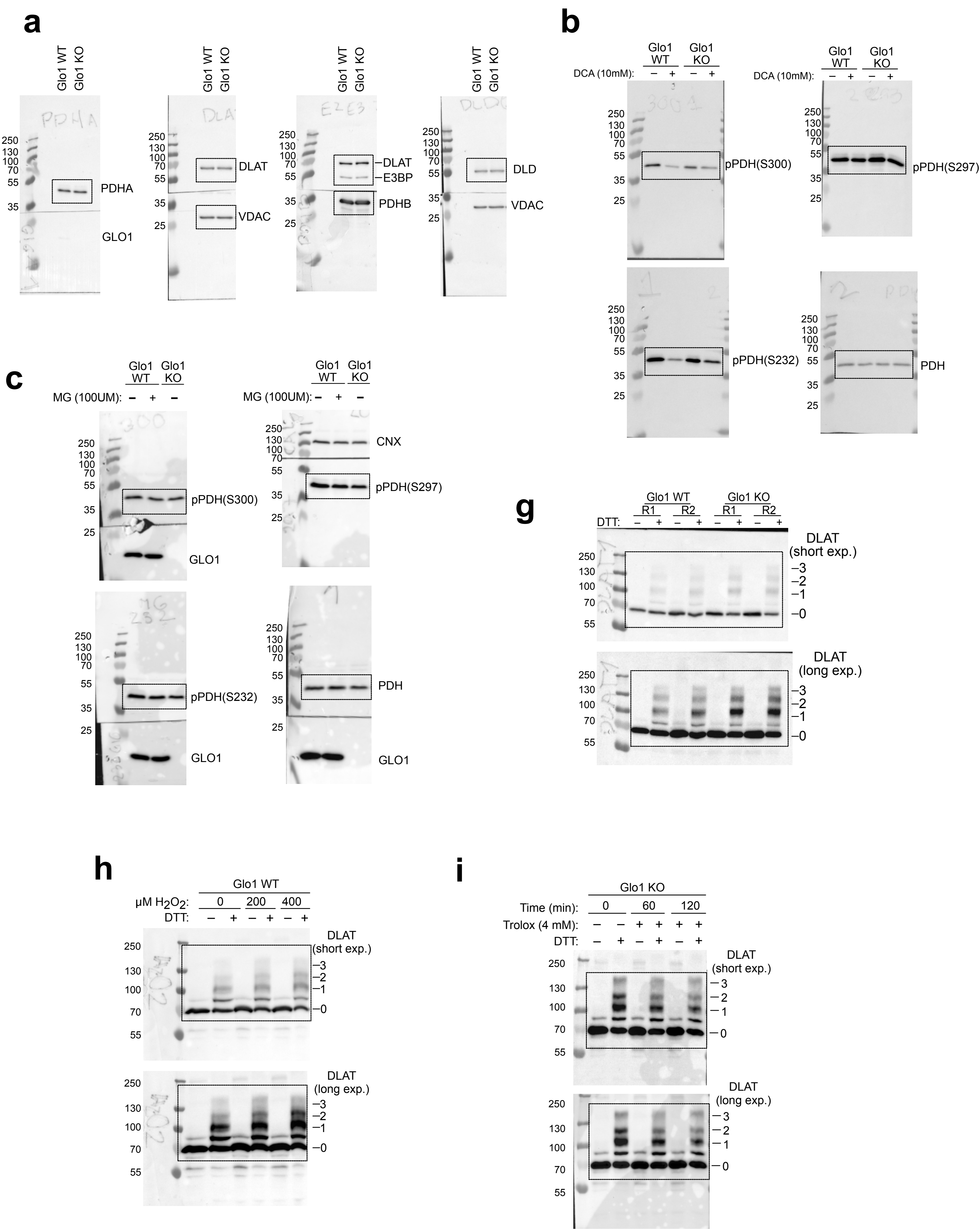
a
Glo1 WT
Glo1 KO
PDHA
GLO1
DLAT
VDAC
DLAT
E3BP
PDHB
DLD
VDAC
b
Glo1 WT
Glo1 KO
DCA (10mM):
pPDH(S300)
pPDH(S297)
pPDH(S232)
PDH
c
MG (100UM):
pPDH(S300)
GLO1
CNX
pPDH(S297)
pPDH(S232)
PDH
g
Glo1 WT
Glo1 KO
R1
R2
DTT:
DLAT (short exp.)
DLAT (long exp.)
h
Glo1 WT
μM H2O2:
0
200
400
DTT:
DLAT (short exp.)
DLAT (long exp.)
i
Glo1 KO
Time (min):
0
60
120
Trolox (4 mM):
DTT:
DLAT (short exp.)
DLAT (long exp.)
