# Supplementary material for: Regulation of PDH via cysteine oxidation on DLAT
Source: Nat Commun. 2026 Oct 14;17:10784. doi: 10.1038/s41467-026-78266-6 (PMC13669365; doi:10.1038/s41467-026-78266-6)
Supplement: Supplementary file 5 [file 41467_2026_78266_MOESM8_ESM.zip › Uncropped Blots Figure 3.pdf]

Figure 3

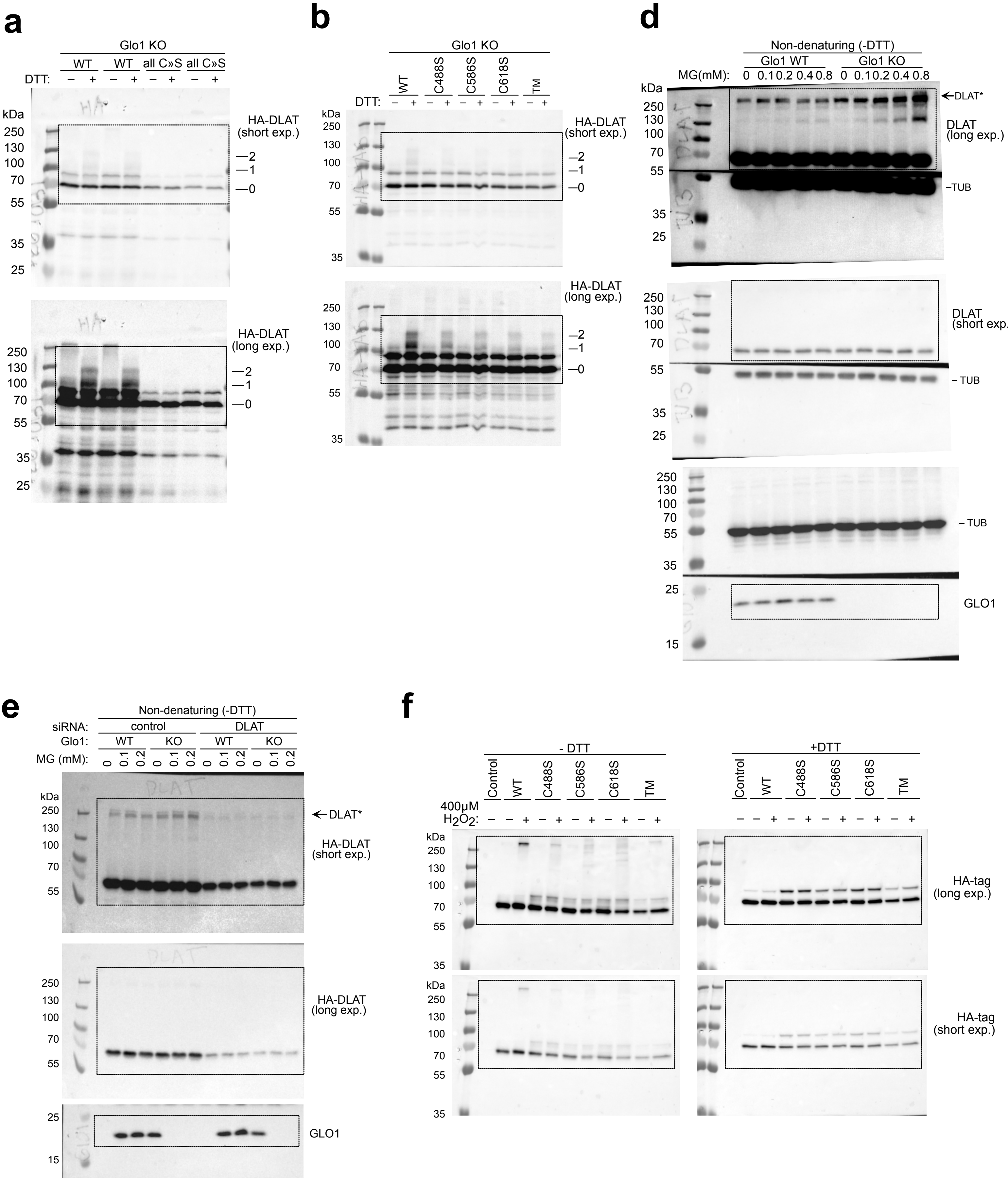

a
Glo1 KO
WT
WT
all C»S
all C»S
DTT:
kDa
250
130
100
70
55
35
25
HA-DLAT (short exp.)
HA-DLAT (long exp.)
—2
—1
—0
b
Glo1 KO
WT
C488S
C586S
C618S
TM
DTT:
HA-DLAT (short exp.)
HA-DLAT (long exp.)
d
Non-denaturing (-DTT)
Glo1 WT
Glo1 KO
MG(mM): 0 0.1 0.2 0.4 0.8 0 0.1 0.2 0.4 0.8
DLAT*
DLAT (long exp.)
TUB
DLAT (short exp.)
GLO1
e
Non-denaturing (-DTT)
siRNA:
control
DLAT
Glo1:
WT
KO
MG (mM):
DLAT*
HA-DLAT (short exp.)
HA-DLAT (long exp.)
GLO1
f
- DTT
+DTT
Control
WT
C488S
C586S
C618S
TM
400μM H2O2:
HA-tag (long exp.)
HA-tag (short exp.)
