# Supplementary material for: Regulation of PDH via cysteine oxidation on DLAT
Source: Nat Commun. 2026 Oct 14;17:10784. doi: 10.1038/s41467-026-78266-6 (PMC13669365; doi:10.1038/s41467-026-78266-6)
Supplement: Supplementary file 5 [file 41467_2026_78266_MOESM8_ESM.zip › Uncropped Blots Figure 4.pdf]

Figure 4

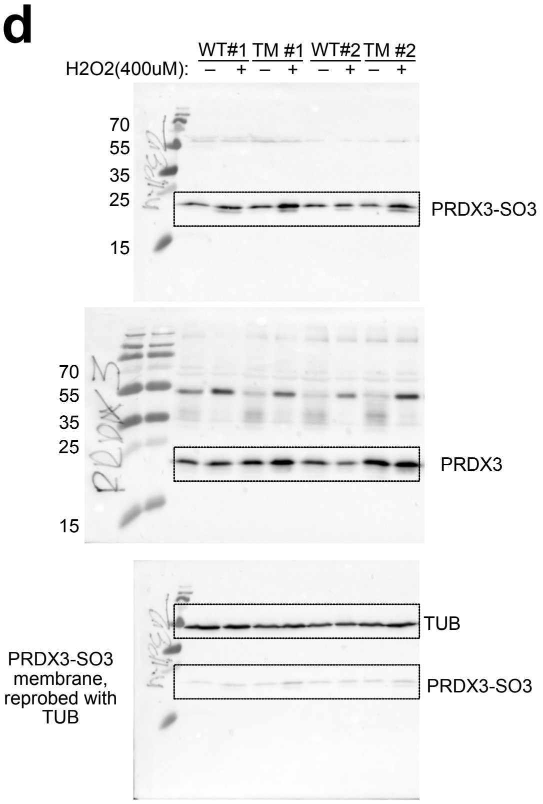

d
WT#1
TM #1
WT#2
TM #2
H2O2(400uM):
– + – + – + – +
70
55
35
25
15
PRDX3-SO3
PRDX3
TUB
PRDX3-SO3 membrane, reprobed with TUB
