# Supplementary material for: Regulation of PDH via cysteine oxidation on DLAT
Source: Nat Commun. 2026 Oct 14;17:10784. doi: 10.1038/s41467-026-78266-6 (PMC13669365; doi:10.1038/s41467-026-78266-6)
Supplement: Supplementary file 5 [file 41467_2026_78266_MOESM8_ESM.zip › Uncropped Blots Figure 5.pdf]

Figure 5

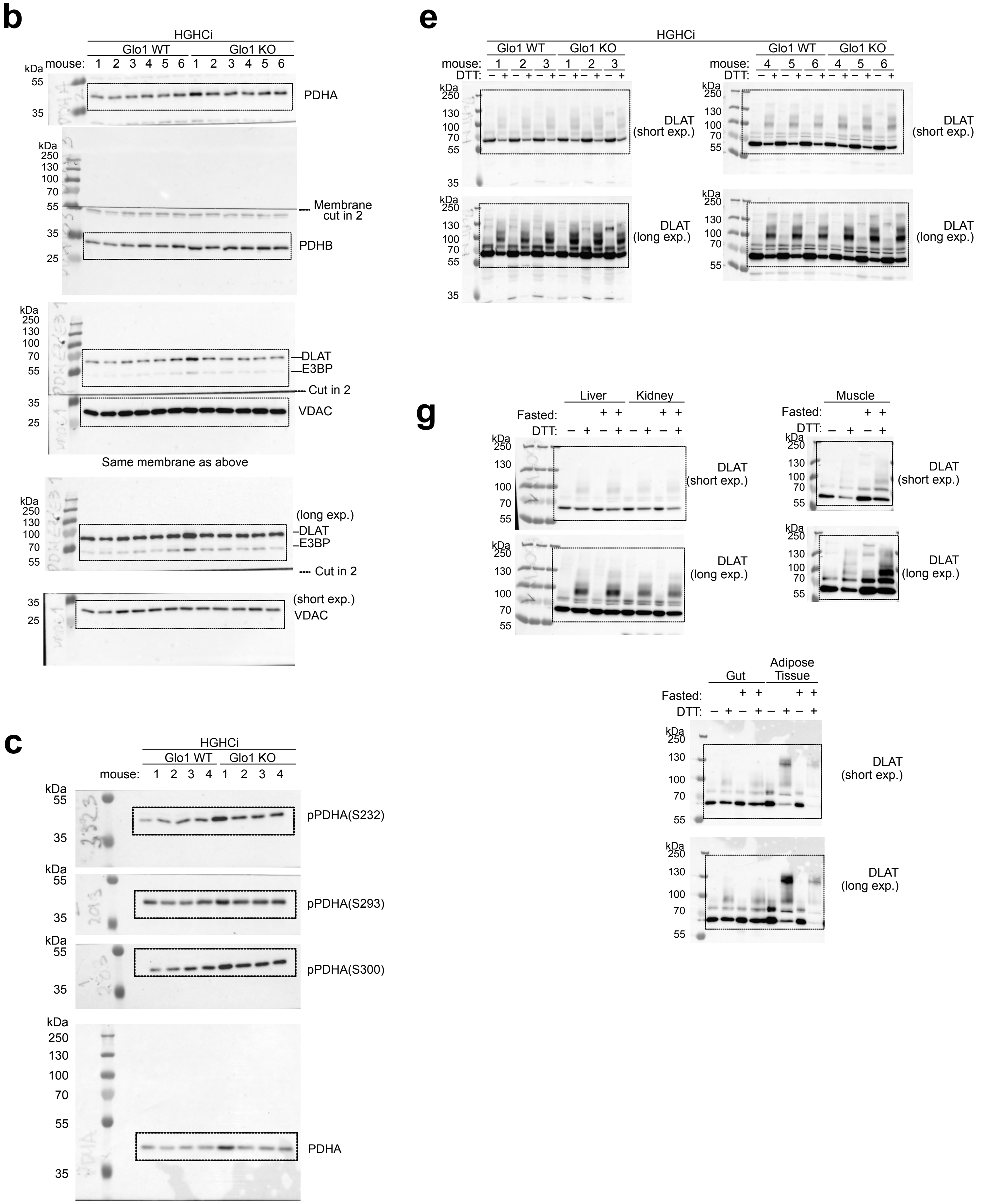

b
HGHCi
Glo1 WT
Glo1 KO
mouse: 1 2 3 4 5 6 1 2 3 4 5 6
kDa
PDHA
Membrane cut in 2
PDHB
DLAT
E3BP
Cut in 2
VDAC
Same membrane as above
(long exp.)
(short exp.)
c
HGHCi
Glo1 WT
Glo1 KO
mouse: 1 2 3 4 1 2 3 4
pPDHA(S232)
pPDHA(S293)
pPDHA(S300)
PDHA
e
HGHCi
Glo1 WT
Glo1 KO
mouse: 1 2 3 1 2 3
mouse: 4 5 6 4 5 6
DTT:
DLAT (short exp.)
DLAT (long exp.)
g
Liver
Kidney
Muscle
Gut
Adipose Tissue
Fasted:
DTT:
DLAT (short exp.)
DLAT (long exp.)
