# Supplementary material for: Regulation of PDH via cysteine oxidation on DLAT
Source: Nat Commun. 2026 Oct 14;17:10784. doi: 10.1038/s41467-026-78266-6 (PMC13669365; doi:10.1038/s41467-026-78266-6)
Supplement: Supplementary file 5 [file 41467_2026_78266_MOESM8_ESM.zip › Uncropped Blots Figure 6.pdf]

Figure 6

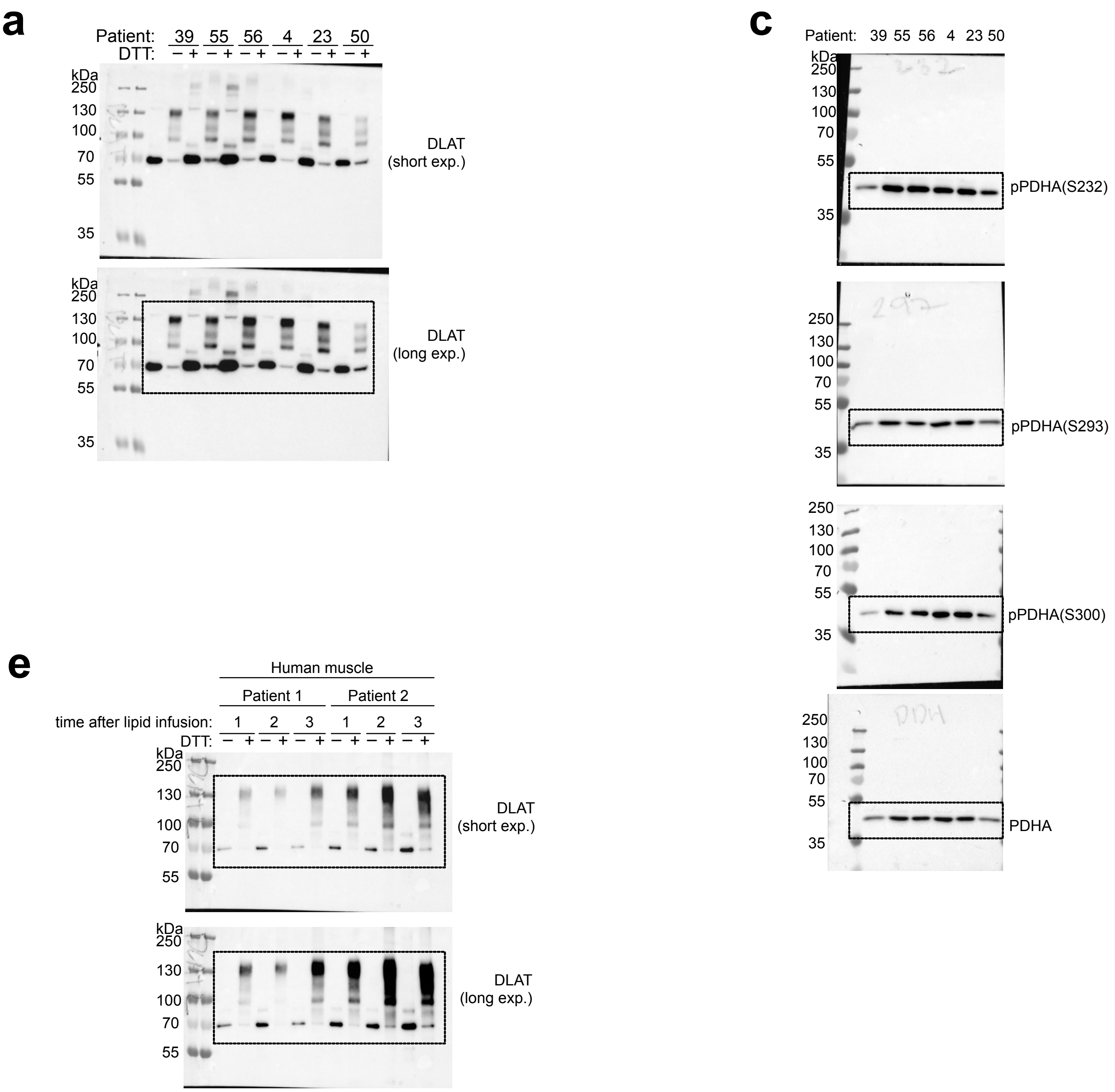
a
Patient: 39 55 56 4 23 50
DTT: − + − + − + − + − + − +
kDa
250
130
100
70
55
35
DLAT (short exp.)
DLAT (long exp.)
c
Patient: 39 55 56 4 23 50
pPDHA(S232)
pPDHA(S293)
pPDHA(S300)
PDHA
e
Human muscle
Patient 1
Patient 2
time after lipid infusion: 1 2 3 1 2 3
DTT: − + − + − + − + − + − +
DLAT (short exp.)
DLAT (long exp.)
